# Doebner-type pyrazolopyridine carboxylic acids in an Ugi four-component reaction

**DOI:** 10.3762/bjoc.15.126

**Published:** 2019-06-12

**Authors:** Maryna V Murlykina, Oleksandr V Kolomiets, Maryna M Kornet, Yana I Sakhno, Sergey M Desenko, Victoriya V Dyakonenko, Svetlana V Shishkina, Oleksandr A Brazhko, Vladimir I Musatov, Alexander V Tsygankov, Erik V Van der Eycken, Valentyn A Chebanov

**Affiliations:** 1 Division of Chemistry of Functional Materials, State Scientific Institution “Institute for Single Crystals” of National Academy of Sciences of Ukraine, Nauky Ave., 60, 61072, Kharkiv, Ukraine; 2 Faculty of Chemistry, V. N. Karazin Kharkiv National University, Svobody sq., 4, 61077, Kharkiv, Ukraine; 3 Laboratory for Organic & Microwave-Assisted Chemistry (LOMAC), KU Leuven, Celestijnenlaan 200F, B-3001, Leuven, Belgium; 4 Laboratory of Biotechnology of Physiologically Active Substances, Zaporizhzhia National University, Zhukovsky str., 66, Zaporizhzhya, Ukraine, 69600; 5 National Technical University “Kharkiv Polytechnic Institute”, Kyrpychova str., 2, 61002, Kharkiv, Ukraine; 6 Peoples' Friendship University of Russia, Miklukho-Maklya str., 6, 117198, Moscow, Russia

**Keywords:** antibacterial activity, Doebner reaction, pyrazolo[3,4-*b*]pyridine carboxylic acids, Ugi reaction

## Abstract

Substituted 1*H*-pyrazolo[3,4-*b*]pyridine-4- and 1*H*-pyrazolo[3,4-*b*]pyridine-6-carboxamides have been synthetized through a Doebner–Ugi multicomponent reaction sequence in a convergent and versatile manner using diversity generation strategies: combination of two multicomponent reactions and conditions-based divergence strategy. The target products contain as pharmacophores pyrazolopyridine and peptidomimetic moieties with four points of diversity introduced from readily available starting materials including scaffold diversity. A small focused compound library of 23 Ugi products was created and screened for antibacterial activity.

## Introduction

**Scheme 1:**
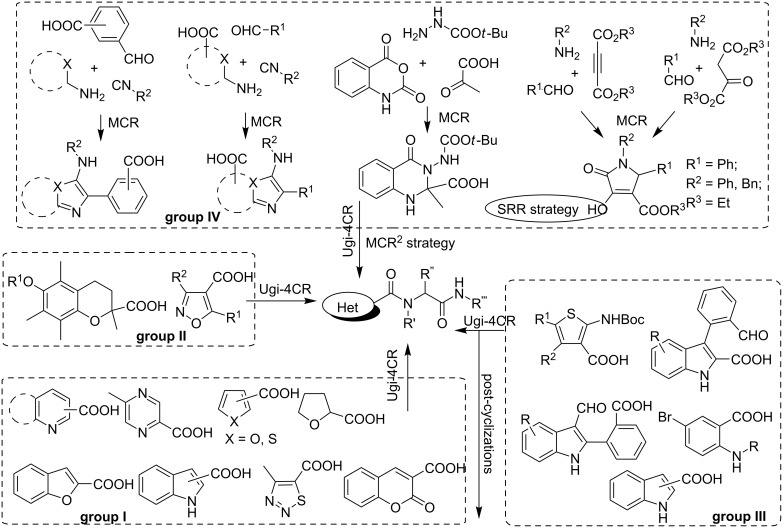
An overview of heterocyclic acids used in the Ugi reaction.

Modern medicinal chemistry is faced with the task of quick and effective screening a variety of organic molecules in order to identify new active pharmaceutical ingredients among them. Therefore, in turn, organic chemistry has to solve an equally important task of the rapid generating focused libraries of drug-like compounds characterized by several important features, e.g., molecular complexity and diversity at different levels, high variability and easy accessibility from relatively simple reagents. These challenges can be overcome by using multicomponent reactions (MCRs) but also other strategies can be applied in addition to MCRs for generating diversity, e.g., build/couple/pair- (BCP), single reactant replacement- (SRR), modular reaction sequences- (MRS), conditions-based divergence- (CBD) and combination of multicomponent reactions (MCR^2^) strategies (for more details and examples see [1–3] and Scheme 1). A synergetic application of several diversity-oriented synthesis (DOS) instruments allows an effective decoration of the privileged scaffolds for creating collections of unique, highly potent bioactive compounds [4–5].

The pyrazolopyridine scaffold can be regarded as a privileged motif as it exhibits various biological actions: antiproliferative [6–9], antimicrobial [10–11], anxiolytic [12], analgesic [13], hypnotic [13], antiviral [13], anti-HIV [13] activities, etc. Soural et al. [14] explored different data and showed the relevance of compounds composed of two or more heterocyclic rings for drug discovery. The target products containing a heterocyclic core bound to a peptide-like chain also showed a broad spectrum of biological activity: β-secretase (BACE1) inhibitory activity [15]; inducing apoptosis in colorectal cancer cells [16]; antimalarial activity against a chloroquine (CQ) non-resistant Plasmodium falciparum 3D7 strain [17]; antagonists of p53-Mdm2 interaction [18]; antiproliferative activity in the human solid tumor cell lines A549 (lung), HBL-100 (breast), HeLa (cervix), SW1573 (lung), T-47D (breast), and WiDr(colon) [19]; cyclophilin A inhibitory activity for the treatment of hepatitis C virus infections [20], etc.

Among the variety of heterocyclic acids used in Ugi-4CR [15–19,21–42] only a few of them in addition to bearing a simple pharmacophore core (group I, Scheme 1) are also characterized by the complexity and diversity of the skeleton itself gained through multi-step transformations (group II) [18–19,34–36] or allow for generating additional diversity through post-cyclization reactions (group III) [18,35–42]. Meanwhile the complexity of the acid skeleton can be achieved by MCR. Several publications illustrated this principle: synthesis of heterocyclic acids [26,43] or enols [44] in a first multicomponent step, followed by subjecting them to a subsequent Ugi process, thus, applying the MCR^2^ approach (group IV, Scheme 1).

Actually, there was no example for the combined application of Doebner and Ugi-type MCRs although the former condensation easily affords the azoloazine pharmacophore that is able to participate as an acid component in the latter reaction. It should be noted, that Cowen et al. [6] reported N-substituted-1*H*-pyrazolo[3,4-*b*]pyridine-4-carboxamides being SMYD2 inhibitors (an oncogenic methyltransferase that represses the functional activity of the tumor suppressor proteins p53 and RB); the similar structures can be obtained using the methodology of sequential Doebner- and Ugi-type MCRs.

In the present work we combined several diversity-oriented synthetic (DOS) approaches. First, by using CBD and MCR strategies in a Doebner-type reaction we synthesized pyrazolopyridine carboxylic acids which were subsequently applied in the Ugi reaction, thus, combining two multicomponent procedures.

## Results and Discussion

As mentioned above, the diversification of the privileged scaffold using different DOS strategies allowed to significantly increase the diversity of the final products. In our study pyrazolo[3,4-*b*]pyridine scaffold was chosen as a privileged one and based on this, we combined two MCRs: the previously well-studied three-component Doebner-type condensation of aminopyrazoles, aldehydes and pyruvic acid [45–46] with the isocyanide-based four-component Ugi reaction.

As we have shown before [47–48] the application of the CBD strategy to multicomponent Doebner-type condensations involving aminoazoles allowed the synthesis of several chemotypes of structurally complex products from a limited number of relatively simple starting materials just by varying the reaction conditions (temperature, solvent–catalyst system, activation method, forced realization of one of the cascades of multicomponent treatment). We decided to use this strategy and to synthetize heteroaromatic carboxylic acids **4** and **7** starting from the same reactants but using a multicomponent and a sequential protocol. We chose these heterocyclic acids to be subjected to the further Ugi transformation based on their higher stability compared to other azoloazine carboxylic acids (e.g., tetrahydro- [49–50] and dihydroazoloazines that may undergo oxidation during the Ugi 4CR) and as they do not contain additional functional groups that may influence the subsequent Ugi reaction (e.g., hydroxy group in tetrahydro- [46,51], dihydro- [51] or aromatic derivatives [51]).

**Scheme 2:**
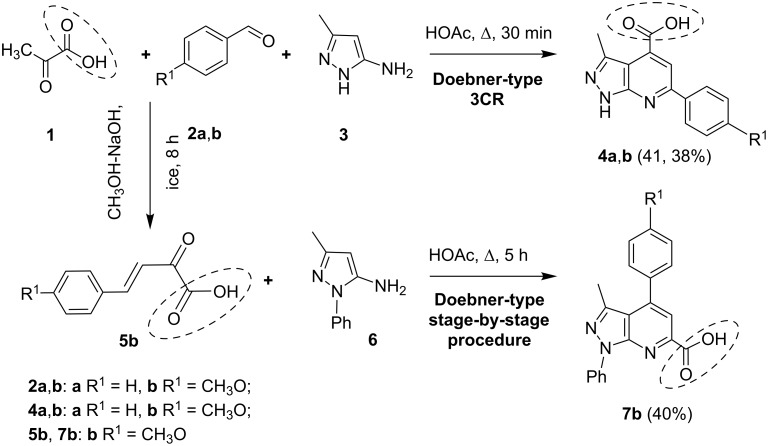
Synthesis of pyrazolopyridine carboxylic acids **4** [45] and **7** [45] in Doebner-type reaction.

Two different reaction pathways were applied based on known synthetic procedures (Scheme 2): the three-component reaction between pyruvic acid (**1**), aromatic aldehydes **2a**,**b** and 5-amino-3-methylpyrazole (**3**) (HOAc, Δ, 30 min) [45] and a two-component condensation of the preliminary synthetized 4-(4-methoxyphenyl)-2-oxobut-3-enoic acid (**5b**) [52–53] with 5-amino-3-methyl-*N*-phenylpyrazole (**6**) (HOAc, Δ, 5 h). As a result, two different types of pyrazolo[3,4-*b*]pyridines containing the carboxylic group either at C4 position (compounds **4a**,**b**) or at the C6 position (compound **7b**) were synthetized (Scheme 2). We modified the earlier described methodology for the synthesis of pyrazolo[3,4-*b*]pyridine-6-carboxylic acid (**7b**) [45]: the solvent was changed from DMF to HOAc and the reaction time was increased from 30 min to 5 hours. Despite of the longer reaction time the whole procedure became more efficient due to the easier work-up stage as well as due to avoiding the formation of impurities of the dihydropyrazolo[3,4-*b*]pyridines.

Thus, starting from the same set of reactants two different types of heterocyclic acids **4** and **7** containing two diversity points were obtained. Afterwards compounds **4a**,**b** were introduced into the Ugi four-component reaction to create 3 additional points of diversity. However, due to the low solubility of the pyrazolopyridine acids **4a**,**b** under the literature standard reaction conditions for the Ugi transformation (stirring in methanol at rt and similar procedures) the reaction did not take place. Under these conditions, the pyrazolopyridine carboxylic acids **4a,b** did not dissolve and remained unreacted even after prolonged stirring and heating. Consequently, the solvent was changed to DMF that allowed us to isolate the Ugi products **11** after long stirring (48–72 h) at rt. It must be noted, that in many cases the pyrazolopyridine acids **4a,b** did not fully dissolve in DMF at rt that resulted in considerably decreased yields.

In an attempt to increase the yield of the products the reaction was repeated at different temperatures ranging from rt to 80 °C and it was found that heating at 70 °C afforded the best results. At this temperature not only the yields increased but also the reaction time could be reduced to 48 hours. When applying a solvent mixture of DMF and MeOH the yields further increased, with the best results obtained using a ratio of 1:2. We presume that methanol provides the optimal acidity to the reaction medium needed for successful protonation of the intermediate azomethine, formed between the aromatic aldehyde **8** and aniline **9**, to the corresponding iminium cation and its further transformation involving carboxylic acid **4** and isocyanide **10**.

As a result, we developed an efficient procedure for the synthesis of compounds **11a–q** through reaction of aromatic aldehydes **8a–d**, amines **9a–f**, *tert*-butylisocyanide (**10**) and heteroaromatic carboxylic acids **4a**,**b** in a 2:1 mixture of methanol and DMF at 70 °C. Following this procedure, a small library of seventeen Ugi products was obtained (Table 1).

**Table 1:** Synthesis of compounds **11** and **12** by combination of Doebner and Ugi-type MCRs.

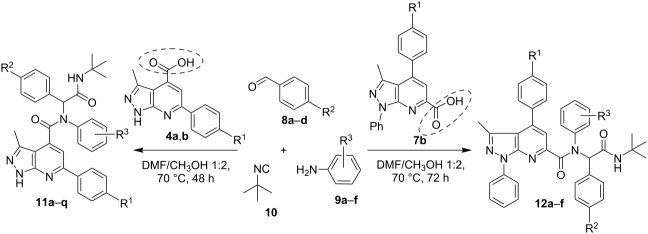								

	Starting materials						Products	

Entry	Acid	R^1^	**8**	R^2^	**9**	R^3^	**11**,**12**	yield, %

1	**4a**	H	**8a**	H	**9a**	H	**11a**	39
2	**4a**	H	**8a**	H	**9b**	4-CH_3_	**11b**	40
3	**4a**	H	**8a**	H	**9c**	4-Br	**11c**	30
4	**4a**	H	**8a**	H	**9d**	2-CH_3_O	**11d**	28
5	**4a**	H	**8a**	H	**9e**	3-CH_3_O	**11e**	30
6	**4a**	H	**8a**	H	**9f**	4-CH_3_O	**11f**	42
7	**4b**	CH_3_O	**8a**	H	**9a**	H	**11g**	43
8	**4b**	CH_3_O	**8a**	H	**9b**	4-CH_3_	**11h**	53
9	**4b**	CH_3_O	**8a**	H	**9c**	4-Br	**11i**	37
10	**4b**	CH_3_O	**8a**	H	**9d**	2-CH_3_O	**11j**	37
11	**4b**	CH_3_O	**8a**	H	**9e**	3-CH_3_O	**11k**	35
12	**4b**	CH_3_O	**8a**	H	**9f**	4-CH_3_O	**11l**	42
13	**4b**	CH_3_O	**8b**	Cl	**9a**	H	**11m**	44
14	**4b**	CH_3_O	**8b**	Cl	**9b**	4-CH_3_	**11n**	49
15	**4b**	CH_3_O	**8b**	Cl	**9c**	4-Br	**11o**	34
16	**4b**	CH_3_O	**8b**	Cl	**9f**	4-CH_3_O	**11p**	37
17	**4b**	CH_3_O	**8c**	NO_2_	**9a**	H	**11q**	20
18	**7b**	CH_3_O	**8a**	H	**9a**	H	**12a**	50
19	**7b**	CH_3_O	**8a**	H	**9b**	4-CH_3_	**12b**	51
20	**7b**	CH_3_O	**8b**	Cl	**9a**	H	**12c**	34
21	**7b**	CH_3_O	**8b**	Cl	**9b**	4-CH_3_	**12d**	36
22	**7b**	CH_3_O	**8d**	CH_3_O	**9a**	H	**12e**	46
23	**7b**	CH_3_O	**8d**	CH_3_O	**9b**	4-CH_3_	**12f**	25

Next, we applied pyrazolo[3,4-*b*]pyridine-6-carboxylic acid **7b** with another positional location of the substituents in comparison with compounds **4** in the Ugi reaction with the same reagents **8**, **9** and **10** using the optimized procedure (Table 1). This expanded the library of Ugi products by adding compounds **12a–f**.

**Figure 1:**
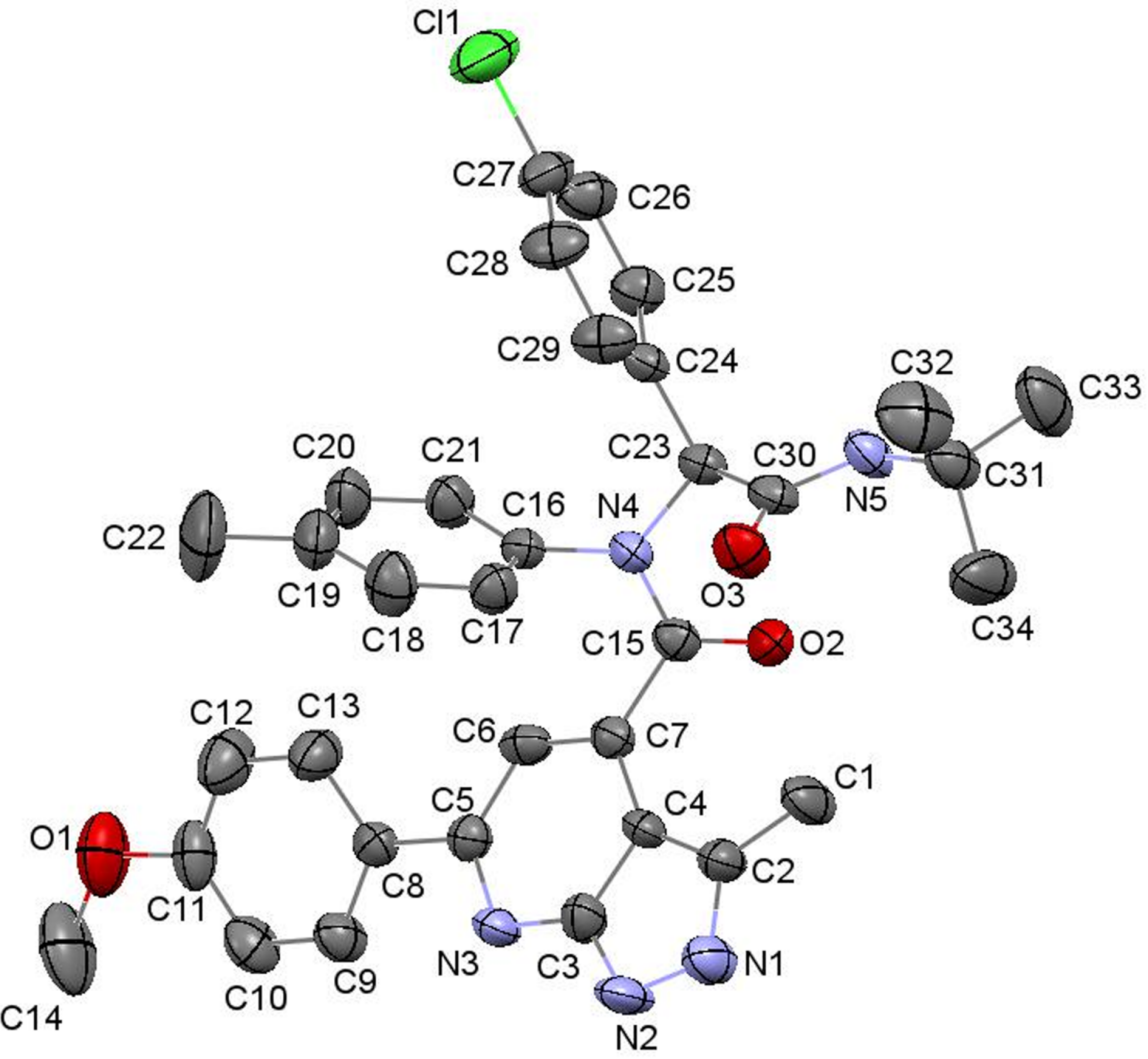
Molecular structure of *N*-(2-(*tert*-butylamino)-1-(4-chlorophenyl)-2-oxoethyl)-6-(4-methoxyphenyl)-3-methyl-*N*-*p*-tolyl-1*H*-pyrazolo[3,4-*b*]pyridine-4-carboxamide (**11n**) according to X-ray diffraction data. Non-hydrogen atoms are presented as thermal ellipsoids with 50% probability.

The purity and structures of the obtained heterocyclic products were established by means of NMR spectroscopy, mass spectrometry, and elemental analysis. The final assignment of the structures **11** and **12** was made by X-ray analysis for the structure **11n** (Figure 1).

## Antibacterial activity

It is worth mentioning that the modification of the pyrazolo[3,4-*b*]pyridine scaffold through Ugi reaction allowed not only to introduce three additional diversity points but also to increase significantly the solubility of the products **11** and **12** compared to the starting acids **4** and **7**. Compounds **11** are soluble in MeOH, EtOH, iPrOH, acetone, EtOAc, CH_3_CN, DCM, CHCl_3_ and compounds **12** are soluble in acetone, CH_3_CN, DCM, CHCl_3_ and partially soluble when heated in EtOAc, MeOH, EtOH, showing the advantages of this protocol for investigating the activity of pyrazolo[3,4-*b*]pyridine moiety in biological experiments. Particularly, the evaluation of the antibacterial activity of the small library of new compounds **11** and **12** was carried out.

We next screened some selected compounds for their antibacterial activity (Table 2, Supporting Information File 1) against the reference bacterial strains *Bacillus subtilis* (strain 1211), *Staphylococcus aureus* (strain 2231) (gram-positive) and *Escherichia coli* (strain 1257), *Pseudomonas aeruginosa* (strain 1111) (gram-negative).

**Table 2:** Antibacterial activity results.

Entry	Compound	MIC^a^/MBC^b^, mg/L	Strains of test cultures			

			*Escherichia coli*	*Pseudomonas aeruginosa*	*Staphylococcus aureus*	*Bacillus subtilis*

1	**11a**	MIC	250	–^c^	250	250
		MBC	–	–	–	–
2	**11b**	MIC	500	–	500	125
		MBC	–	–	–	–
3	**11f**	MIC	–	–	–	–
		MBC	–	–	–	–
4	**11g**	MIC	250	–	–	250
		MBC	–	–	–	–
5	**11l**	MIC	–	–	–	–
		MBC	–	–	–	–
6	**11m**	MIC	500	–	250	250
		MBC	–	–	–	–
7	**nitroxoline**	MIC	15.6	62.5	31.25	1.9
		MBC	15.6	62.5	31.25	1.9

^a^MIC – minimum inhibitory concentration; ^b^MBC – minimum bactericidal concentration; ^c^the substance at concentration ≤ 500 mg/L does not inhibit culture growth.

Generally, the compounds were found to be less active than nitroxoline being the reference substance. The results obtained indicate that some substances inhibited the growth of the test microorganisms demonstrating weak antimicrobial effect (Table 2). The growth of gram-positive bacteria (strains of *S. aureus* and *B. subtilis*) was inhibited in a more effective way. Particularly, compound **11b** inhibited the growth of *B. subtilis* at a concentration of 125 mg/L. A bacteriostatic activity against *S. aureus* was observed only at the higher concentrations of 250 and 500 mg/L. The same situation was found for the tested *E. coli* strain. The gram-negative bacterium *P. aeruginosa* showed resistance to all compounds tested in the given concentration range. The observed low level of antibacterial activity of the synthesized heterocycles is a good prerequisite for screening them for other types of activity, e.g., anticancer, antidiabetic, etc., because in these cases a negative influence on the microflora of the organism would be decreased [54].

## Conclusion

In summary, two multicomponent reactions of Doebner and Ugi-type were combined in a convergent and versatile manner giving substituted 1*H*-pyrazolo[3,4-*b*]pyridine-4- and 1*H*-pyrazolo[3,4-*b*]pyridine-6-carboxamides. The use of a conditions-based divergence strategy allowed introducing the scaffold diversity and obtaining two types of structures with different orientation of substituents (containing a carboxylic group either at C4 or C6 position of the pyrazolopyridine core). The optimal methodology for the synthesis of target products was elaborated (mixture of methanol and DMF (2:1) and heating to 70 °C) and a small focused library of 23 Ugi products was created. The target compounds containing two pharmacophore pyrazolopyridine and peptidomimetic moieties were screened for their antibacterial activity and demonstrated weak antibacterial effect.

## Supporting Information

File 1 Experimental and analytical data.

File 2 NMR spectra.
