# Supplementary material for: Doebner-type pyrazolopyridine carboxylic acids in an Ugi four-component reaction
Source: Beilstein J Org Chem. 2019 Jun 12;15:1281–8. doi: 10.3762/bjoc.15.126 (PMC6604699; doi:10.3762/bjoc.15.126)
Supplement: File 1 — Experimental and analytical data. [file Beilstein_J_Org_Chem-15-1281-s001.pdf]

BEILSTEIN JOURNAL OF ORGANIC CHEMISTRY

Supporting Information

for

Doebner-type pyrazolopyridine carboxylic acids in an Ugi four-component reaction

Maryna V. Murlykina, Oleksandr V. Kolomiets, Maryna M. Kornet, Yana I. Sakhno, Sergey M. Desenko, Victoriya V. Dyakonenko, Svetlana V. Shishkina, Oleksandr A. Brazhko, Vladimir I. Musatov, Alexander V. Tsygankov, Erik V. Van der Eycken and Valentyn A. Chebanov

Beilstein J. Org. Chem. **2019,** *15,* 1281–1288. doi:10.3762/bjoc.15.126

Experimental and analytical data

License and Terms: This is a supporting information file under the terms of the Creative Commons Attribution License (http://creativecommons.org/licenses/by/4.0). Please note that the reuse, redistribution and reproduction in particular requires that the authors and source are credited.
The license is subject to the *Beilstein Journal of Organic Chemistry* terms and conditions: (https://www.beilstein-journals.org/bjoc)

1. General

The starting 6-aryl-3-methyl-1*H*-pyrazolo[3,4-*b*]pyridine-4-carboxylic acids **4** [1], 4-(4-methoxyphenyl)-2-oxobut-3-enoic acid (**5b**) [2,3], 4-(4-methoxyphenyl)-3-methyl-1-phenyl-1*H*-pyrazolo[3,4-*b*]pyridine-6-carboxylic acid (**7b**) [1] were synthesized according to the known literature procedures. *tert*-Butylisocyanide and substituted aldehydes were commercially available.

Melting points of all compounds synthesized were determined with a Kofler melting point apparatus and were uncorrected. The NMR spectra were recorded in DMSO-d_6 at 300 MHz (75 MHz for ^{13}C) with a Bruker Avance 300 spectrometer, at 400 MHz (100 MHz for ^{13}C) with a Varian MR-400. The mass spectra were recorded on LCQ Advantage system for LC–MS and direct injections coupled to an ELSD detector, Agilent 6120 Quadrupole LCMS System. Elemental analysis was realized on EA-3000 CHNS-O analyzer (Eurovector, Italy).

Ultrasonic-assisted experiments were performed using the standard US-baths (SELDI, Ukraine) with a working frequency of 44.2 kHz and Bransonic® ultrasonic cleaner Branson 2510EDTH with a working frequency 42 kHz.

2. Chemistry

General procedure for the synthesis of *N*-(2-(*tert*-butylamino)-2-oxo-1-arylethyl)-*N*,6-diaryl-3-methyl-1*H*-pyrazolo[3,4-*b*]pyridine-4-carboxamides 11a–q.

The appropriate aniline **9** (0.5 mmol) and aromatic aldehyde **8** (0.5 mmol) were dissolved in DMF/CH_3OH 1:2 (2 mL) and stirred for 1 hour. Then, 3-methyl-6-aryl-1*H*-pyrazolo[3,4-*b*]pyridine-4-carboxylic acid **4** (0.5 mmol) and *tert*-butylisocyanide (**10**, 0.5 mmol) were added consecutively and the reaction mixture was stirred for 48 h at 70 °C in a closed vial untill completion. Afterwards, the mixture was poured onto ice, ultrasonicated for 15–30 min, and

filtered. The collected precipitate was treated with 5 mL of EtOAc/hexane 1:15, ultrasonicated for additional 15–30 min, filtered again and dried.

*N-(2-(tert-Butylamino)-2-oxo-1-phenylethyl)-3-methyl-N,6-diphenyl-1H-pyrazolo[3,4-b]pyridine-4-carboxamide (**11a**)*. Corless solid, mp 211-212 °C. [Found: C, 74.14%; H, 6.10%; N, 13.54. $C_{32}H_{31}N_5O_2$ requires C, 74.25%; H, 6.04%; N, 13.53 %]; ^{1}H NMR (300 MHz, DMSO-d$_6$, δ, ppm): 13.28 (s, 1H, NH), 7.99 (s, 1H, NH), 6.92-7.91 (m, 15H, ArH), 7.37 (s, 1H, CH), 6.31 (s, 1H, CH), 2.61 (s, 3H, CH_3), 1.32 (c, 9H, $\underline{(CH_3)_3}C$); NMR ^{13}C (75 MHz, DMSO-d$_6$, δ, ppm): 168.8, 166.6, 154.4, 153.0, 140.6, 139.1, 139.0, 138.2, 134.9, 131.6, 130.28, 129.4, 128.7, 127.8, 127.5, 127.3, 126.8, 111.7, 109.3, 64.0, 50.4, 28.4, 13.4. LC/MS (ESI): 518 [MH]$^+$.

*N-(2-(tert-Butylamino)-2-oxo-1-phenylethyl)-3-methyl-6-phenyl-N-p-tolyl-1H-pyrazolo[3,4-b]pyridine-4-carboxamide (**11b**)*. Colorless solid, mp 167-168 °C. [Found: C, 74.37%; H, 6.34%; N, 13.22%. $C_{33}H_{33}N_5O_2$ requires C, 74.55%; H, 6.25%; N, 13.17 %]; ^{1}H NMR (300 MHz, DMSO-d$_6$, δ, ppm): 13.27 (s, 1H, NH), 7.96 (s, 1H, NH), 6.62-7.94 (m, 14H, ArH), 7.39 (s, 1H, CH), 6.28 (s, 1H, CH), 2.59 (s, 3H, CH_3), 1.91 (s, 3H, CH_3), 1.32 (s, 9H, $\underline{(CH_3)_3}C$); NMR ^{13}C (75 MHz, DMSO-d$_6$, δ, ppm): 168.8, 166.8, 140.5, 139.3, 138.2, 136.4, 136.4, 135.0, 131.3, 130.3, 129.4, 128.9, 128.8, 128.1, 127.8, 127.0, 126.9, 111.6, 109.3, 64.0, 50.4, 28.4, 20.3, 13.4. LC/MS (ESI): 532 [MH]$^+$.

*N-(4-Bromophenyl)-N-(2-(tert-butylamino)-2-oxo-1-phenylethyl)-3-methyl-6-phenyl-1H-pyrazolo[3,4-b]pyridin-4-carboxamide (**11c**)*. Colorless solid, mp139-141 °C. [Found: C, 64.42%; H, 5.10; N, 11.65. $C_{32}H_{30}BrN_5O_2$ requires C, 64.43%; H, 5.07%; N, 11.74 %]; ^{1}H NMR (300 MHz, DMSO-d$_6$, δ, ppm): 13.30 (s, 1H, NH), 7.95 (s, 1H, NH), 7.41-8.05 (m, 14H, ArH), 7.45 (s, 1H, CH), 6.31 (s, 1H, CH), 2.59 (s, 3H, CH_3), 1.32 (s, 9H, $\underline{(CH_3)_3}C$); NMR ^{13}C (75 MHz, DMSO-d$_6$, δ, ppm): 168.8, 166.5, 154.5, 153.0, 140.5, 138.8, 138.5, 138.1, 134.6,

133.8, 130.5, 130.2, 129.5, 128.8, 128.0, 126.9, 120.5, 111.6, 109.2, 99.5, 63.8, 50.5, 28.3, 13.4. LC/MS (ESI): 596 $[MH]^+$.

*N-(2-(tert-butylamino)-2-oxo-1-phenylethyl)-3-methyl-6-phenyl-N-(2-methoxyphenyl)-1H-pyrazolo[3,4- b]pyridin-4-carboxamide (**11d**).* Colorless solid, mp 164-166 °C. [Found: C, 72.35%; H, 6.13; N, 12.63. $C_{33}H_{33}N_5O_3$ C, 72.37%; H, 6.07; N, 12.79 %]; ^{1}H NMR (300 MHz, DMSO-d_6, δ, ppm): 13.28 (s, 1H, NH), 7.92 (s, 1H, NH), 6.38-7.84 (m, 14H, ArH), 7.25 (s, 1H, CH), 6.16 (s, 1H, CH), 3.38 (s, 3H, $\underline{CH_3}O$), 2.61 (s, 3H, CH_3), 1.31 (s, 9H, $\underline{(CH_3)_3}C$); NMR ^{13}C (75 MHz, DMSO-d_6, δ, ppm): 168.9, 166.8, 155.1, 154.2, 153.1, 140.8, 139.0, 138.3, 133.9, 132.3, 130.2, 129.6, 129.4, 128.9, 128.0, 127.7, 127.0, 126.6, 119.6, 110.7, 109.8, 109.2, 64.2, 54.7, 50.3, 28.4, 13.5. LC/MS (ESI): 548 $[MH]^+$.

*N-(2-(tert-Butylamino)-2-oxo-1-phenylethyl)-3-methyl-6-phenyl-N-(3-methoxyphenyl)-1H-pyrazolo[3,4-b]pyridin-4-carboxamide (**11e**).* Colorless solid, mp 137-139 °C. [Found: C, 72.38; H, 6.06; N, 12.78. $C_{33}H_{33}N_5O_3$ C, 72.37%; H, 6.07%; N, 12.79 %]; ^{1}H NMR (300 MHz, DMSO-d_6, δ, ppm): 13.28 (s, 1H, NH), 7.98 (s, 1H, NH), 6.32-7.96 (m, 14H, ArH), 7.21 (s, 1H, CH), 6.28 (s, 1H, CH), 3.39 (s, 3H, CH_3O), 2.60 (s, 3H, CH_3), 1.37 (s, 9H, $\underline{(CH_3)_3}C$); NMR ^{13}C (75 MHz, DMSO-d_6, δ, ppm): 168.8, 166.6, 158.2, 154.3, 152.9, 140.6, 140.1, 139.0, 138.2, 134.8, 130.3, 129.4, 128.7, 128.1, 127.8, 126.8, 123.9, 117.3, 113.3, 111.6, 109.4, 64.0, 54.8, 50.4, 28.4, 13.4. HRMS (EI) requires LC/MS (ESI): 548 $[MH]^+$.

*N-(2-(tert-Butylamino)-2-oxo-1-phenylethyl)-3-methyl-6-phenyl-N-(4-methoxyphenyl)-1H-pyrazolo[3,4-b]pyridin-4-carboxamide (**11f**).* Colorless solid, mp 141-143 °C. [Found: C, 72.27%; H, 6.14%; N, 12.82%. $C_{33}H_{33}N_5O_3$ C, 72.37%; H, 6.07%; N, 12.79 %]; ^{1}H NMR (300 MHz, DMSO-d_6, δ, ppm): 13.27 (s, 1H, NH), 7.96 (s, 1H, NH), 6.35-7.95 (m, 14H, ArH), 7.39 (s, 1H, CH), 6.27 (s, 1H, CH), 3.42 (s, 3H, CH_3O), 2.59 (s, 3H, CH_3), 1.32 (s, 9H, $\underline{(CH_3)_3}C$); NMR ^{13}C (75 MHz, DMSO-d_6, δ, ppm): 168.9, 166.9, 157.7, 154.4, 153.0, 140.6, 139.4, 138.3,

135.0, 132.7, 131.6, 130.3, 129.4, 128.8, 127.9, 127.8, 126.9, 112.6, 111.5, 109.3, 63.9, 54.8, 50.4, 28.4, 13.4. LC/MS (ESI): 548 $[MH]^+$.

N-(2-(tert-Butylamino)-2-oxo-1-phenylethyl)-3-methyl-6-(4-methoxyphenyl)-N-phenyl-1H-pyrazolo[3,4-b]pyridin-4-carboxamide (***11g***). Colorless solid, mp 186-187 °C. [Found: C, 72.30%; H, 6.11%; N, 12.76%. $C_{33}H_{33}N_5O_3$ C, 72.37%; H, 6.07; N, 12.79 %]; ^{1}H NMR (300 MHz, DMSO-d$_6$, δ, ppm): 13.17 (s, 1H, NH), 7.98 (s, 1H, NH), 6.82-7.88 (m, 14H, ArH), 7.29 (s, 1H, CH), 6.31 (s, 1H, CH), 3.80 (s, 3H, $\underline{CH_3}$O), 2.59 (s, 3H, CH_3), 1.32 (s, 9H, $\underline{(CH_3)_3}$C); NMR ^{13}C (75 MHz, DMSO-d$_6$, δ, ppm): 168.8, 166.7, 160.4, 154.2, 153.0, 140.5, 139.1, 138.9, 134.9, 131.5, 130.7, 130.3, 128.2, 127.8, 127.5, 127.2, 114.1, 111.1, 108.9, 64.0, 55.2, 50.4, 28.4, 13.4. LC/MS (ESI): 548 $[MH]^+$.

N-(2-(tert-Butylamino)-2-oxo-1-phenylethyl)-3-methyl-6-(4-methoxyphenyl)-N-p-tolyl-1H-pyrazolo[3,4-b]pyridin-4-carboxamide (***11h***). Colorless solid, mp 118-120 °C. [Found: C, 72.73%; H, 6.19%; N, 12.53%. $C_{34}H_{35}N_5O_3$ C, 72.71%; H, 6.28%; N, 12.47 %]; ^{1}H NMR (300 MHz, DMSO-d$_6$, δ, ppm): 13.17 (s, 1H, NH), 7.95 (s, 1H, NH), 6.64-7.91 (m, 13H, ArH), 7.33 (s, 1H, CH), 6.29 (s, 1H, CH), 3.81 (s, 3H, $\underline{CH_3}$O), 2.58 (s, 3H, CH_3), 1.92 (s, 3H, CH_3), 1.32 (s, 9H, $\underline{(CH_3)_3}$C); NMR ^{13}C (75 MHz, DMSO-d$_6$, δ, ppm): 168.8, 166.9, 160.4, 154.2, 153.0, 140.5, 139.1, 136.4, 136.3, 135.0, 131.3, 130.7, 130.3, 128.2, 128.0, 127.8, 114.1, 111.0, 108.8, 63.9, 55.2, 50.4, 28.4, 20.3, 13.4. LC/MS (ESI): 563 $[M+2H]^+$.

N-(4-Bromophenyl)-N-(2-(tert-butylamino)-2-oxo-1-phenylethyl)-3-methyl-6-(4-methoxyphenyl)-1H-pyrazolo[3,4-b]pyridine-4-carboxamide (***11i***). Colorless solid, mp 127-129 °C. [Found: C, 63.13%; H, 5.20%; N, 11.20%. $C_{33}H_{32}BrN_5O_3$ C, 63.26%; H, 5.15%; N, 11.18 %]; ^{1}H NMR (300 MHz, DMSO-d$_6$, δ, ppm): 13.23 (s, 1H, NH), 8.04 (s, 1H, NH), 7.01-7.93 (m, 13H, ArH), 7.39 (s, 1H, CH), 6.30 (s, 1H, CH), 3.81 (s, 3H, $\underline{CH_3}$O), 2.57 (s, 3H, CH_3), 1.32 (s, 9H, $\underline{(CH_3)_3}$C); NMR ^{13}C (75 MHz, DMSO-d$_6$, δ, ppm): 168.7, 166.5, 160.4, 154.3, 153.0,

138.6, 138.5, 134.6, 133.7, 130.6, 130.5, 130.5, 130.2, 128.3, 128.0, 120.5, 114.2, 111.0, 108.7, 63.8, 55.2, 50.4, 28.3, 13.3. LC/MS (ESI): 628 $[M+3H]^+$.

*N-(2-(tert-Butylamino)-2-oxo-1-phenylethyl)-3-methyl-6-(4-methoxyphenyl)-N-(2-methoxyphenyl)-1H-pyrazolo[3,4-b]pyridine-4-carboxamide (**11j**).* Colorless solid, mp 148-150 °C. [Found: C, 70.55%; H, 6.19%; N, 12.10%. $C_{34}H_{35}N_5O_4$ requires C, 70.69%; H, 6.11%; N, 12.12 %]; ^{1}H NMR (300 MHz, DMSO-d_6, δ, ppm): 13.17 (s, 1H, NH), 7.91 (s, 1H, NH), 6.40-7.76 (m, 13H, ArH), 7.19 (s, 1H, CH), 6.14 (s, 1H, CH), 3.80 (s, 3H, $\underline{CH_3}O$), 3.39 (s, 3H, $\underline{CH_3}O$), 2.59 (s, 3H, CH_3), 1.30 (s, 9H, $\underline{(CH_3)_3}C$); NMR ^{13}C (75 MHz, DMSO-d_6, δ, ppm): 168.9, 166.9, 160.3, 155.1, 154.0, 153.1, 140.7, 138.8, 133.9, 132.3, 130.7, 130.2, 129.6, 128.0, 127.9, 127.7, 127.0, 119.6, 114.2, 110.7, 109.2, 108.7, 64.2, 55.2, 54.7, 50.3, 28.4, 13.5. LC/MS (ESI): 578 $[MH]^+$.

*N-(2-(tert-Butylamino)-2-oxo-1-phenylethyl)-3-methyl-6-(4-methoxyphenyl)-N-(3-methoxyphenyl)-1H-pyrazolo[3,4-b]pyridine-4-carboxamide (**11k**).* Colorless solid, mp 122-124 °C. [Found: C, 70.66%; H, 6.04%; N, 12.15%. $C_{34}H_{35}N_5O_4$ requires C, 70.69%; H, 6.11%; N, 12.12 %]; ^{1}H NMR (300 MHz, DMSO-d_6, δ, ppm): 13.18 (s, 1H, NH), 7.97 (s, 1H, NH), 6.36-7.91 (m, 13H, ArH), 7.39 (s, 1H, CH), 6.27 (s, 1H, CH), 3.81 (s, 3H, $\underline{CH_3}O$), 3.38 (s, 3H, $\underline{CH_3}O$), 2.58 (s, 3H, CH_3), 1.32 (s, 9H, $\underline{(CH_3)_3}C$); NMR ^{13}C (75 MHz, DMSO-d_6, δ, ppm): 174.0, 171.9, 165.7, 163.4, 159.4, 158.2, 145.7, 145.4, 144.1, 140.1, 135.9, 135.6, 133.4, 133.4, 133.3, 133.1, 129.1, 122.5, 119.4, 116.2, 114.1, 69.2, 60.5, 60.1, 55.7, 33.6, 18.6. LC/MS (ESI): 578 $[MH]^+$.

*N-(2-(tert-Butylamino)-2-oxo-1-phenylethyl)-3-methyl-N,6-bis(4-methoxyphenyl)-1H-pyrazolo[3,4-b]pyridine-4-carboxamide (**11l**).* Colorless solid, mp 125-126 °C. [Found: C, 70.56%; H, 6.14%; N, 12.11%. $C_{34}H_{35}N_5O_4$ requires C, 70.69%; H, 6.11%; N, 12.12 %]; ^{1}H NMR (300 MHz, DMSO-d_6, δ, ppm): 13.17 (s, 1H, NH), 7.95 (s, 1H, NH), 6.40-7.91 (m,

13H, ArH), 7.32 (s, 1H, CH), 6.27 (s, 1H, CH), 3.81 (s, 3H, CH_3O), 3.43 (s, 3H, CH_3O), 2.57 (s, 3H, CH_3), 1.31 (s, 9H, $(CH_3)_3C$); NMR ^{13}C (75 MHz, DMSO-d_6, δ, ppm): 168.9, 167.0, 160.4, 157.6, 154.2, 153.0, 140.5, 139.2, 135.0, 132.6, 131.6, 130.7, 130.3, 128.3, 127.8, 127.8, 114.1, 112.5, 110.9, 108.8, 63.9, 55.2, 54.8, 50.4, 28.4, 13.4. LC/MS (ESI): 578 $[MH]^+$.

*N-(2-(tert-Butylamino)-1-(4-chlorophenyl)-2-oxoethyl)-6-(4-methoxyphenyl)-3-methyl-N-phenyl-1H-pyrazolo[3,4-b]pyridine-4-carboxamide (**11m**).* Colorless solid, mp 135-136 °C. [Found: C, 68.15%; H, 5.38%; N, 12.00%. $C_{33}H_{32}ClN_5O_3$ requires C, 68.09%; H, 5.54%; N, 12.03 %]; ^1H NMR (300 MHz, DMSO-d_6, δ, ppm): 13.18 (s, 1H, NH), 8.02 (s, 1H, NH), 6.87-7.88 (m, 13H, ArH), 7.31 (s, 1H, CH), 6.28 (s, 1H, CH), 3.80 (s, 3H, CH_3O), 2.58 (s, 3H, CH_3), 1.31 (s, 9H, $(CH_3)_3C$); NMR ^{13}C (75 MHz, DMSO-d_6, δ, ppm): δ 168.5, 166.7, 160.4, 154.2, 153.0, 140.5, 138.9, 138.7, 133.9, 132.6, 132.0, 131.5, 130.6, 128.2, 127.9, 127.7, 127.5, 114.1, 111.1, 108.8, 63.2, 55.2, 50.5, 28.3, 13.4. LC/MS (ESI): 583 $[M+2H]^+$.

*N-(2-(tert-Butylamino)-1-(4-chlorophenyl)-2-oxoethyl)-6-(4-methoxyphenyl)-3-methyl-N-p-tolyl-1H-pyrazolo[3,4-b]pyridine-4-carboxamide (**11n**).* Colorless solid, mp 213-215 °C. [Found: C, 68.34%; H, 5.40%; N, 11.85%. $C_{34}H_{34}ClN_5O_3$ requires C, 68.50%; H, 5.75%; N, 11.75 %]; ^1H NMR (300 MHz, DMSO-d_6, δ, ppm): 13.18 (s, 1H, NH), 7.99 (s, 1H, NH), 6.69-7.90 (m, 12H, ArH), 7.34 (s, 1H, CH), 6.27 (s, 1H, CH), 3.81 (s, 3H, CH_3O), 2.57 (s, 3H, CH_3), 1.94 (s, 3H, CH_3), 1.31 (s, 9H, $(CH_3)_3C$); NMR ^{13}C (75 MHz, DMSO-d_6, δ, ppm): 168.5, 166.9, 160.4, 154.1, 153.0, 140.4, 138.9, 136.6, 136.3, 134.0, 132.5, 132.0, 131.4, 131.3, 130.7, 128.4, 128.2, 127.9, 114.1, 111.0, 108.8, 63.1, 55.2, 50.4, 28.3, 20.3, 13.4. LC/MS (ESI): 596 $[MH]^+$.

*N-(4-Bromophenyl)-N-(2-(tert-butylamino)-1-(4-chlorophenyl)-2-oxoethyl)-6-(4-methoxyphenyl)-3-methyl-1H-pyrazolo[3,4-b]pyridine-4-carboxamide (**11o**).* Colorless solid, mp 204-206 °C. [Found: C, 59.75%; H, 4.83%; N, 10.65%. $C_{33}H_{31}ClBrN_5O_3$ requires C, 59.96%; H, 4.73%; N, 10.60 %]; ^1H NMR (300 MHz, DMSO-d_6, δ, ppm): 13.26 (s, 1H, NH),

8.10 (s, 1H, NH), 7.01-8.08 (m, 12H, ArH), 7.40 (s, 1H, CH), 6.29 (s, 1H, CH), 3.83 (s, 3H, $\underline{CH_3}O$), 2.58 (s, 3H, CH_3), 1.31 (s, 9H, $\underline{(CH_3)_3}C$); NMR ^{13}C (75 MHz, DMSO-d_6, δ, ppm): 168.5, 166.6, 166.5, 160.5, 160.3, 154.8, 154.3, 153.9, 138.4, 133.8, 132.8, 132.0, 131.1, 130.7, 130.6, 128.3, 128.1, 120.7, 114.2, 111.6, 111.0, 109.0, 63.0, 55.2, 50.5, 28.3, 13.3. LC/MS (ESI): 661 $[M+2H]^+$.

*N-(2-(tert-Butylamino)-1-(4-chlorophenyl)-2-oxoethyl)-N,6-bis-(4-methoxyphenyl)-3-methyl-1H-pyrazolo[3,4-b]pyridine-4-carboxamide (**11p**).* Colorless solid, mp 145-146 °C. [Found: C, 66.65%; H, 5.64%; N, 11.43%. $C_{34}H_{34}ClN_5O_4$ requires C, 66.71%; H, 5.60%; N, 11.44 %]; ^1H NMR (300 MHz, DMSO-d_6, δ, ppm): 13.18 (s, 1H, NH), 7.99 (s, 1H, NH), 6.44-7.91 (m, 12H, ArH), 7.34 (s, 1H, CH), 6.25 (s, 1H, CH), 3.81 (s, 3H, $\underline{CH_3}O$), 3.45 (s, 3H, $\underline{CH_3}O$), 2.56 (s, 3H, CH_3), 1.31 (s, 9H, $\underline{(CH_3)_3}C$); NMR ^{13}C (75 MHz, DMSO-d_6, δ, ppm): 168.6, 167.1, 160.4, 157.8, 154.2, 153.0, 140.5, 139.0, 134.1, 132.7, 132.5, 132.1, 131.5, 130.7, 128.3, 127.9, 114.2, 112.7, 111.0, 108.8, 63.1, 55.2, 54.9, 50.4, 28.3, 13.4. LC/MS (ESI): 611 $[M]^+$.

*N-(2-(tert-Butylamino)-1-(4-nitrophenyl)-2-oxoethyl)-6-(4-methoxyphenyl)-3-methyl-N-phenyl-1H-pyrazolo[3,4-b]pyridine-4-carboxamide (**11q**).* Yellowish solid, 128-130 °C. [Found: C, 66.84%; H, 5.39%; N, 14.20%. $C_{33}H_{32}N_6O_6$ requires C, 66.88%; H, 5.44%; N, 14.18%]; ^1H NMR (400 MHz, DMSO-d_6, δ, ppm): 13.16 (s, 1H, NH), 8.10 (s, 1H, NH), 6.83-8.05 (m, 15H, ArH+CH), 6.37 (s, 1H, CH), 3.77 (s, 3H, $\underline{CH_3}O$), 2.55 (s, 3H, CH_3), 1.27 (s, 9H, $\underline{(CH_3)_3}C$); NMR ^{13}C (100 MHz, DMSO-d_6, δ, ppm): 170.4, 169.5, 167.5, 163.1, 157.1, 155.6, 149.5, 145.2, 141.4, 141.1, 134.2, 133.9, 133.2, 130.9, 130.6, 130.4, 125.5, 116.8, 113.9, 66.2, 57.9, 53.3, 30.9, 15.9. LC/MS (ESI): 593 $[MH]^+$.

General procedure for the synthesis of *N*-(1-aryl-2-(*tert*-butylamino)-2-oxoethyl)-4-(4-methoxyphenyl)-3-methyl-*N*,1-diaryl-1*H*-pyrazolo[3,4-*b*]pyridine-6-carboxamides 12a-f.

The appropriate aniline **9** (0.5 mmol) and aromatic aldehyde **8** (0.5 mmol) were dissolved in DMF/CH_3OH 1:2 (2 mL) and the mixture stirred for 1 hour. Then, 4-(4-methoxyphenyl)-3-methyl-1-phenyl-1*H*-pyrazolo[3,4-*b*]pyridine-6-carboxylic acid (**7b**, 0.5 mmol) and *tert*-butylisocyanide (**10**, 0.5 mmol) were added consecutively and the reaction mixture stirred for 72 h at 70 °C in the closed vial until completion. Afterwards, the mixture was stirred for 1–2 hours before precipitation; the formed precipitate was filtered off and dried. In case of well-soluble substances the reaction mixture was poured onto ice, ultrasonicated for 15–30 min, and filtered. The collected precipitate was treated with 5 mL of EtOAc/hexane 1:15, ultrasonicated for additional 15–30 min, filtered again and dried.

*N-(2-(tert-Butylamino)-2-oxo-1-phenylethyl)-4-(4-methoxyphenyl)-3-methyl-N,1-diphenyl-1H-pyrazolo[3,4-b]pyridine-6-carboxamide (**12a**);* Colorless solid, 192-194 °C. [Found: C, 75.15%; H, 5.95%; N, 11.20%. $C_{39}H_{37}N_5O_3$ requires C, 75.10%; H, 5.98%; N, 11.23%]; ^{1}H NMR (400 MHz, DMSO-d_6, δ, ppm): 6.84-7.85 (m, 21H, ArH+CH+NH), 6.31 (s, 1H, CH), 3.81 (s, 3H, $\underline{CH_3}O$), 2.19 (s, 3H, CH_3), 1.21 (s, 9H, $\underline{(CH_3)_3}C$); NMR ^{13}C (100 MHz, DMSO-d_6, δ, ppm): 168.4, 167.6, 159.9, 153.5, 149.0, 145.7, 142.1, 138.5, 137.3, 135.6, 135.4, 130.4, 130.2, 129.9, 128.8, 128.3, 128.0, 127.7, 127.4, 125.7, 120.6, 117.7, 113.9, 113.5, 64.6, 55.2, 50.3, 28.3, 20.2, 15.2. LC/MS (ESI): 624 $[MH]^+$.

*N-(2-(tert-Butylamino)-2-oxo-1-phenylethyl)-4-(4-methoxyphenyl)-3-methyl-1-phenyl-N-(p-tolyl)-1H-pyrazolo[3,4-b]pyridine-6-carboxamide (**12b**);* Colorless solid, 175-177 °C. [Found: C, 75.36%; H, 6.16%; N, 10.99%. $C_{40}H_{39}N_5O_3$ requires C, 75.33%; H, 6.16%; N, 10.98%]; ^{1}H NMR (400 MHz, DMSO-d_6, δ, ppm): 6.74-7.82 (m, 20H, ArH+CH+NH), 6.27 (s, 1H, CH), 3.81 (s, 3H, $\underline{CH_3}O$), 2.19 (s, 3H, CH_3), 1.94 (s, 3H, CH_3), 1.21 (s, 9H, $\underline{(CH_3)_3}C$); NMR ^{13}C (100 MHz, DMSO-d_6, δ, ppm): 168.3, 167.5, 160.0, 153.3, 149.0, 145.7, 142.1, 139.9, 138.4, 135.3,

130.4, 130.4, 129.9, 128.8, 128.2, 127.7, 127.4, 126.5, 125.6, 120.5, 117.6, 113.8, 113.5, 64.6, 55.2, 50.3, 28.2, 15.1. LC/MS (ESI): 638 $[MH]^+$.

*N-(2-(tert-Butylamino)-1-(4-chlorophenyl)-2-oxoethyl)-4-(4-methoxyphenyl)-3-methyl-N,1-diphenyl-1H-pyrazolo[3,4-b]pyridine-6-carboxamide (**12c**);* Colorless solid, mp 167-168 °C. [Found: C, 71.19%; H, 5.43%; N, 10.85%. $C_{39}H_{36}ClN_5O_3$ requires C, 71.17%; H, 5.51%; N, 10.64%]; ^1H NMR (400 MHz, DMSO-d_6, δ, ppm): 6.88-7.78 (m, 20H, ArH+CH+NH), 6.26 (s, 1H, CH), 3.81 (s, 3H, $\underline{CH_3}O$), 2.19 (s, 3H, CH_3), 1.20 (s, 9H, $\underline{(CH_3)_3}C$); NMR ^{13}C (100 MHz, DMSO-d_6, δ, ppm): 168.0, 167.5, 160.0, 153.2, 148.9, 145.8, 142.1, 139.7, 138.4, 134.4, 132.2, 131.7, 130.5, 130.4, 128.9, 128.2, 127.8, 127.6, 126.7, 125.7, 120.6, 117.7, 113.9, 113.5, 63.9, 55.2, 50.4, 28.1, 15.2. LC/MS (ESI): 658 $[MH]^+$.

*N-(2-(tert-Butylamino)-1-(4-chlorophenyl)-2-oxoethyl)-4-(4-methoxyphenyl)-3-methyl-1-phenyl-N-(p-tolyl)-1H-pyrazolo[3,4-b]pyridine-6-carboxamide (**12d**);* Colorless solid, mp 182-183 °C. [Found: C, 71.38%; H, 5.80%; N, 10.40%. $C_{40}H_{38}ClN_5O_3$ requires C, 71.47%; H, 5.70%; N, 10.42%]; ^1H NMR (400 MHz, DMSO-d_6, δ, ppm): 6.8-7.86 (m, 19H, ArH+CH+NH), 6.25 (s, 1H, CH), 3.81 (s, 3H, $\underline{CH_3}O$), 2.18 (s, 3H, CH_3), 1.96 (s, 3H, CH_3), 1.20 (s, 9H, $\underline{(CH_3)_3}C$); NMR ^{13}C (100 MHz, DMSO-d_6, δ, ppm): 168.0, 167.5, 160.0, 153.3, 148.9, 145.7, 142.1, 138.4, 137.1, 135.9, 134.4, 132.2, 131.7, 130.4, 130.2, 128.8, 128.1, 127.7, 125.7, 120.7, 117.65, 113.9, 113.5, 109. 5, 63.9, 55.2, 50.4, 28.3, 20.3, 15.2. LC/MS (ESI): 672 $[MH]^+$.

*N-(2-(tert-Butylamino)-1-(4-methoxyphenyl)-2-oxoethyl)-4-(4-methoxyphenyl)-3-methyl-N,1-diphenyl-1H-pyrazolo[3,4-b]pyridine-6-carboxamide (**12e**);* Colorless solid, mp 171-172 °C. [Found: C, 73.47%; H, 6.04%; N, 10.68%. $C_{40}H_{39}N_5O_4$ requires C, 73.49%; H, 6.01%; N, 10.71%]; ^1H NMR (400 MHz, DMSO-d_6, δ, ppm): 6.67-7.77 (m, 20H, ArH+CH+NH) , 6.21 (s, 1H, CH), 3.81 (s, 3H, $\underline{CH_3}O$), 3.61 (s, 3H, $\underline{CH_3}O$), 2.19 (s, 3H, CH_3), 1.21 (s, 9H, $\underline{(CH_3)_3}C$); NMR ^{13}C (100 MHz, DMSO-d_6, δ, ppm): 168.7, 167.5, 160.0, 158.5, 153.4, 149.0, 145.7,

142.1, 139.9, 138.4, 131.2, 130.6, 130.4, 128.9, 128.6, 128.2, 127.4, 127.1, 126.5, 125.6, 120.6, 117.6, 113.9, 113.4, 113.1, 64.0, 55.2, 54.9, 50.3, 15.2. LC/MS (ESI): 654 $[MH]^+$.

*N-(2-(tert-Butylamino)-1-(4-methoxyphenyl)-2-oxoethyl)-4-(4-methoxyphenyl)-3-methyl-1-phenyl-N-(p-tolyl)-1H-pyrazolo[3,4-b]pyridine-6-carboxamide (**12f**);* Colorless solid, mp 149-150 °C. [Found: C, 73.56%; H, 6.23%; N, 10.59%. $C_{41}H_{41}N_5O_4$ requires C, 73.74%; H, 6.19%; N, 10.49%]; ^{1}H NMR (400 MHz, DMSO-d_6, δ, ppm): 6.72-7.81 (m, 19H, ArH+CH+NH), 6.23 (s, 1H, CH), 3.84 (s, 3H, $\underline{CH_3}O$), 3.65 (s, 3H, $\underline{CH_3}O$), 2.22 (s, 3H, CH_3), 1.99 (s, 3H, CH_3) , 1.25 (s, 9H, $\underline{(CH_3)_3}C$); NMR ^{13}C (100 MHz, DMSO-d_6, δ, ppm): 168.8, 167.6, 160.0, 158.5, 153.6, 149.1, 145.7, 142.2, 138.5, 137.4, 135.7, 131.3, 130.4, 130.4, 128.9, 128.1, 127.3, 125.8, 120.7, 117.7, 113.9, 113.2, 109.5, 64.1, 55.3, 54.9, 50.3, 28.4, 20.3, 15.24. LC/MS (ESI): 668 $[MH]^+$.

3. X-ray analysis

The crystals of **11n** ($C_{34}H_{34}N_5O_3Cl$) are monoclinic. At 293 K, a = 28.239(2), b = 10.9641(6), c = 21.090(1) Å, β = 94.270(5)°, V = 6511.7(7) Å^3, M_r = 596.11, Z = 8, space group $C2/c$, d_{calc}= 1.216 g/cm^3, μ(MoKα) = 0.158 mm^{-1}, F(000) = 2512. Parameters of the primitive cell and intensities of 21036 reflections (5695 independent, R_{int} = 0.130) were measured on an «X-callibur» diffractometer (graphite monochromated MoKα radiation, CCD-detector, ω scanning, $2\Theta_{max}$ = 50°).

The structures were solved by direct method using SHELXTL package [4]. Positions of hydrogen atoms were located from electron density difference maps and refined using riding model with U_{iso} = nU_{eq} (n = 1.5 for methyl groups and water molecule and 1.2 for other hydrogen atoms). Full-matrix least-squares refinement against F^2 in anisotropic approximation for non-hydrogen atoms was converged to wR_2 = 0.192 for 5695 reflections (R_1 = 0.089 for 2577 reflections with F>4σ(F), S = 0.959). The final atomic coordinates, and crystallographic data for molecule **11n** have been deposited to the Cambridge Crystallographic Data Centre, 12

Union Road, CB2 1EZ, UK (fax: +44-1223-336033; e-mail: deposit@ccdc.cam.ac.uk) and are available on request quoting the deposition numbers CCDC 1898425).

4. Antibacterial activity

The study of antibacterial activity of the compounds was performed in vitro by the microdilution method. In order to prepare microdilution trays twofold serial dilution of the solutions of antimicrobial agents in a liquid culture medium (Mueller-Hinton broth (MHB)), ("HiMedia Laboratories", India) was made. Disposable polystyrene 96-well cell culture plates for immunological studies (DELTALAB, Spain) and Proline Plus 8-channel mechanical pipettes were used during the experiment. The analyses were carried out according to recommendations of CLSI (Clinical and Laboratory Standards Institute, USA) [5-8] and analogous as described in [9].

In the beginning the solutions of the test compounds at concentrations of 500, 250, 125, 62.5, 31.25 and 15.6 mg/L were added to the wells of the plate using the twofold serial dilution method (the total volume of 200 μL was constant). Collectable reference strains of bacteria were used as test cultures: *Bacillus subtilis* (strain 1211), *Staphylococcus aureus* (strain 2231) - gram-positive cultures; *Escherichia coli* (strain 1257) and *Pseudomonas aeruginosa* (strain 1111) - gram-negative cultures. For the preparation of an inoculum from the isolated colonies of microorganisms being in the phase of exponential growth a suspension in saline was made according to McFarland standard (0.5). The suspension was diluted with MHB to a concentration of 10^5 CFU/mL (colony-forming unit in 1 mL) and a 100 μL aliquort was added to the walls already containing 100 μL of solutions of the compounds under study. Wells containing only the suspensions diluted with MHB without adding any substance served as the negative control. Sterility of the medium was controlled using the dedicated wells in microtiter

plate containing neither the solution of the substance nor microbial suspension. Inoculated plates were incubated at 37 °C for 18 ± 2 hours.

The presence or absence of culture growth was assessed visually and spectrophotometrically by comparison with the growth of a microorganism in the presence of the compound studied with the growth of the microorganism without any compound. The minimum inhibitiory concentration (MIC) was defined as the lowest concentration of the antimicrobial agent (mg/L) able to inhibit any visible growth of the culture. The minimum bactericidal concentration (MBC) was determined by sowing out the content of those tubes with the absence of any signs of growth on peptone-meat extract agar in Petri dishes. The absorbance of the medium during the growth of the cultures was measured with the help of microplate absorbance reader Sunrise RC (Tecan Austria GmbH, Switzerland) at 492 nm (reference wavelength 620 nm). Nitroxoline, a quinolone-based drug, was used as a comparison standard.

5. References

1. Chebanov, V. A.; Sakhno, Y. I.; Desenko, S. M.; Chernenko, V. N.; Musatov, V. I.; Shishkina, S. V.; Shishkin O. V. and Kappe, C. O. *Tetrahedron* **2007**, *63*, 1229–1242.
2. Annan, N.; Paris R. and Jordan, F. *J. Am. Chem. Soc.* **1989**, *111*, 8895–8901.
3. Meng, Q.; Zhu L. and Zhang, Z. *J. Org. Chem.* **2008**, *73*, 7209–7212.
4. Sheldrick, G. M. *Acta Crystallogr. Sect. A Found. Crystallogr.* **2008**, *64*, 112–122.
5. CLSI. *Methods for Dilution Antimicrobial Susceptibility Tests for Bacteria That Grow Aerobically; Approved Standard—Tenth Edition.* CLSI document M07-A10. Wayne, PA: Clinical and Laboratory Standards Institute; **2015**.
6. ISO 20776-1:2006. *Clinical laboratory testing and in vitro diagnostic test systems - Susceptibility testing of infectious agents and evaluation of performance of antimicrobial susceptibility test devices - Part 1: Reference method for testing the in vitro activity of antimicrobial agents against rapidly growing aerobic bacteria involved in infectious diseases*; **2006**. https://www.iso.org/standard/41630.html
7. Eucast Definitive Document. *Methods for the determination of susceptibility of bacteria to antimicrobial agents. Terminology. Clin. Microbiol. Infect.* **1998**, *4*(5), 291-296. http://www.sciencedirect.com/science/article/pii/S1198743X14650473
8. Cornaglia, G.; Hryniewicz, W.; Jarlier, V.; Kahlmeter, G.; Mittermayer, H.; Stratchounski, L.; Baquero, F. *Clin. Microbiol. Infect.* **2004**, *10*, 349–383.
9. Murlykina, M. V.; Kornet, M. N.; Desenko, S. M.; Shishkina, S. V.; Shishkin, O. V.; Brazhko, A. A.; Musatov, V. I.; Van der Eycken, E. V.; Chebanov, V. A. *Beilstein J. Org. Chem.* **2017**, *13*, 1050–1063.
