# Supplementary material for: Doebner-type pyrazolopyridine carboxylic acids in an Ugi four-component reaction
Source: Beilstein J Org Chem. 2019 Jun 12;15:1281–8. doi: 10.3762/bjoc.15.126 (PMC6604699; doi:10.3762/bjoc.15.126)
Supplement: File 2 — NMR spectra. [file Beilstein_J_Org_Chem-15-1281-s002.pdf]

BEILSTEIN JOURNAL OF ORGANIC CHEMISTRY

Supporting Information

for

Doebner-type pyrazolopyridine carboxylic acids in an Ugi four-component reaction

Maryna V. Murlykina, Oleksandr V. Kolomiets, Maryna M. Kornet, Yana I. Sakhno, Sergey M. Desenko, Victoriya V. Dyakonenko, Svetlana V. Shishkina, Oleksandr A. Brazhko, Vladimir I. Musatov, Alexander V. Tsygankov, Erik V. Van der Eycken and Valentyn A. Chebanov

Beilstein J. Org. Chem. **2019,** *15,* 1281–1288. doi:10.3762/bjoc.15.126

NMR spectra

License and Terms: This is a supporting information file under the terms of the Creative Commons Attribution License (http://creativecommons.org/licenses/by/4.0). Please note that the reuse, redistribution and reproduction in particular requires that the authors and source are credited.
The license is subject to the *Beilstein Journal of Organic Chemistry* terms and conditions: (https://www.beilstein-journals.org/bjoc)

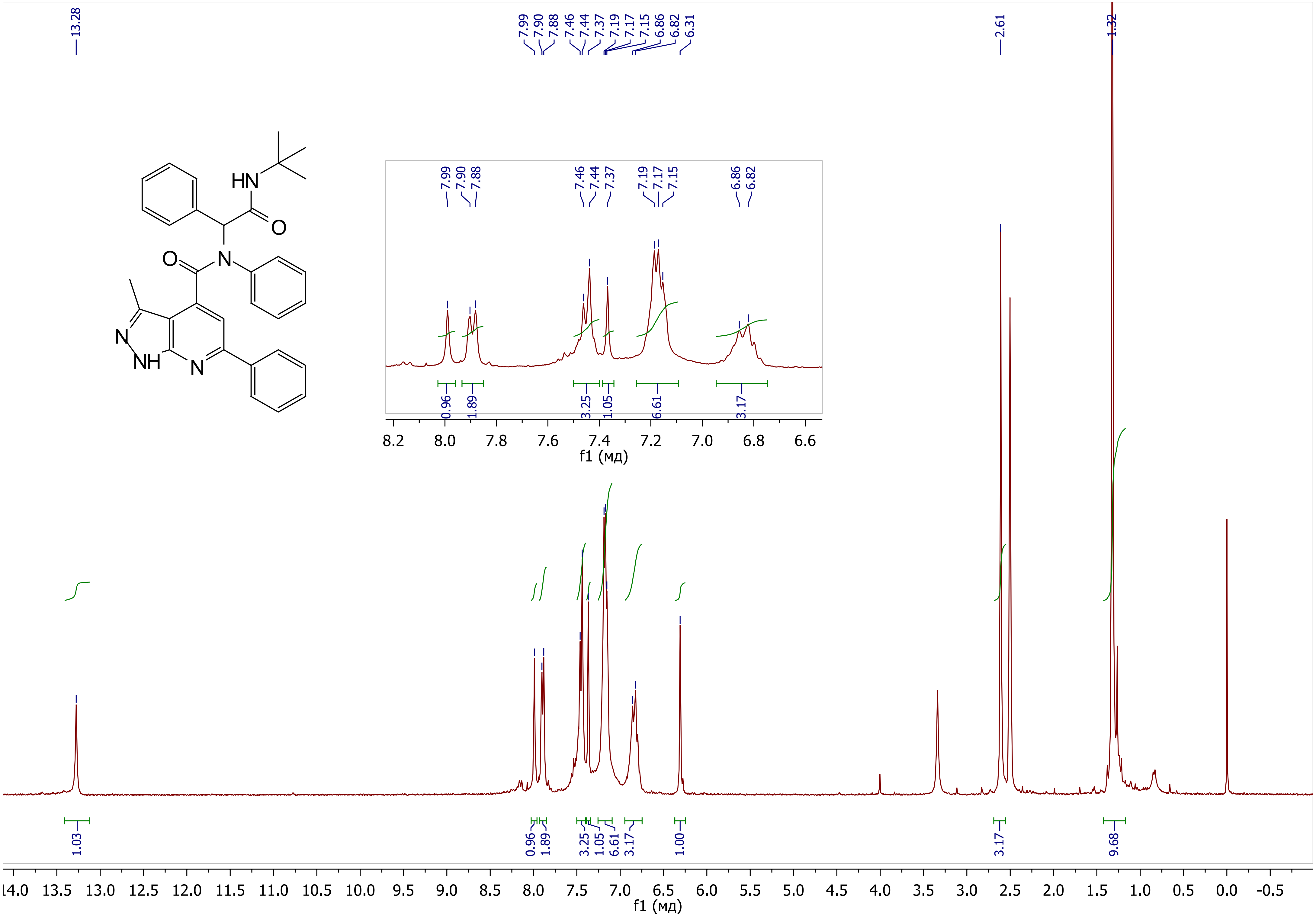
13.28
7.99
7.90
7.88
7.46
7.44
7.37
7.19
7.17
7.15
6.86
6.82
6.31
2.61
1.32
HN
O
O
N
N
NH
N
0.96
1.89
3.25
1.05
6.61
3.17
8.2 8.0 7.8 7.6 7.4 7.2 7.0 6.8 6.6
f1 (мд)
1.03
1.00
3.17
9.68
14.0 13.5 13.0 12.5 12.0 11.5 11.0 10.5 10.0 9.5 9.0 8.5 8.0 7.5 7.0 6.5 6.0 5.5 5.0 4.5 4.0 3.5 3.0 2.5 2.0 1.5 1.0 0.5 0.0 -0.5
f1 (мд)

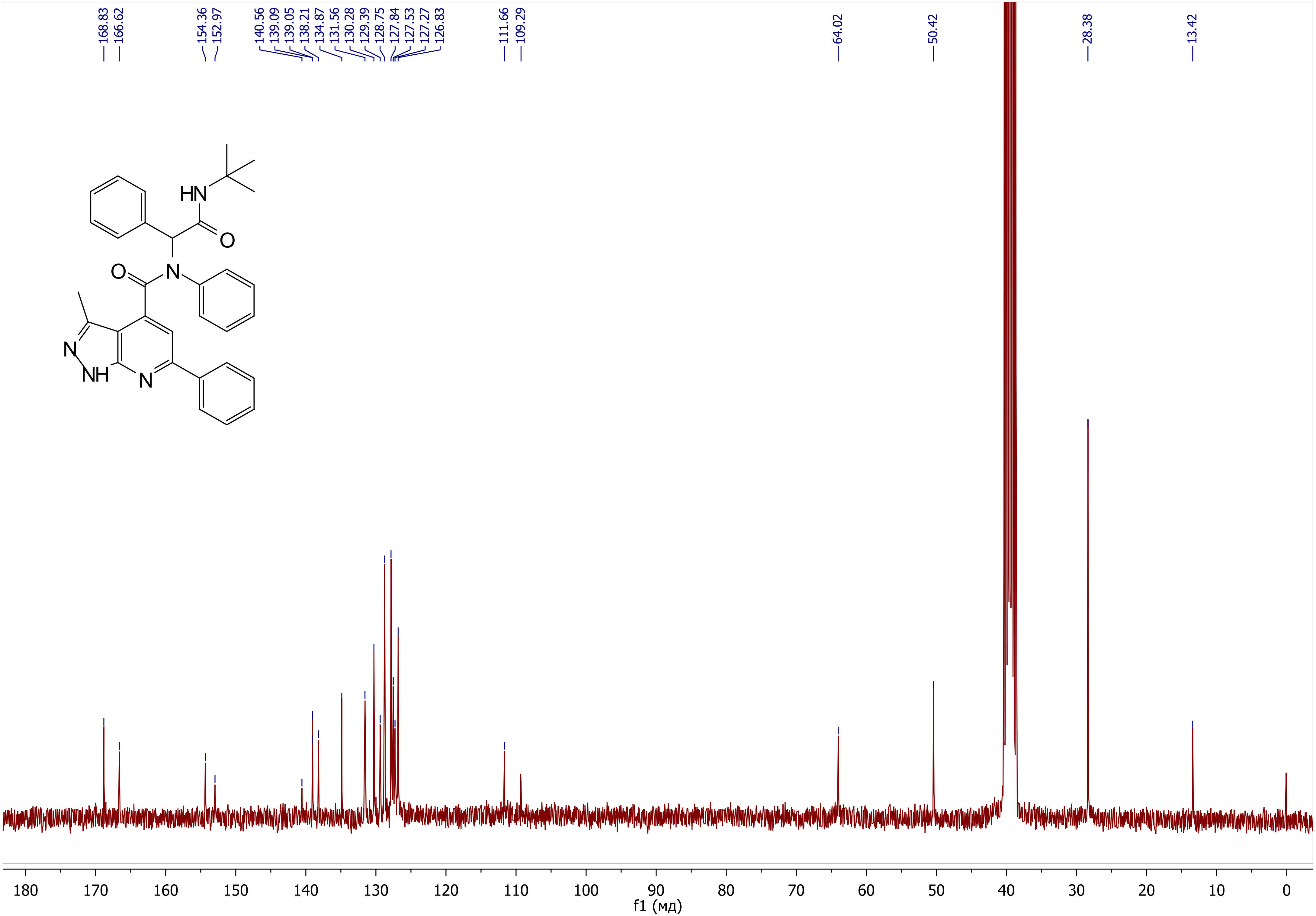
168.83
166.62
154.36
152.97
140.56
139.09
139.05
138.21
134.87
131.56
130.28
129.39
128.75
127.84
127.53
127.27
126.83
111.66
109.29
64.02
50.42
28.38
13.42
HN
O
O
N
N
NH
N
180 170 160 150 140 130 120 110 100 90 80 70 60 50 40 30 20 10 0
f1 (мд)

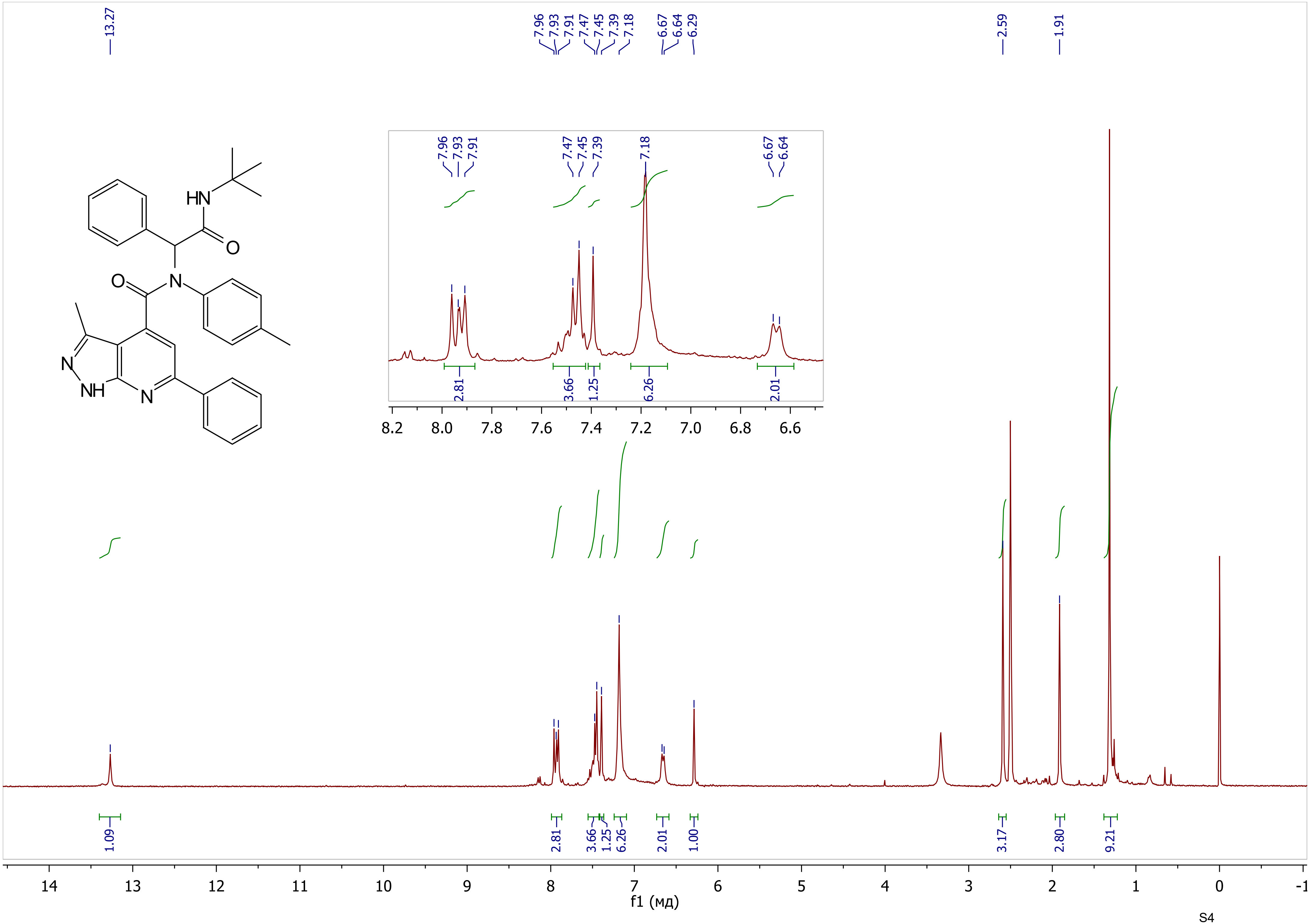
13.27
7.96
7.93
7.91
7.47
7.45
7.39
7.18
6.67
6.64
6.29
2.59
1.91
HN
O
N
O
N
NH
N
1.09
2.81
3.66
1.25
6.26
2.01
1.00
3.17
2.80
9.21
f1 (мд)

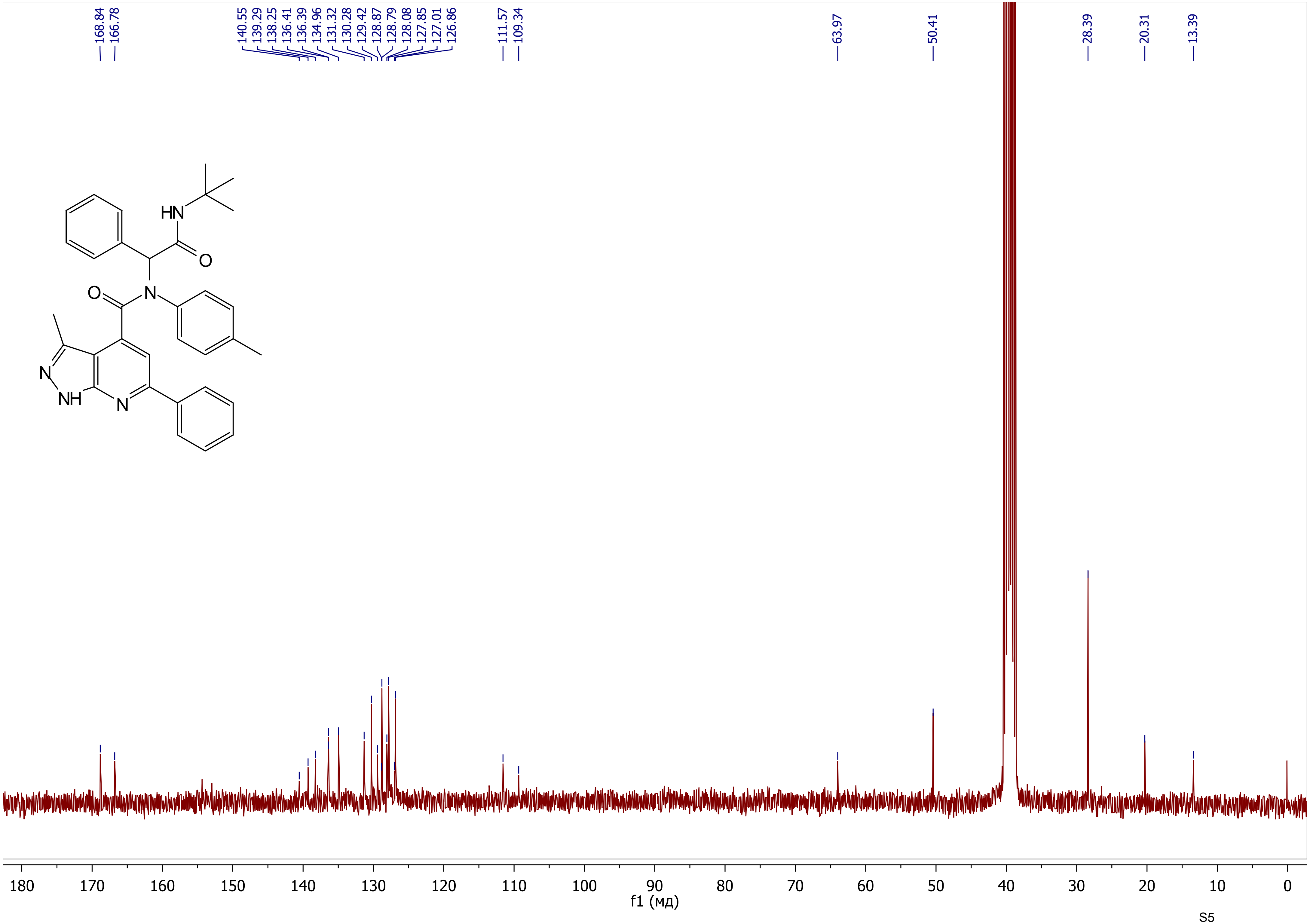
168.84
166.78
140.55
139.29
138.25
136.41
136.39
134.96
131.32
130.28
129.42
128.87
128.79
128.08
127.85
127.01
126.86
111.57
109.34
63.97
50.41
28.39
20.31
13.39
HN
O
O
N
N
NH
N
180 170 160 150 140 130 120 110 100 90 80 70 60 50 40 30 20 10 0
f1 (мд)

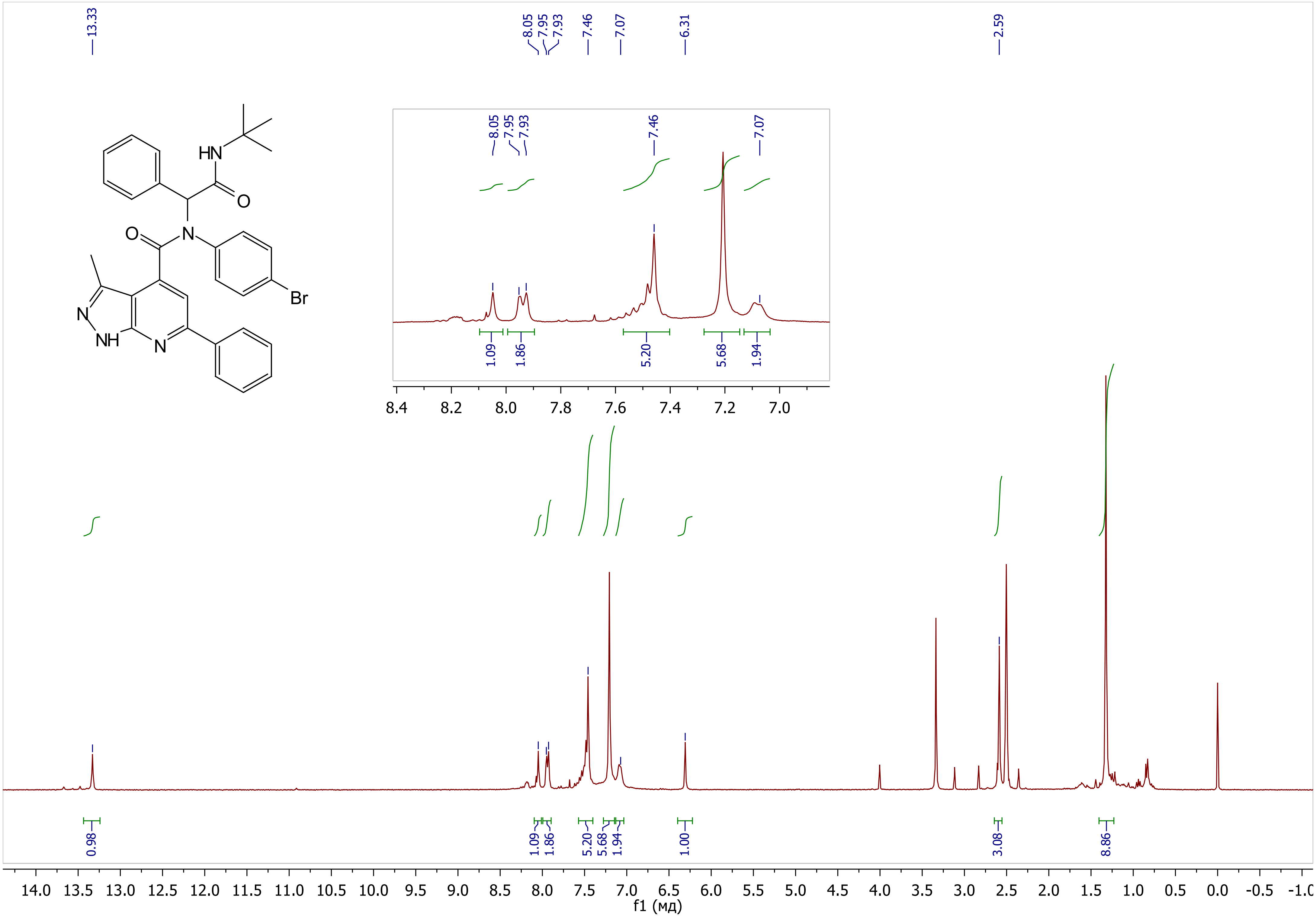
13.33
8.05
7.95
7.93
7.46
7.07
6.31
2.59
HN
O
N
Br
N
NH
N
1.09
1.86
5.20
5.68
1.94
8.4 8.2 8.0 7.8 7.6 7.4 7.2 7.0
0.98
1.00
3.08
8.86
14.0 13.5 13.0 12.5 12.0 11.5 11.0 10.5 10.0 9.5 9.0 8.5 8.0 7.5 7.0 6.5 6.0 5.5 5.0 4.5 4.0 3.5 3.0 2.5 2.0 1.5 1.0 0.5 0.0 -0.5
f1 (мд)

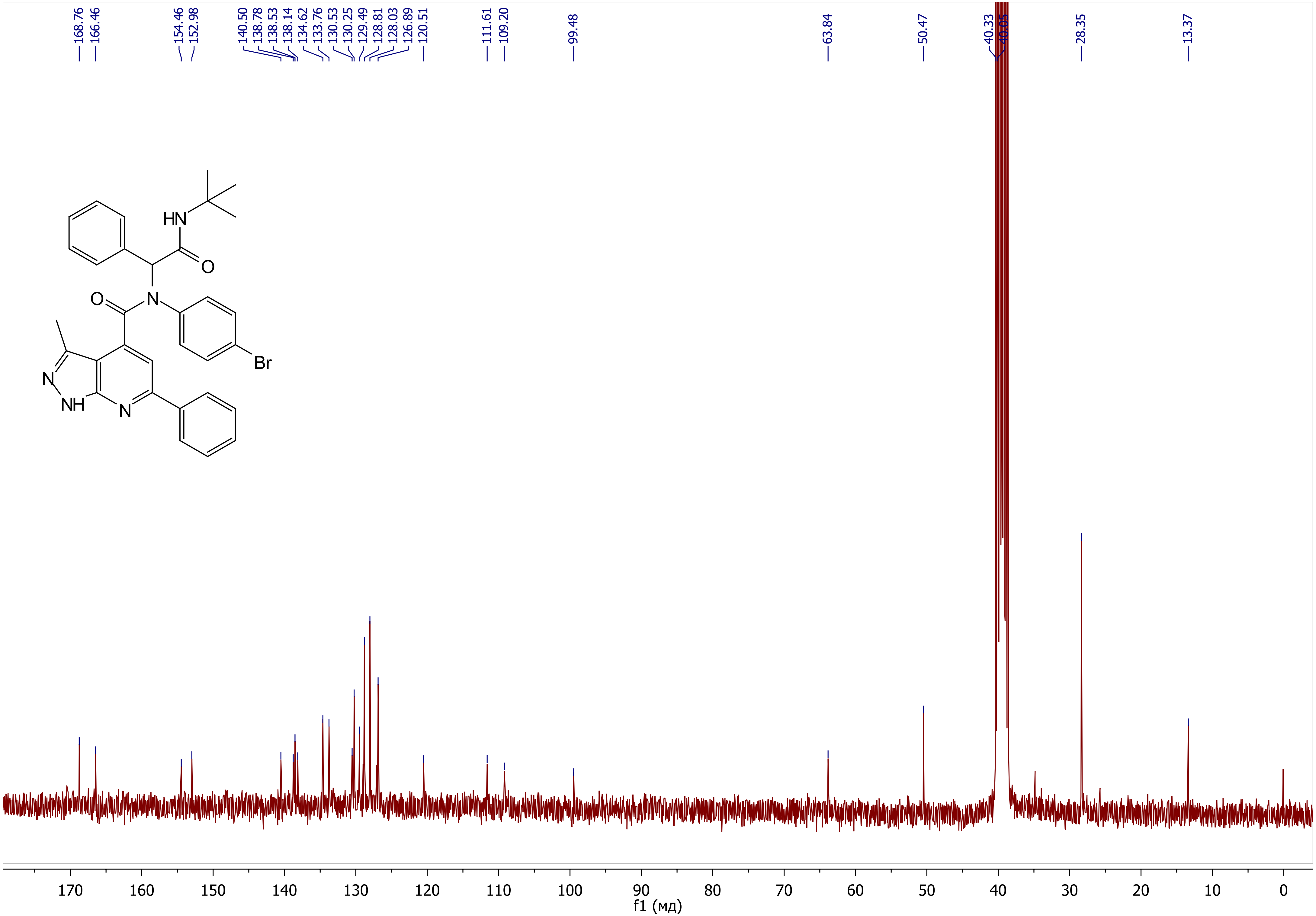
168.76
166.46
154.46
152.98
140.50
138.78
138.53
138.14
134.62
133.76
130.53
130.25
129.49
128.81
128.03
126.89
120.51
111.61
109.20
99.48
63.84
50.47
40.33
40.05
28.35
13.37
HN
O
O
N
N
NH
N
Br
170
160
150
140
130
120
110
100
90
80
70
60
50
40
30
20
10
0
f1 (мд)

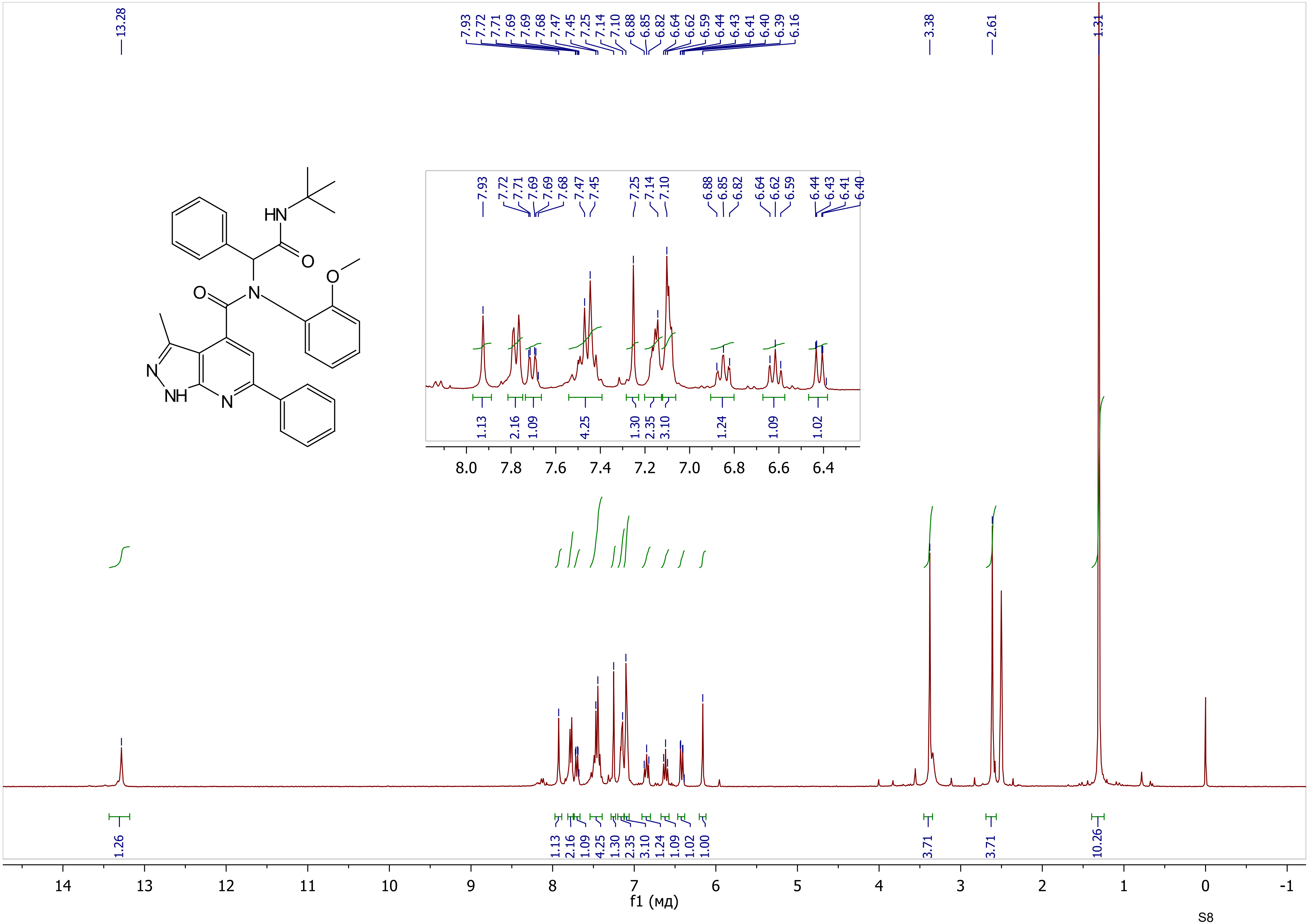
13.28
7.93 7.72 7.71 7.69 7.69 7.68 7.47 7.45 7.25 7.14 7.10 6.88 6.85 6.82 6.64 6.62 6.59 6.44 6.43 6.41 6.40 6.39 6.16
3.38
2.61
1.31
HN
O
O
N
O
N
NH
N
1.13 2.16 1.09 4.25 1.30 2.35 3.10 1.24 1.09 1.02
8.0 7.8 7.6 7.4 7.2 7.0 6.8 6.6 6.4
1.26
1.13 2.16 1.09 4.25 1.30 2.35 3.10 1.24 1.09 1.02 1.00
3.71
3.71
10.26
14 13 12 11 10 9 8 7 6 5 4 3 2 1 0 -1
f1 (мд)

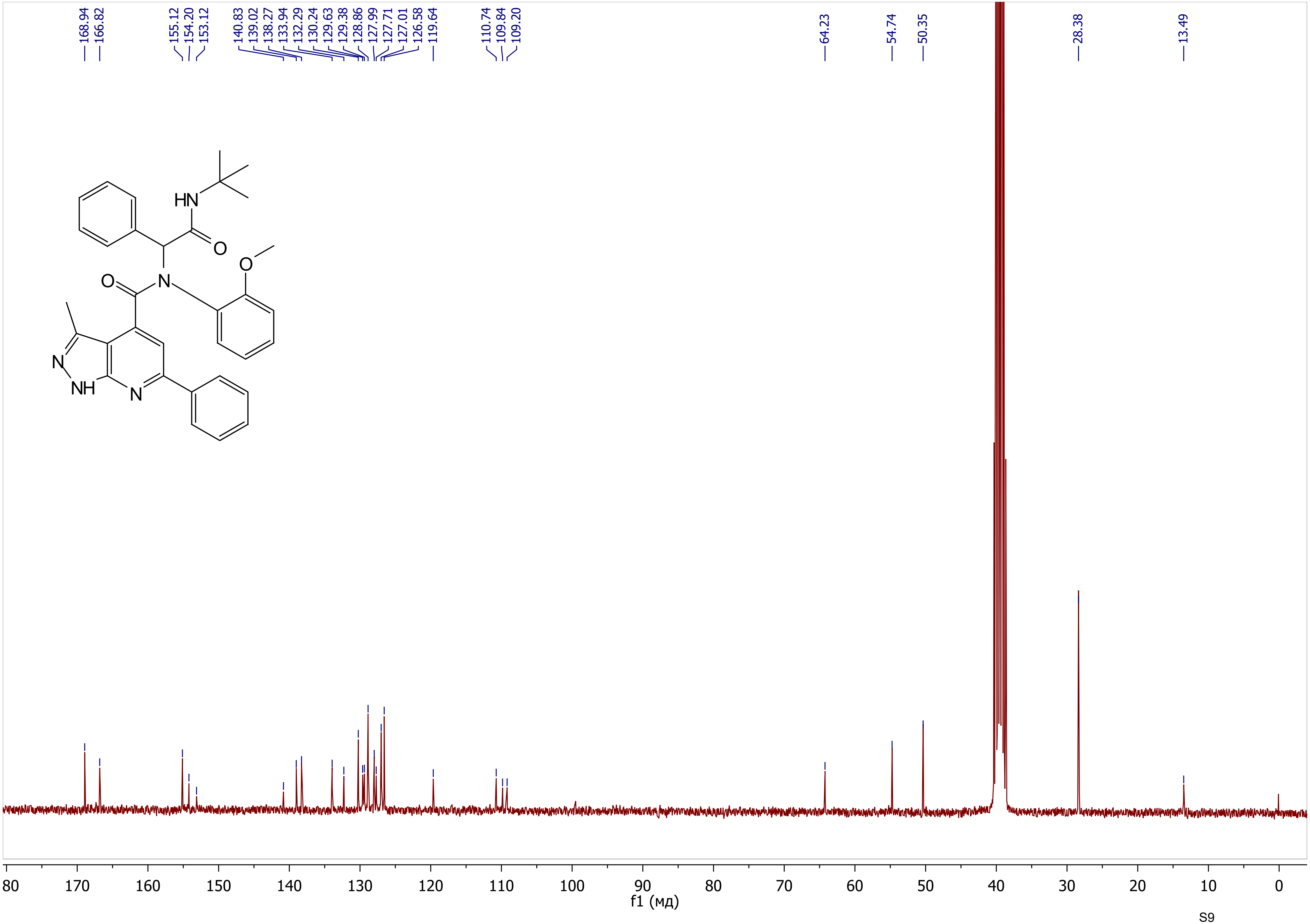
168.94
166.82
155.12
154.20
153.12
140.83
139.02
138.27
133.94
132.29
130.24
129.63
129.38
128.86
127.99
127.71
127.01
126.58
119.64
110.74
109.84
109.20
64.23
54.74
50.35
28.38
13.49
HN
O
O
N
O
N
NH
N
180
170
160
150
140
130
120
110
100
90
80
70
60
50
40
30
20
10
0
f1 (мд)

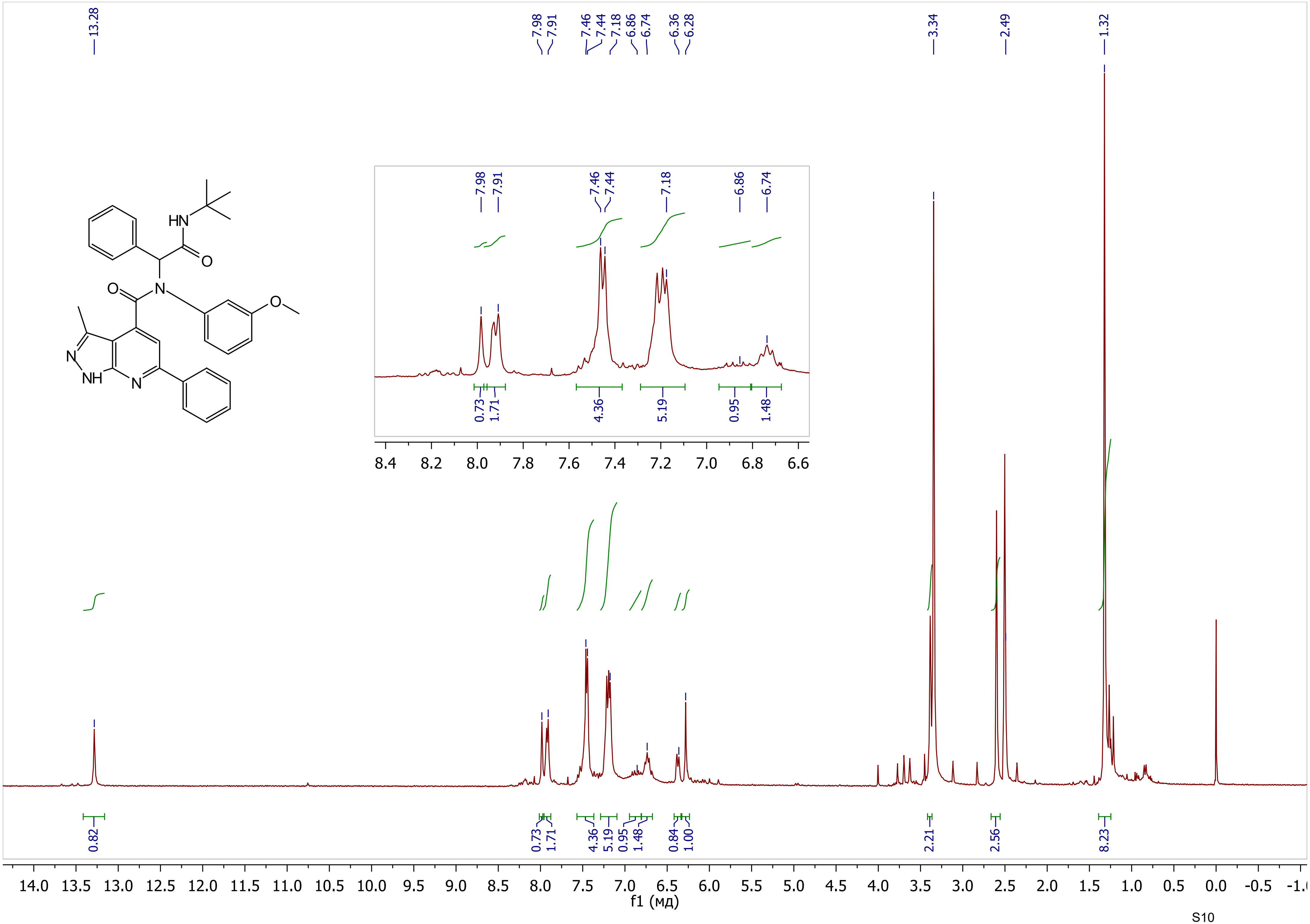
13.28
7.98
7.91
7.46
7.44
7.18
6.86
6.74
6.36
6.28
3.34
2.49
1.32
0.82
0.73
1.71
4.36
5.19
0.95
1.48
0.84
1.00
2.21
2.56
8.23
HN
O
O
N
N
NH
N
O
8.4 8.2 8.0 7.8 7.6 7.4 7.2 7.0 6.8 6.6
14.0 13.5 13.0 12.5 12.0 11.5 11.0 10.5 10.0 9.5 9.0 8.5 8.0 7.5 7.0 6.5 6.0 5.5 5.0 4.5 4.0 3.5 3.0 2.5 2.0 1.5 1.0 0.5 0.0 -0.5
f1 (мд)

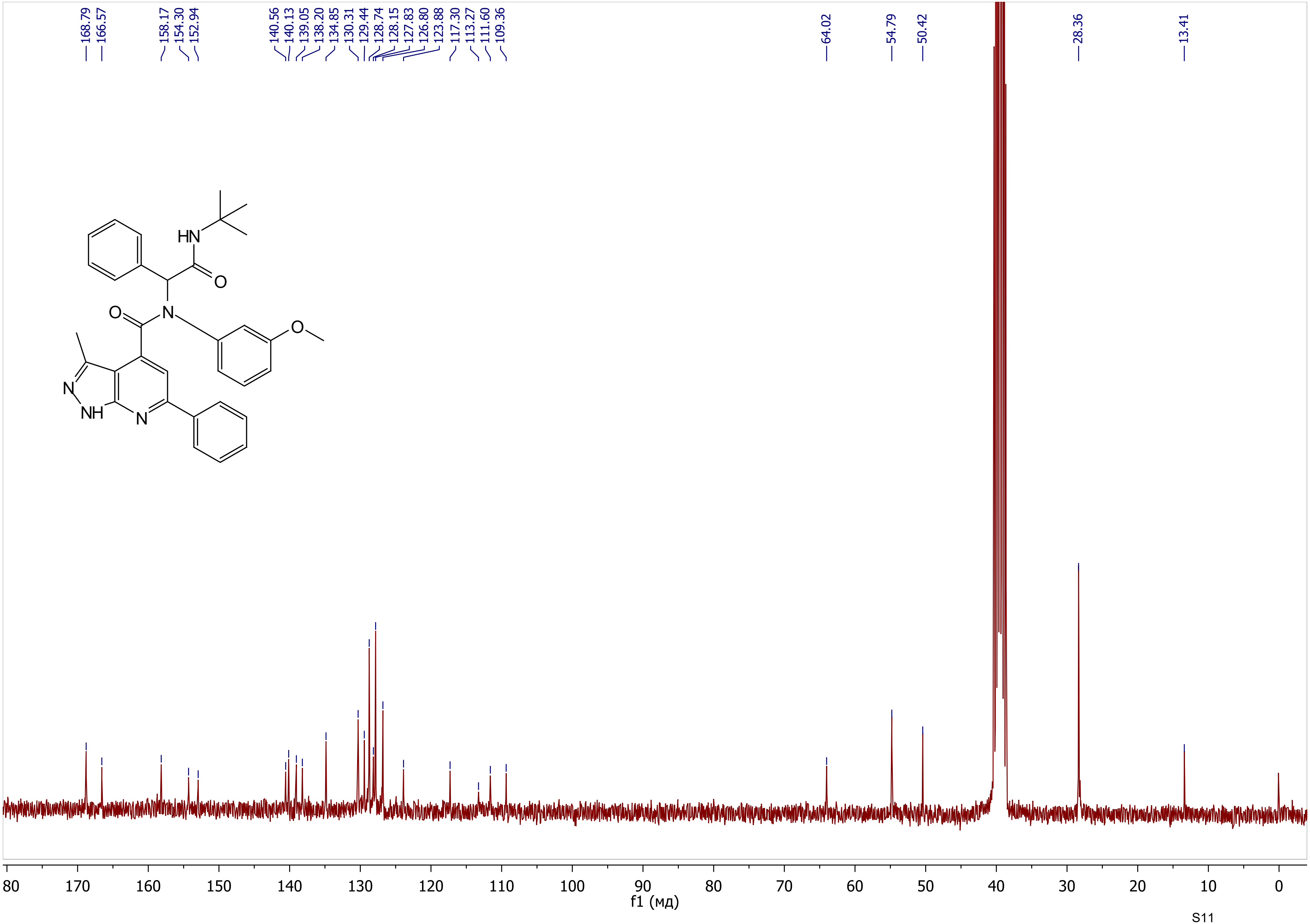
168.79
166.57
158.17
154.30
152.94
140.56
140.13
139.05
138.20
134.85
130.31
129.44
128.74
128.15
127.83
126.80
123.88
117.30
113.27
111.60
109.36
64.02
54.79
50.42
28.36
13.41
HN
O
O
N
N
NH
N
O
80 170 160 150 140 130 120 110 100 90 80 70 60 50 40 30 20 10 0
f1 (мд)

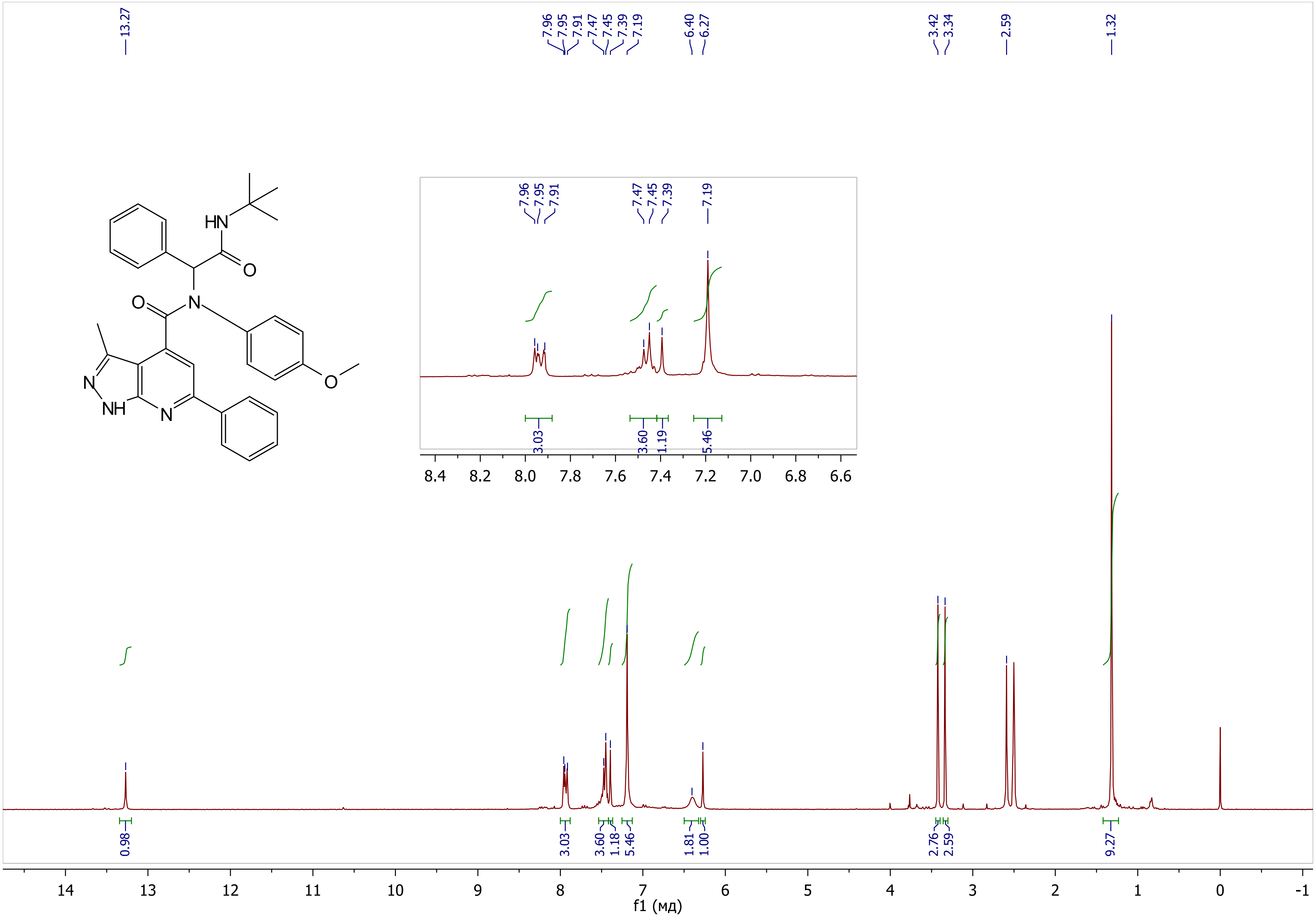
13.27
7.96
7.95
7.91
7.47
7.45
7.39
7.19
6.40
6.27
3.42
3.34
2.59
1.32
HN
O
N
N
NH
N
O
3.03
3.60
1.19
5.46
8.4 8.2 8.0 7.8 7.6 7.4 7.2 7.0 6.8 6.6
0.98
3.03
3.60
1.18
5.46
1.81
1.00
2.76
2.59
9.27
14 13 12 11 10 9 8 7 6 5 4 3 2 1 0 -1
f1 (мд)

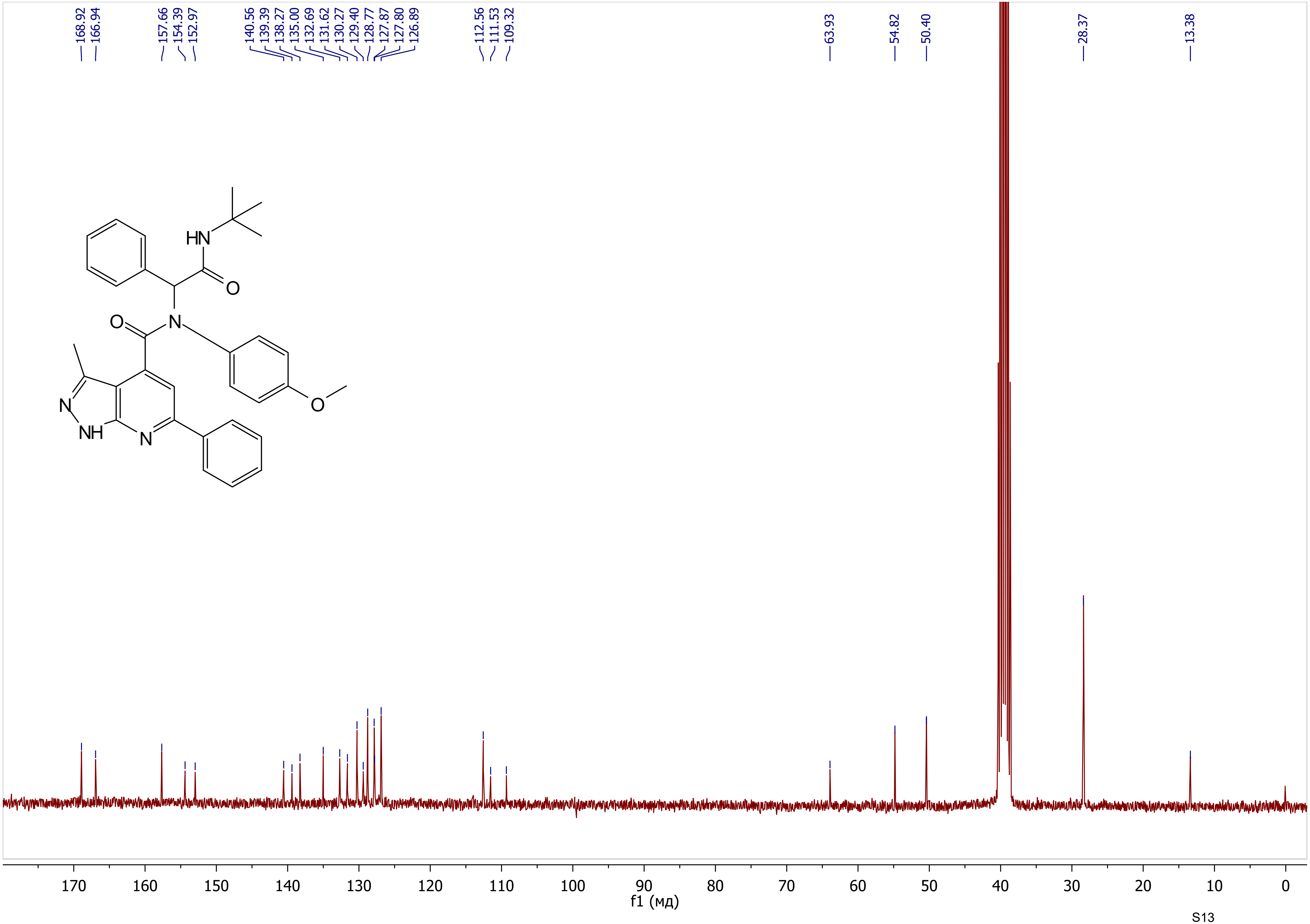
168.92
166.94
157.66
154.39
152.97
140.56
139.39
138.27
135.00
132.69
131.62
130.27
129.40
128.77
127.87
127.80
126.89
112.56
111.53
109.32
63.93
54.82
50.40
28.37
13.38
HN
O
O
N
N
NH
N
O
170
160
150
140
130
120
110
100
90
80
70
60
50
40
30
20
10
0
f1 (мд)

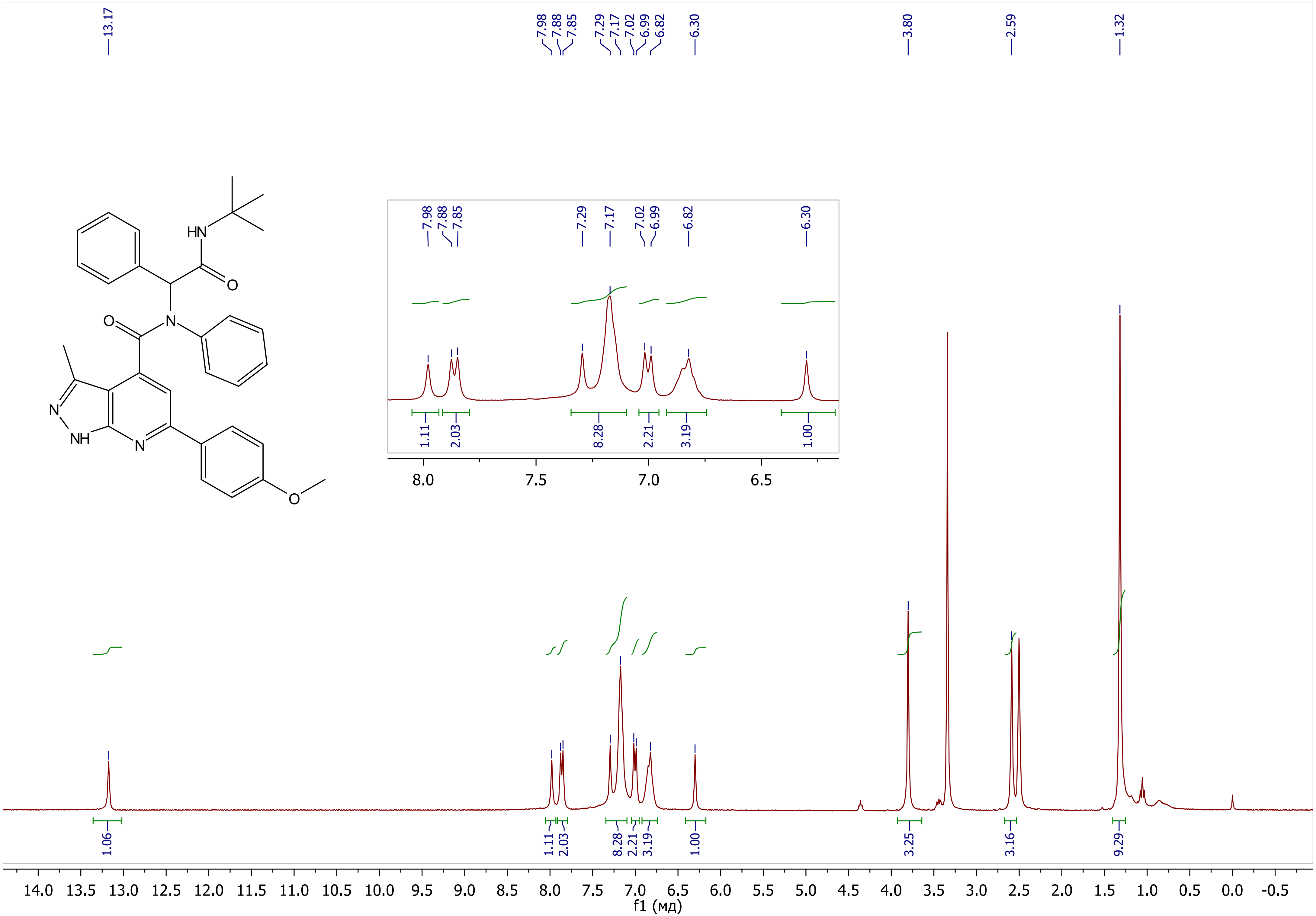
13.17
7.98
7.88
7.85
7.29
7.17
7.02
6.99
6.82
6.30
3.80
2.59
1.32
1.06
1.11
2.03
8.28
2.21
3.19
1.00
3.25
3.16
9.29
HN
O
N
O
N
NH
N
O
8.0
7.5
7.0
6.5
14.0 13.5 13.0 12.5 12.0 11.5 11.0 10.5 10.0 9.5 9.0 8.5 8.0 7.5 7.0 6.5 6.0 5.5 5.0 4.5 4.0 3.5 3.0 2.5 2.0 1.5 1.0 0.5 0.0 -0.5
f1 (мд)

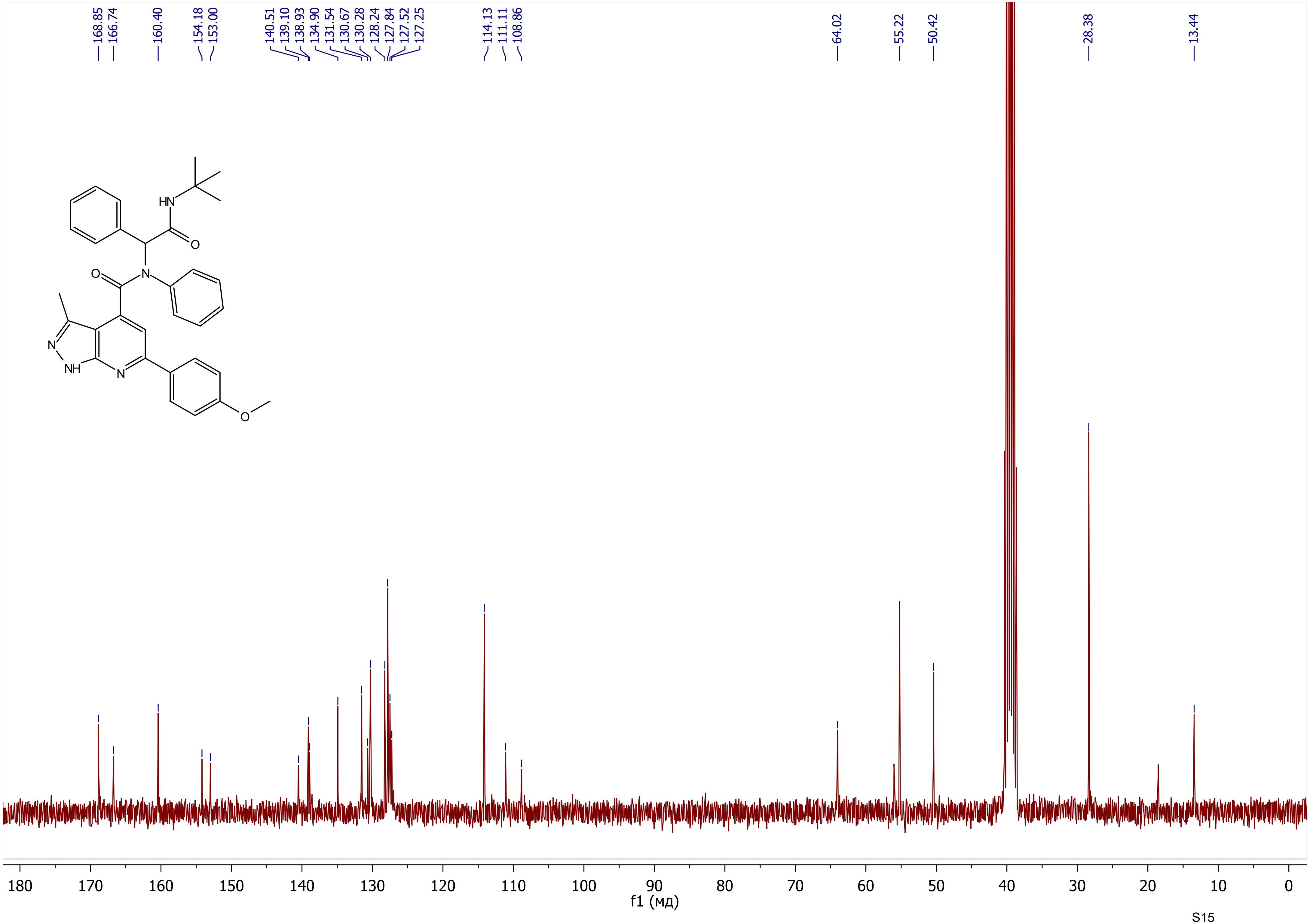

168.85
166.74
160.40
154.18
153.00
140.51
139.10
138.93
134.90
131.54
130.67
130.28
128.24
127.84
127.52
127.25
114.13
111.11
108.86
64.02
55.22
50.42
28.38
13.44
HN
O
O
N
N
NH
N
O
180
170
160
150
140
130
120
110
100
90
80
70
60
50
40
30
20
10
0
f1 (мд)

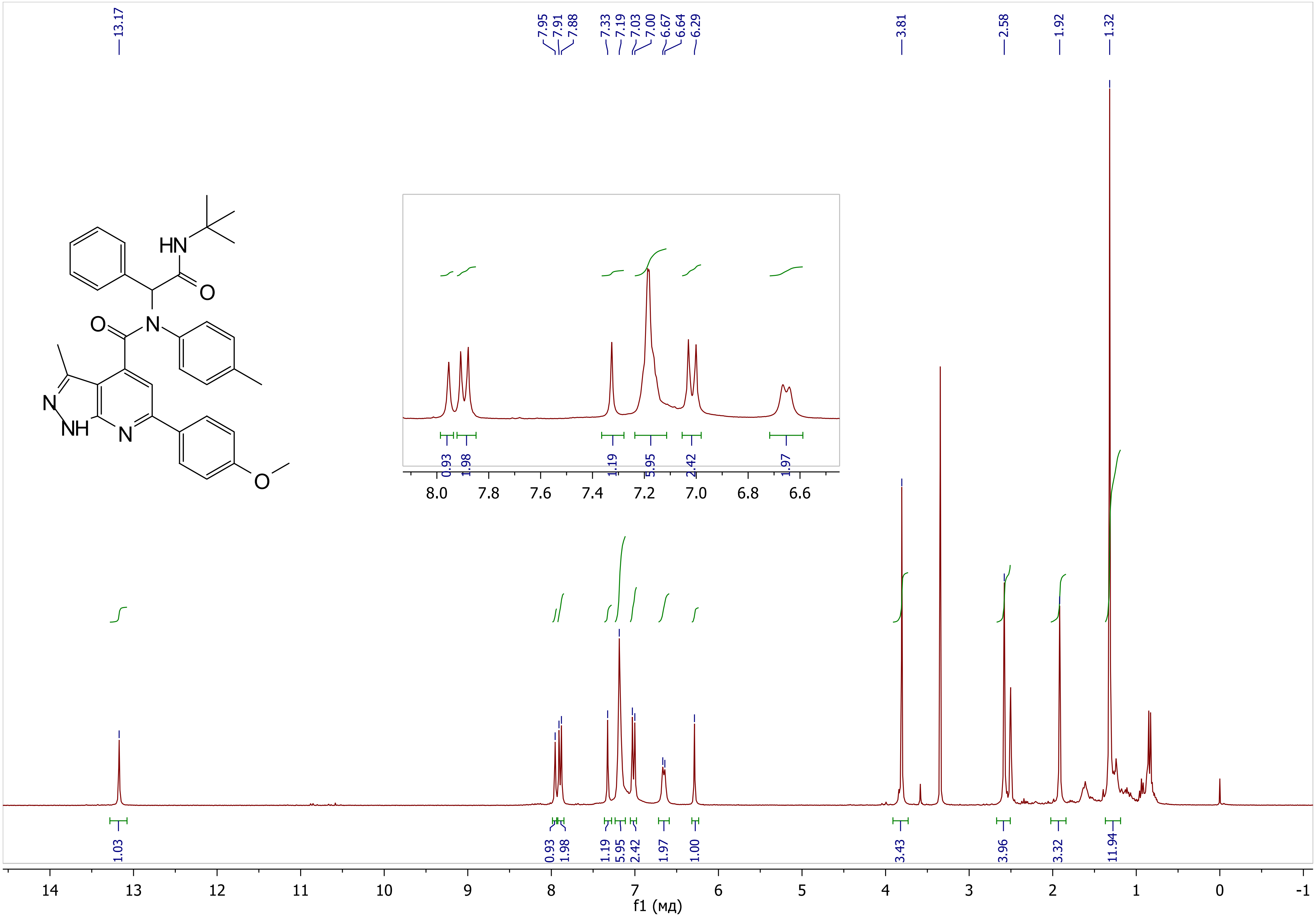
13.17
7.95
7.91
7.88
7.33
7.19
7.03
7.00
6.67
6.64
6.29
3.81
2.58
1.92
1.32
HN
O
O
N
N
NH
N
O
0.93
1.98
1.19
5.95
2.42
1.97
8.0 7.8 7.6 7.4 7.2 7.0 6.8 6.6
1.03
0.93
1.98
1.19
5.95
2.42
1.97
1.00
3.43
3.96
3.32
11.94
14 13 12 11 10 9 8 7 6 5 4 3 2 1 0 -1
f1 (мд)

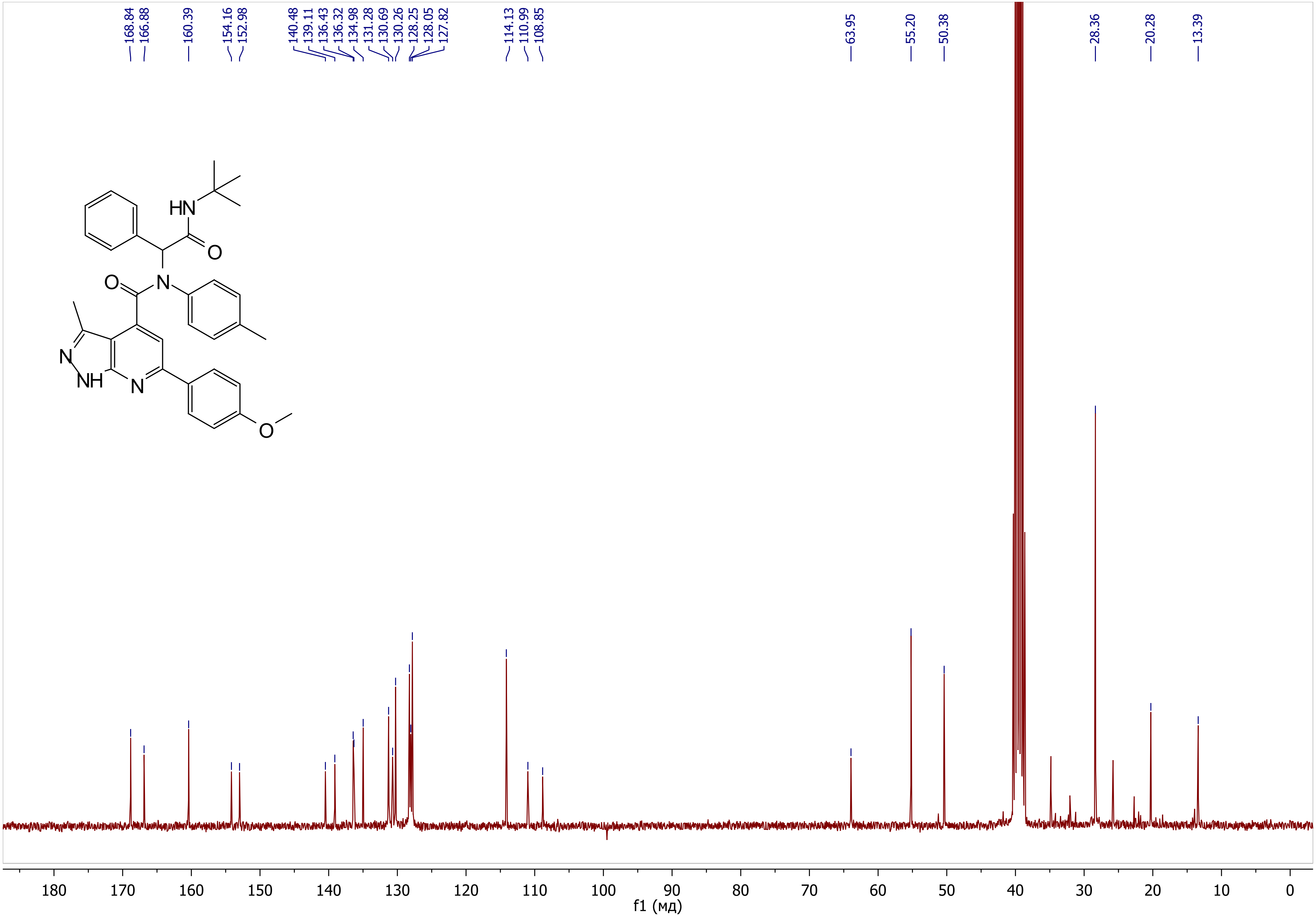
168.84
166.88
160.39
154.16
152.98
140.48
139.11
136.43
136.32
134.98
131.28
130.69
130.26
128.25
128.05
127.82
114.13
110.99
108.85
63.95
55.20
50.38
28.36
20.28
13.39
HN
O
O
N
N
NH
N
O
180 170 160 150 140 130 120 110 100 90 80 70 60 50 40 30 20 10 0
f1 (мд)

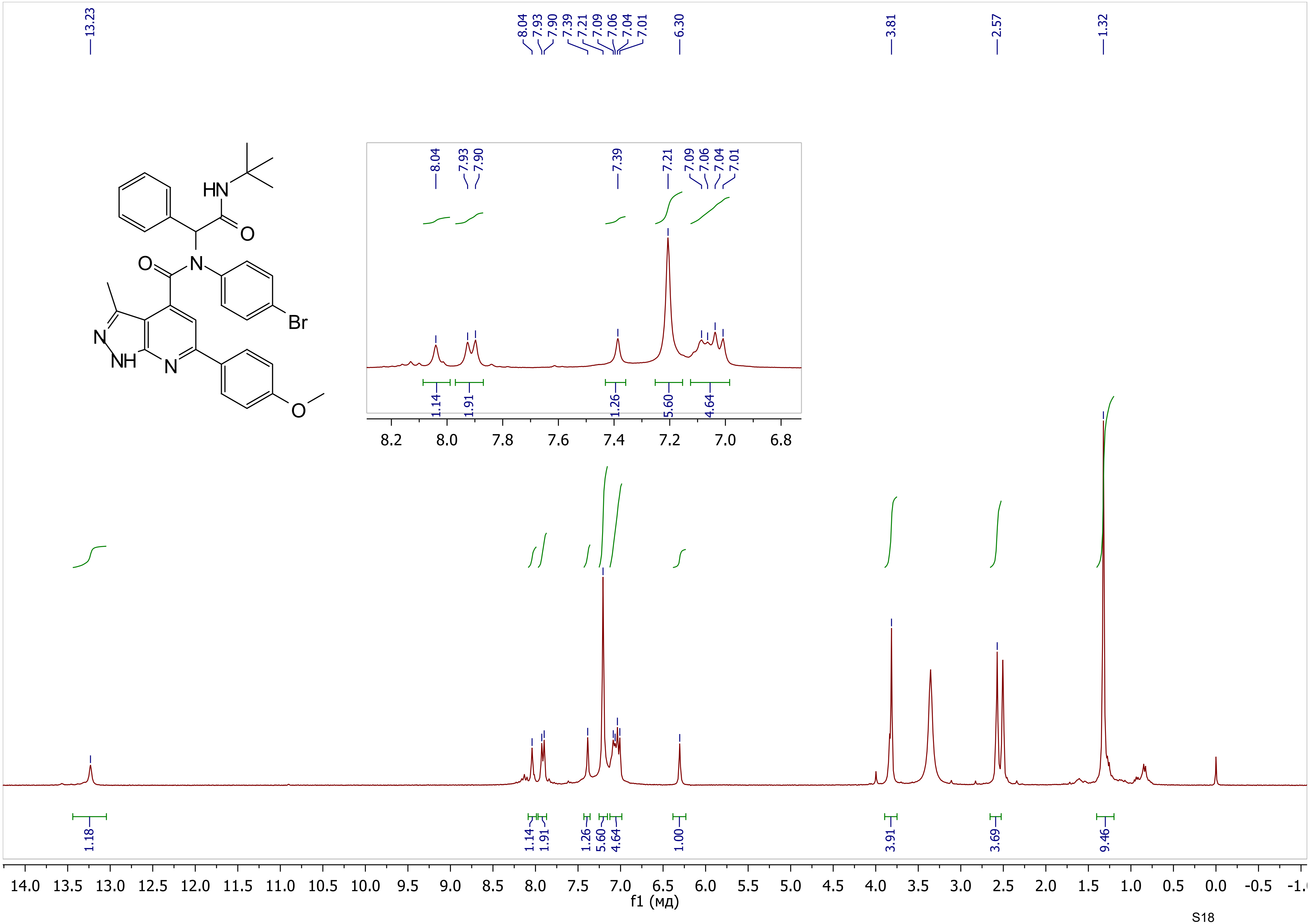
13.23
8.04
7.93
7.90
7.39
7.21
7.09
7.06
7.04
7.01
6.30
3.81
2.57
1.32
HN
O
O
N
N
NH
N
Br
O
1.14
1.91
1.26
5.60
4.64
8.2
8.0
7.8
7.6
7.4
7.2
7.0
6.8
1.18
1.00
3.91
3.69
9.46
14.0 13.5 13.0 12.5 12.0 11.5 11.0 10.5 10.0 9.5 9.0 8.5 8.0 7.5 7.0 6.5 6.0 5.5 5.0 4.5 4.0 3.5 3.0 2.5 2.0 1.5 1.0 0.5 0.0 -0.5
f1 (мд)

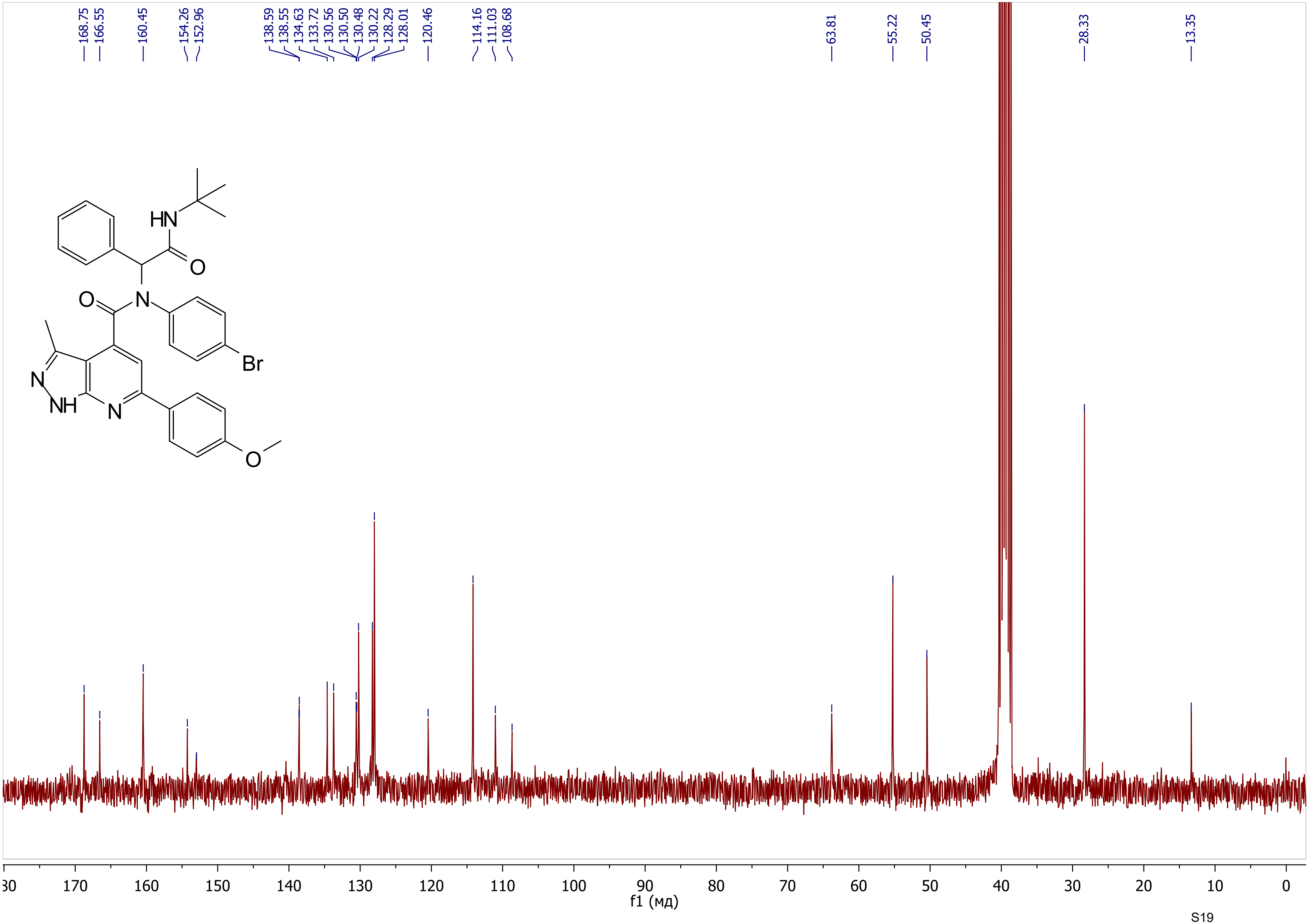
168.75
166.55
160.45
154.26
152.96
138.59
138.55
134.63
133.72
130.56
130.50
130.48
130.22
128.29
128.01
120.46
114.16
111.03
108.68
63.81
55.22
50.45
28.33
13.35
HN
O
O
N
Br
N
NH
N
O
30
170
160
150
140
130
120
110
100
90
80
70
60
50
40
30
20
10
0
f1 (мд)

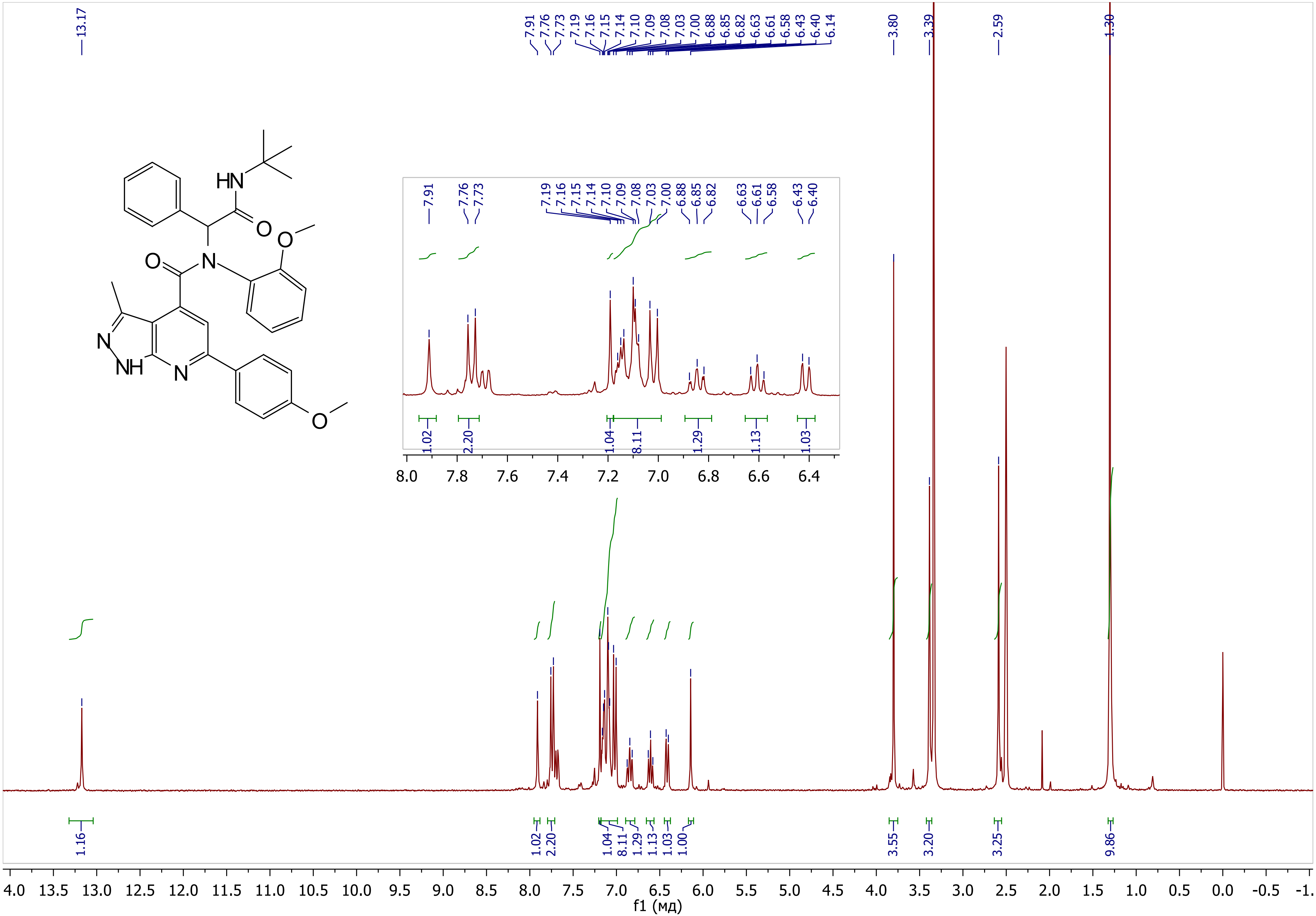
13.17
7.91
7.76
7.73
7.19
7.16
7.15
7.14
7.10
7.09
7.08
7.03
7.00
6.88
6.85
6.82
6.63
6.61
6.58
6.43
6.40
6.14
3.80
3.39
2.59
1.30
HN
O
O
O
N
N
NH
N
O
1.02
2.20
1.04
8.11
1.29
1.13
1.03
8.0
7.8
7.6
7.4
7.2
7.0
6.8
6.6
6.4
1.16
1.00
3.55
3.20
3.25
9.86
f1 (мд)

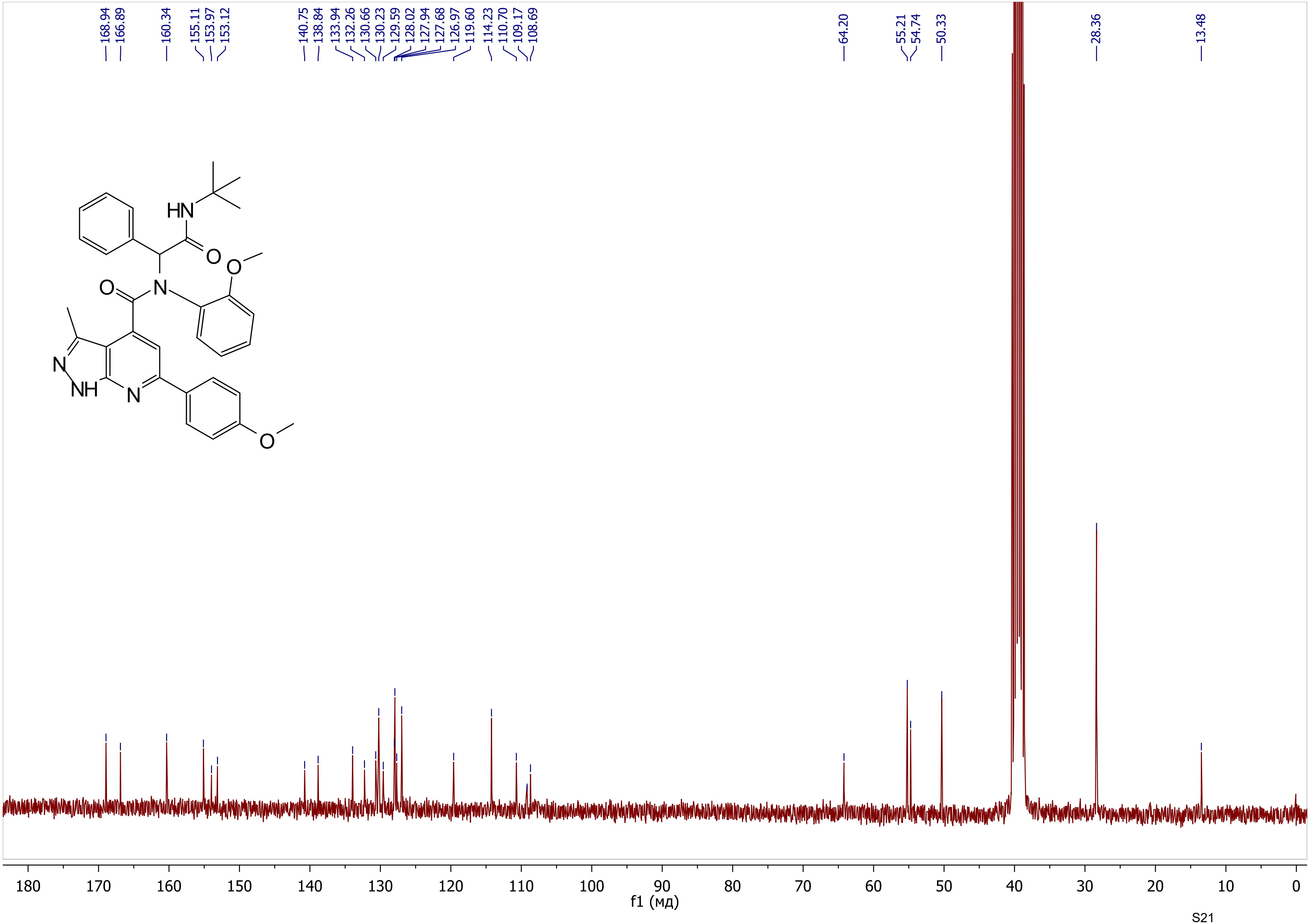
168.94
166.89
160.34
155.11
153.97
153.12
140.75
138.84
133.94
132.26
130.66
130.23
129.59
128.02
127.94
127.68
126.97
119.60
114.23
110.70
109.17
108.69
64.20
55.21
54.74
50.33
28.36
13.48
HN
O
O
N
O
N
NH
N
O
180
170
160
150
140
130
120
110
100
90
80
70
60
50
40
30
20
10
0
f1 (мд)

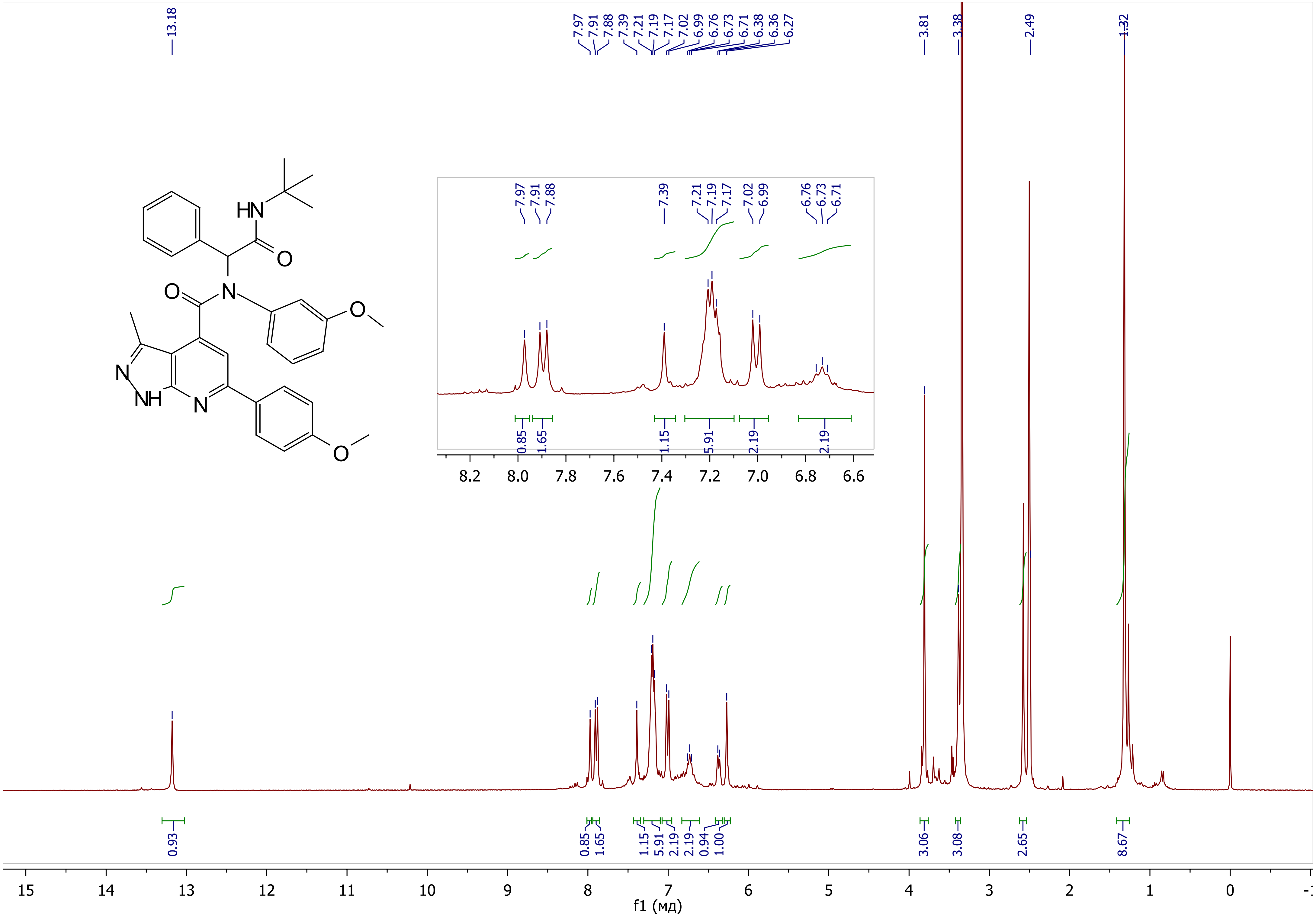
13.18
7.97
7.91
7.88
7.39
7.21
7.19
7.17
7.02
6.99
6.76
6.73
6.71
6.38
6.36
6.27
3.81
3.38
2.49
1.32
HN
O
O
N
O
N
NH
N
O
0.85
1.65
1.15
5.91
2.19
2.19
8.2
8.0
7.8
7.6
7.4
7.2
7.0
6.8
6.6
0.93
0.94
1.00
3.06
3.08
2.65
8.67
15
14
13
12
11
10
9
8
7
6
5
4
3
2
1
0
-1
f1 (мд)

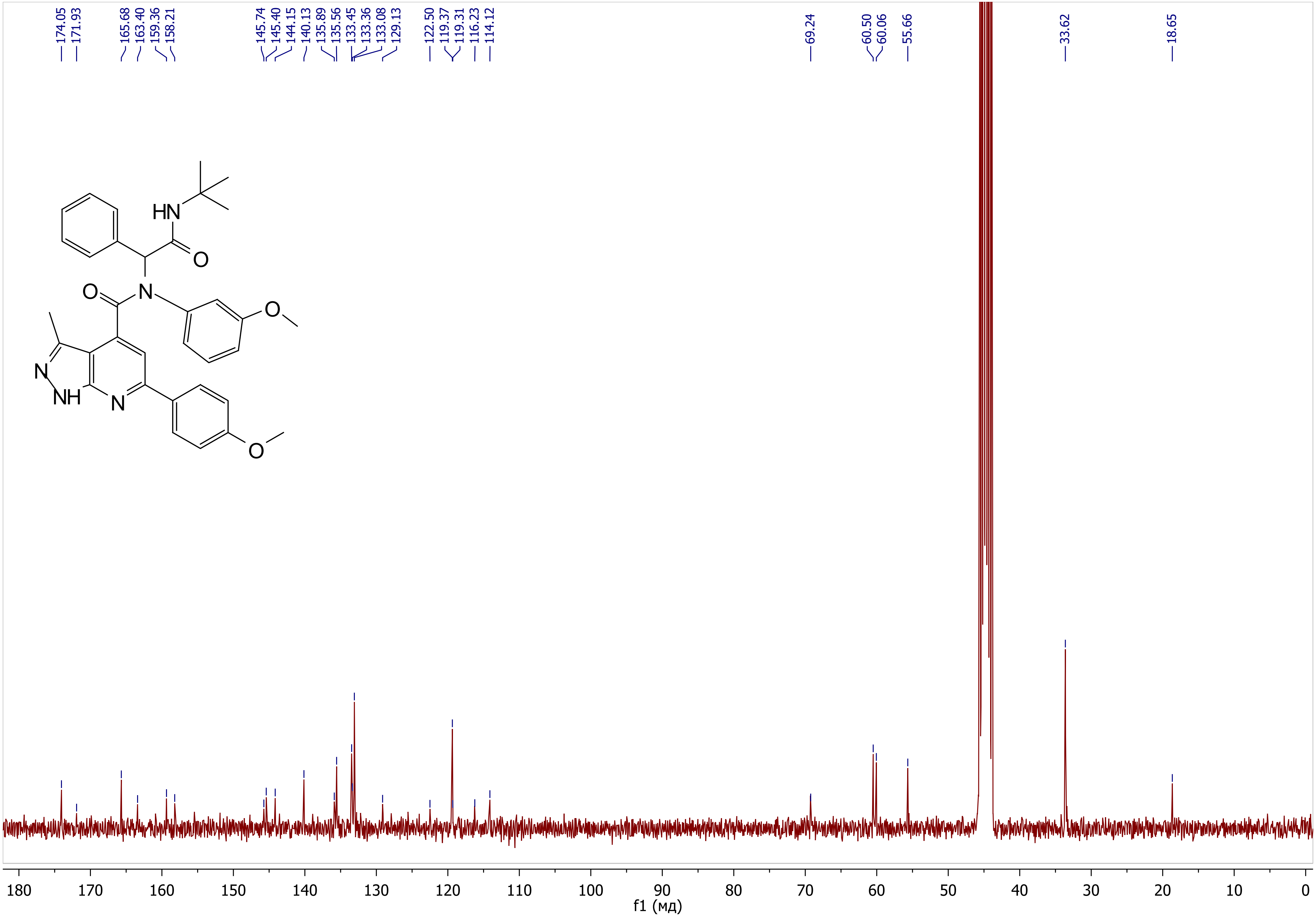
174.05
171.93
165.68
163.40
159.36
158.21
145.74
145.40
144.15
140.13
135.89
135.56
133.45
133.36
133.08
129.13
122.50
119.37
119.31
116.23
114.12
69.24
60.50
60.06
55.66
33.62
18.65
HN
O
N
O
N
NH
N
O
O
180 170 160 150 140 130 120 110 100 90 80 70 60 50 40 30 20 10 0
f1 (мд)

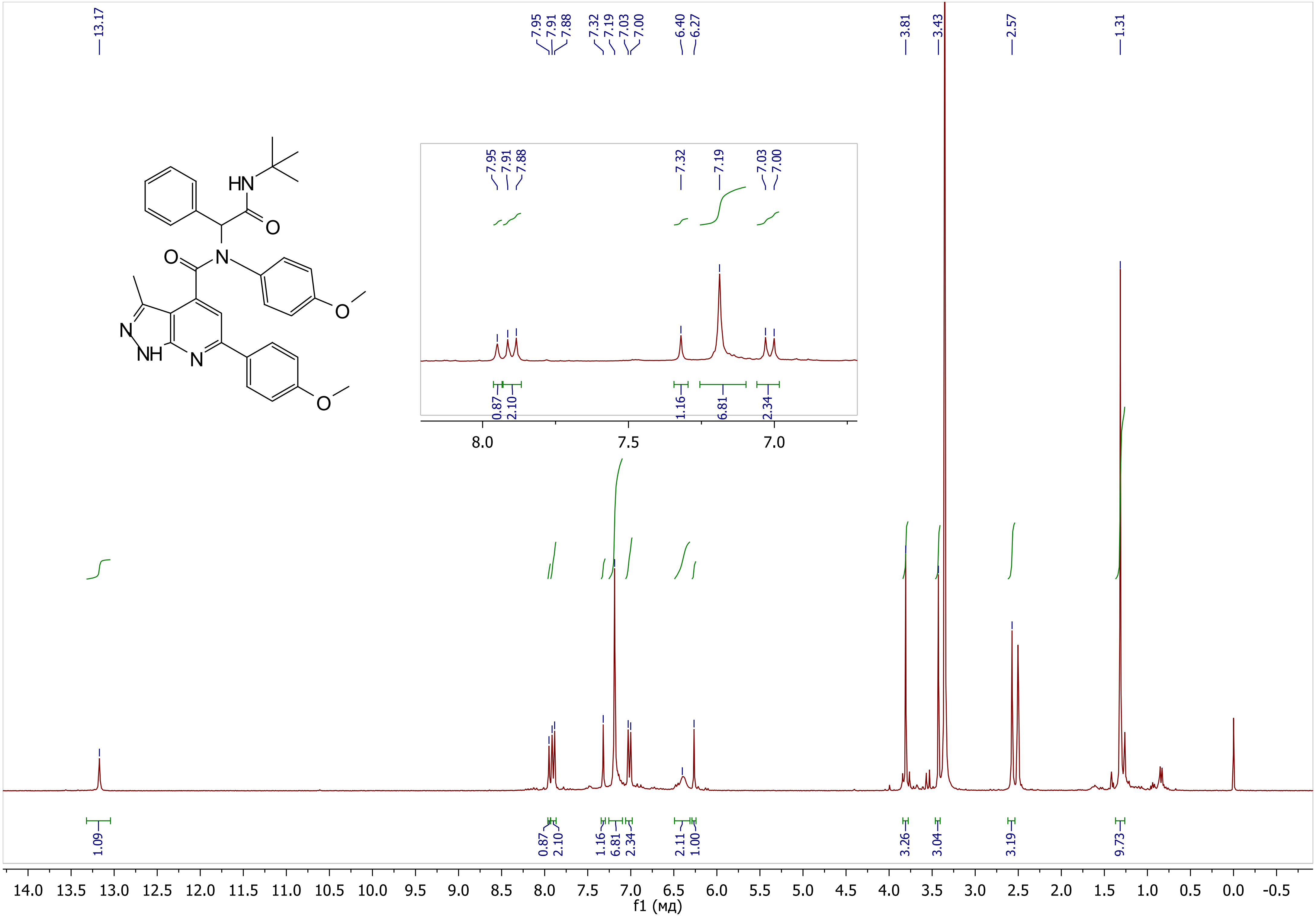
13.17
7.95
7.91
7.88
7.32
7.19
7.03
7.00
6.40
6.27
3.81
3.43
2.57
1.31
HN
O
O
N
N
NH
N
O
O
1.09
0.87
2.10
1.16
6.81
2.34
2.11
1.00
3.26
3.04
3.19
9.73
8.0
7.5
7.0
14.0 13.5 13.0 12.5 12.0 11.5 11.0 10.5 10.0 9.5 9.0 8.5 8.0 7.5 7.0 6.5 6.0 5.5 5.0 4.5 4.0 3.5 3.0 2.5 2.0 1.5 1.0 0.5 0.0 -0.5
f1 (мд)

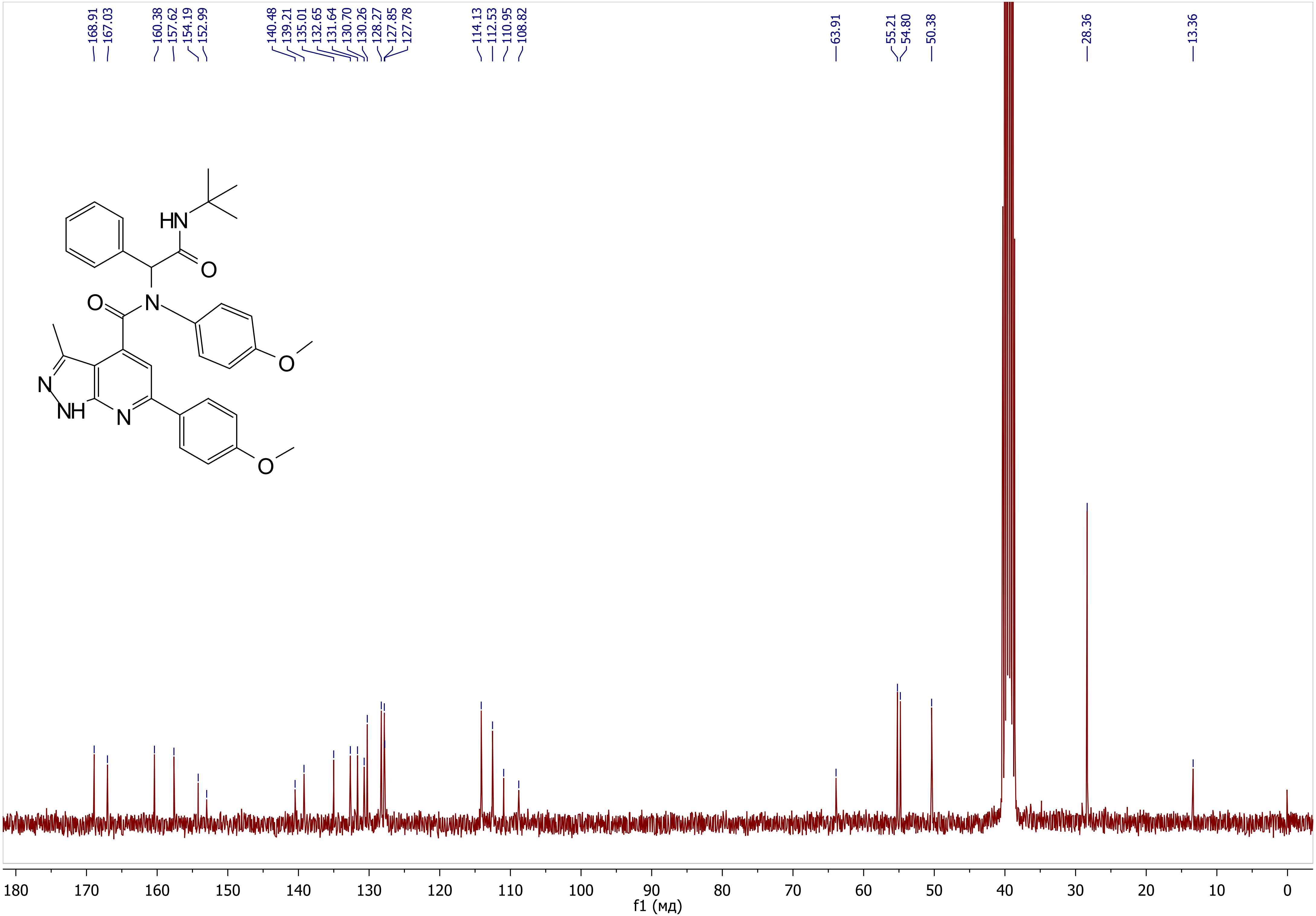
168.91
167.03
160.38
157.62
154.19
152.99
140.48
139.21
135.01
132.65
131.64
130.70
130.26
128.27
127.85
127.78
114.13
112.53
110.95
108.82
63.91
55.21
54.80
50.38
28.36
13.36
HN
O
N
O
N
NH
N
O
O
180 170 160 150 140 130 120 110 100 90 80 70 60 50 40 30 20 10 0
f1 (мд)

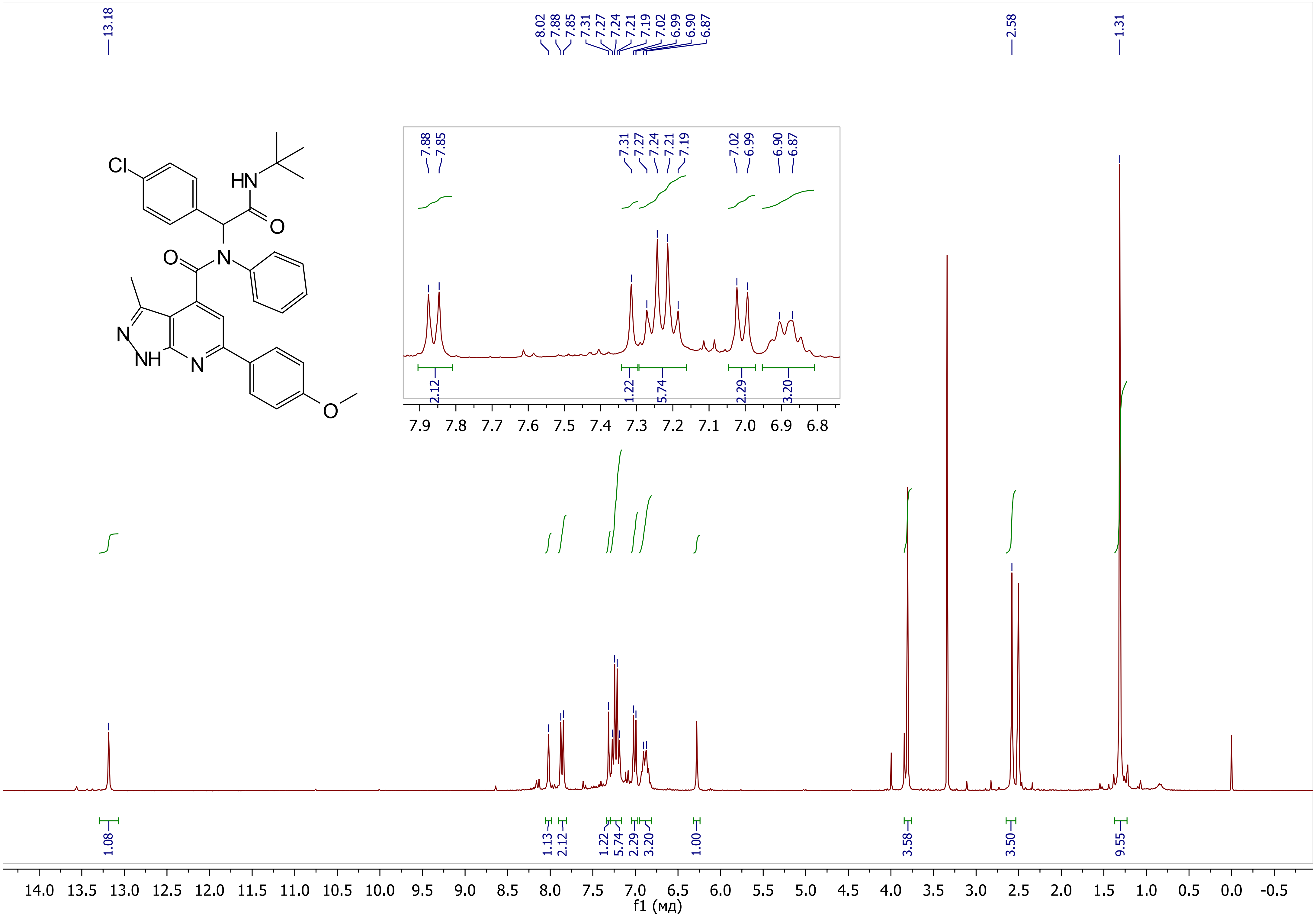
13.18
8.02
7.88
7.85
7.31
7.27
7.24
7.21
7.19
7.02
6.99
6.90
6.87
2.58
1.31
Cl
HN
O
O
N
N
NH
N
O
1.08
1.13
2.12
1.22
5.74
2.29
3.20
1.00
3.58
3.50
9.55
14.0 13.5 13.0 12.5 12.0 11.5 11.0 10.5 10.0 9.5 9.0 8.5 8.0 7.5 7.0 6.5 6.0 5.5 5.0 4.5 4.0 3.5 3.0 2.5 2.0 1.5 1.0 0.5 0.0 -0.5
f1 (мд)
7.9 7.8 7.7 7.6 7.5 7.4 7.3 7.2 7.1 7.0 6.9 6.8

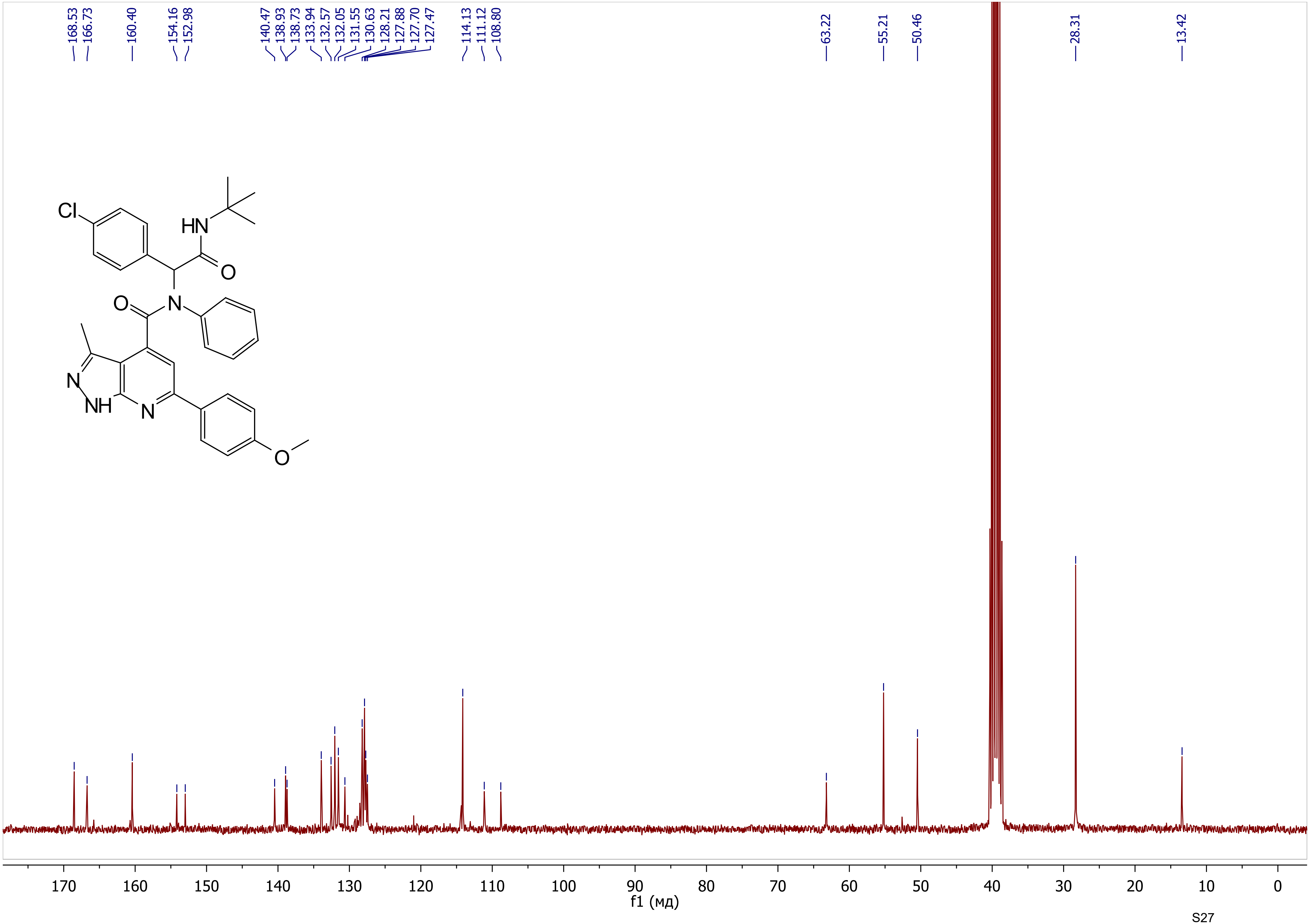
168.53
166.73
160.40
154.16
152.98
140.47
138.93
138.73
133.94
132.57
132.05
131.55
130.63
128.21
127.88
127.70
127.47
114.13
111.12
108.80
63.22
55.21
50.46
28.31
13.42
Cl
HN
O
O
N
N
NH
N
O
170
160
150
140
130
120
110
100
90
80
70
60
50
40
30
20
10
0
f1 (мд)

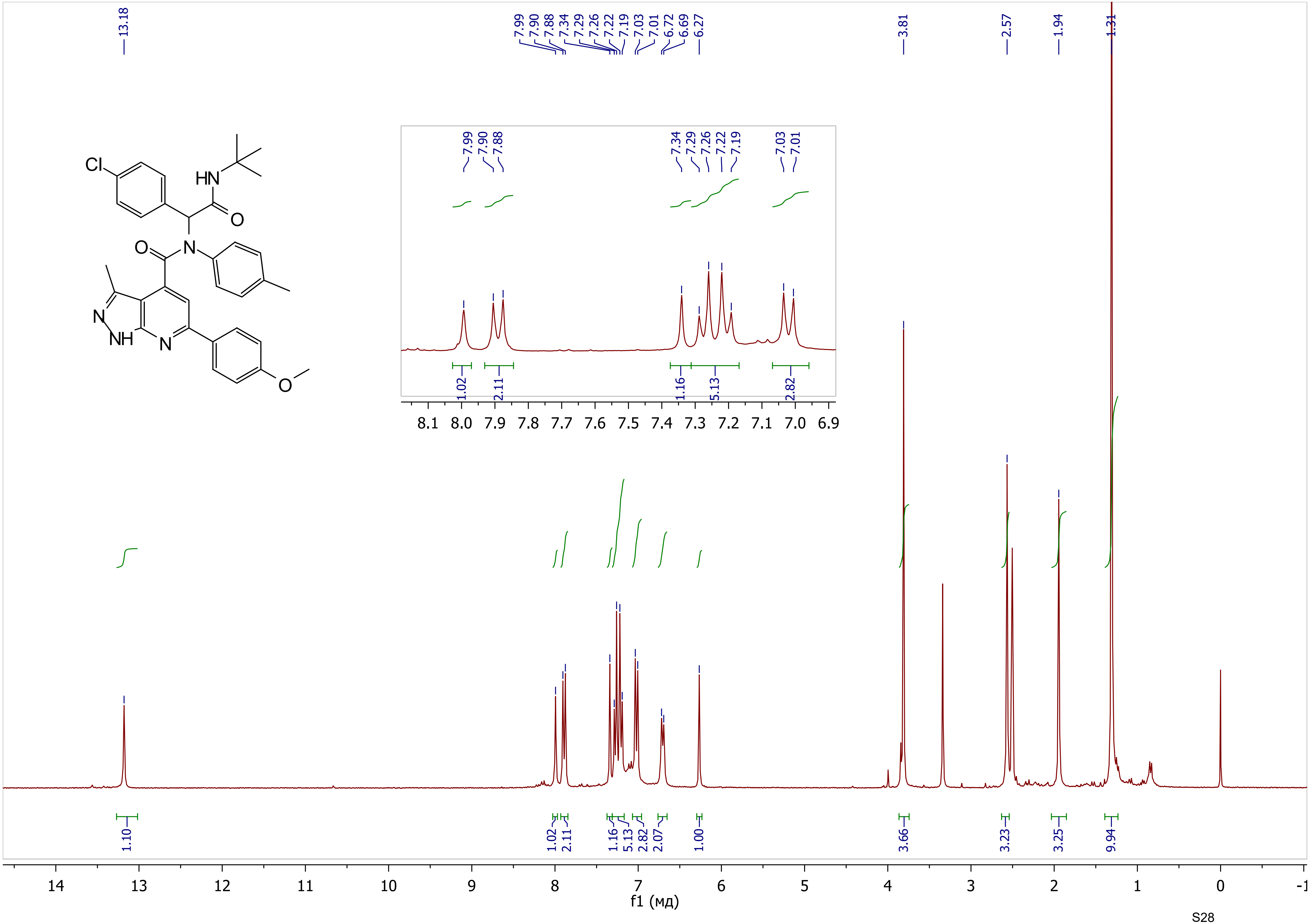
13.18
7.99
7.90
7.88
7.34
7.29
7.26
7.22
7.19
7.03
7.01
6.72
6.69
6.27
3.81
2.57
1.94
1.31
1.10
1.02
2.11
1.16
5.13
2.82
2.07
1.00
3.66
3.23
3.25
9.94
14 13 12 11 10 9 8 7 6 5 4 3 2 1 0 -1
f1 (мд)
8.1 8.0 7.9 7.8 7.7 7.6 7.5 7.4 7.3 7.2 7.1 7.0 6.9
Cl
HN
O
O
N
N
NH
N
O

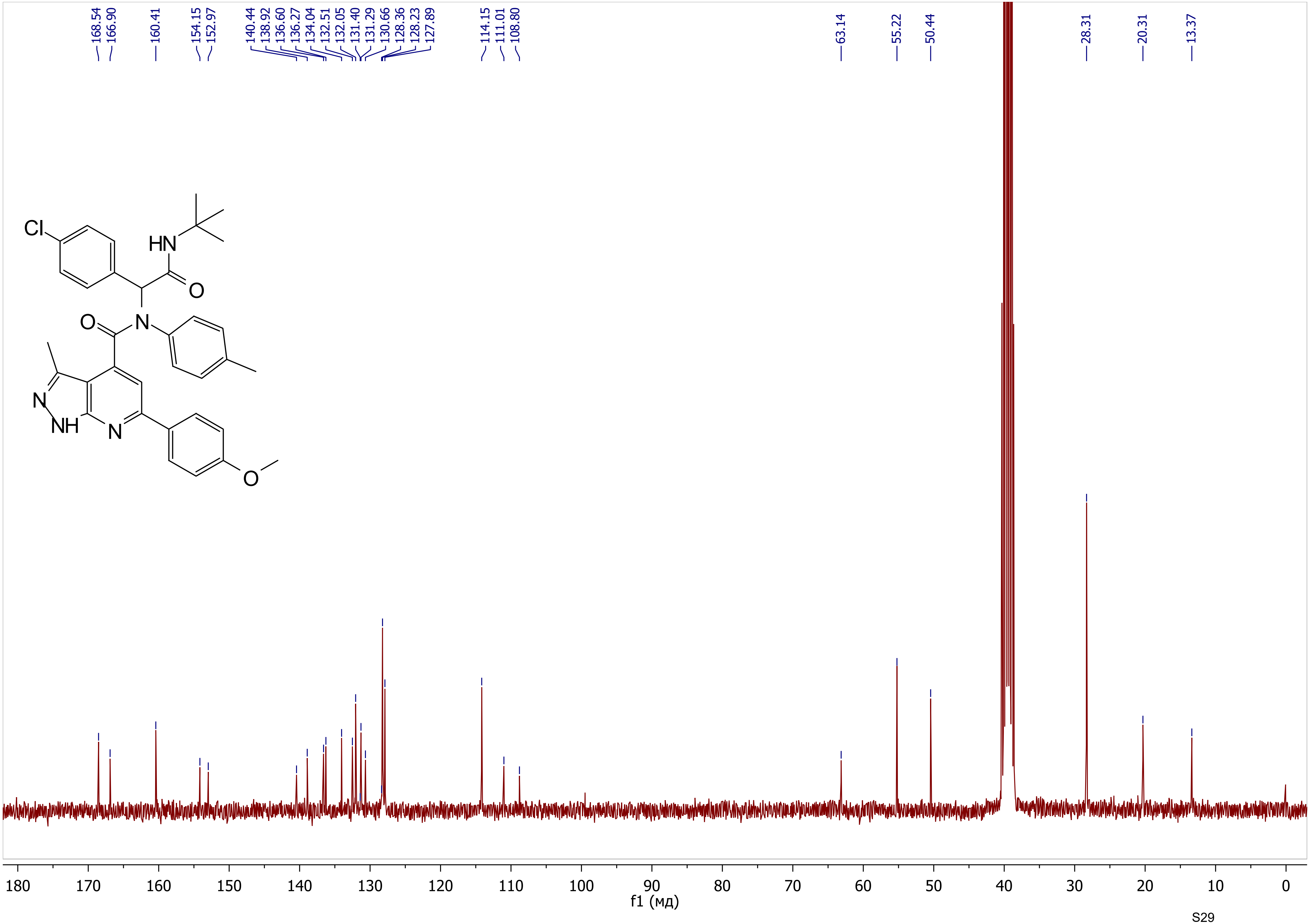
168.54
166.90
160.41
154.15
152.97
140.44
138.92
136.60
136.27
134.04
132.51
132.05
131.40
131.29
130.66
128.36
128.23
127.89
114.15
111.01
108.80
63.14
55.22
50.44
28.31
20.31
13.37
Cl
HN
O
O
N
N
NH
N
O
180 170 160 150 140 130 120 110 100 90 80 70 60 50 40 30 20 10 0
f1 (мд)

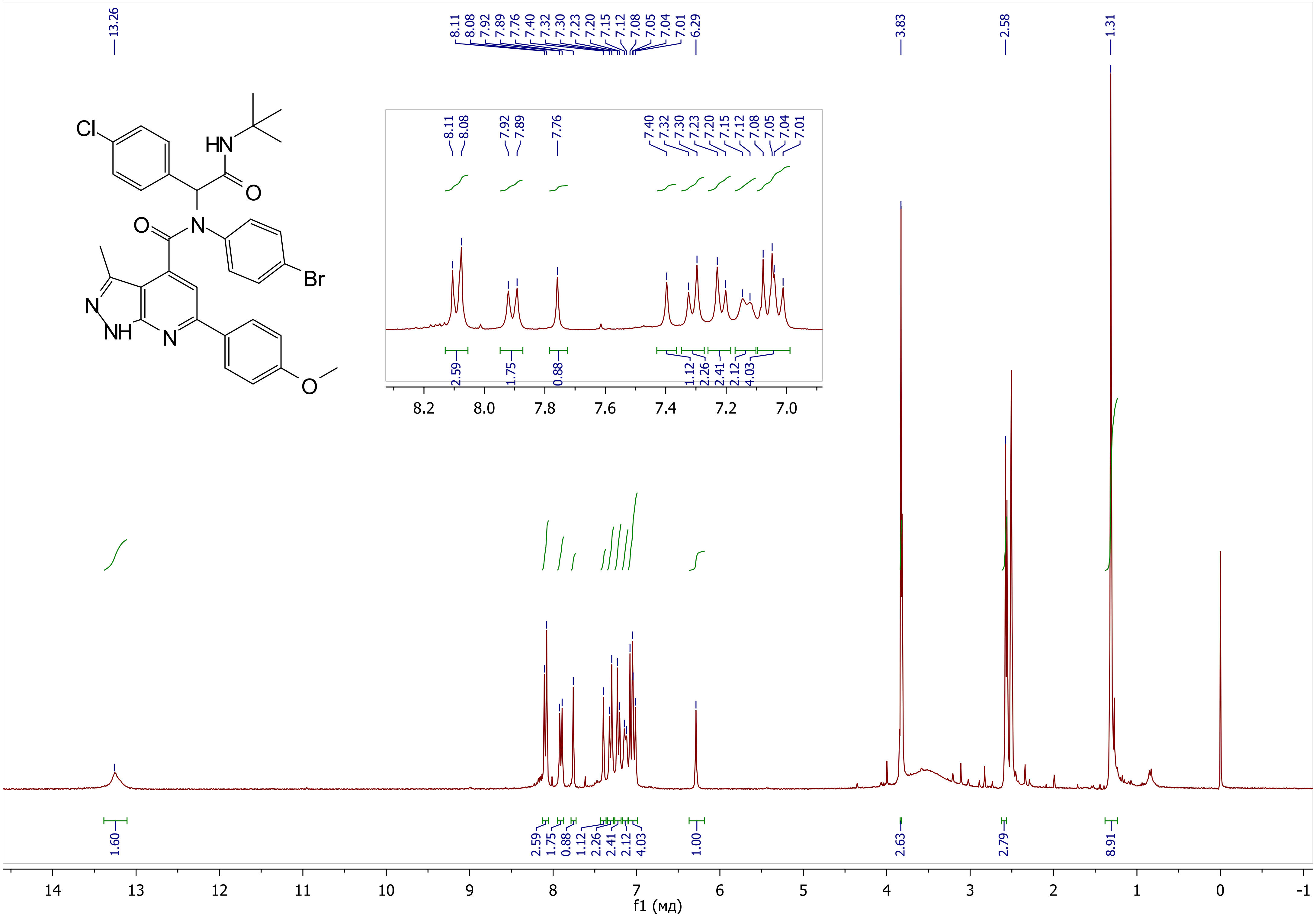

13.26
8.11 8.08 7.92 7.89 7.76 7.40 7.32 7.30 7.23 7.20 7.15 7.12 7.08 7.05 7.04 7.01 6.29
3.83
2.58
1.31
Cl
HN
O
O
N
Br
N
NH
N
O
2.59 1.75 0.88 1.12 2.26 2.41 2.12 4.03
1.60
1.00
2.63
2.79
8.91
8.2 8.0 7.8 7.6 7.4 7.2 7.0
14 13 12 11 10 9 8 7 6 5 4 3 2 1 0 -1
f1 (мд)

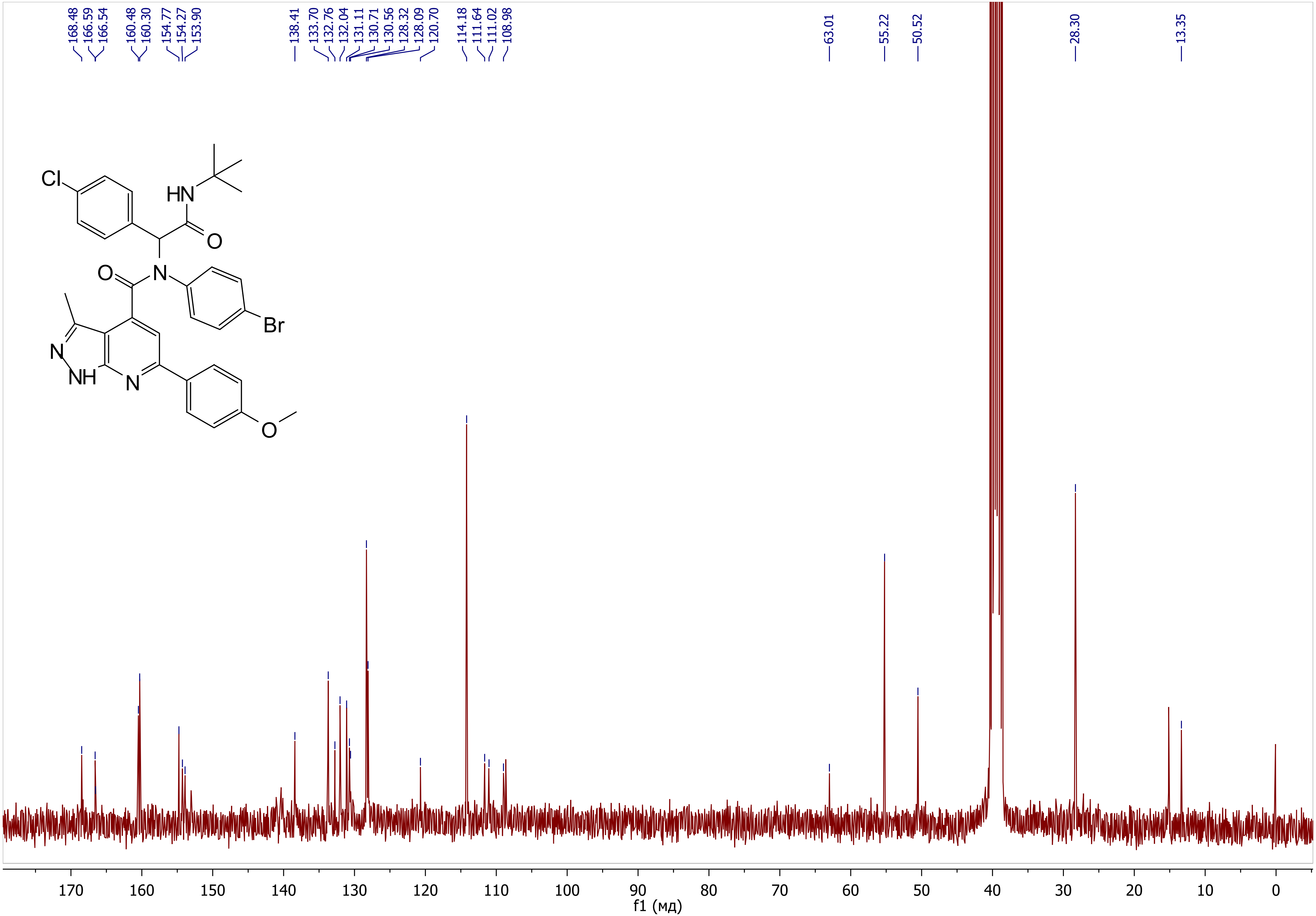
168.48
166.59
166.54
160.48
160.30
154.77
154.27
153.90
138.41
133.70
132.76
132.04
131.11
130.71
130.56
128.32
128.09
120.70
114.18
111.64
111.02
108.98
63.01
55.22
50.52
28.30
13.35
Cl
HN
O
O
N
Br
N
NH
N
O
170
160
150
140
130
120
110
100
90
80
70
60
50
40
30
20
10
0
f1 (мд)

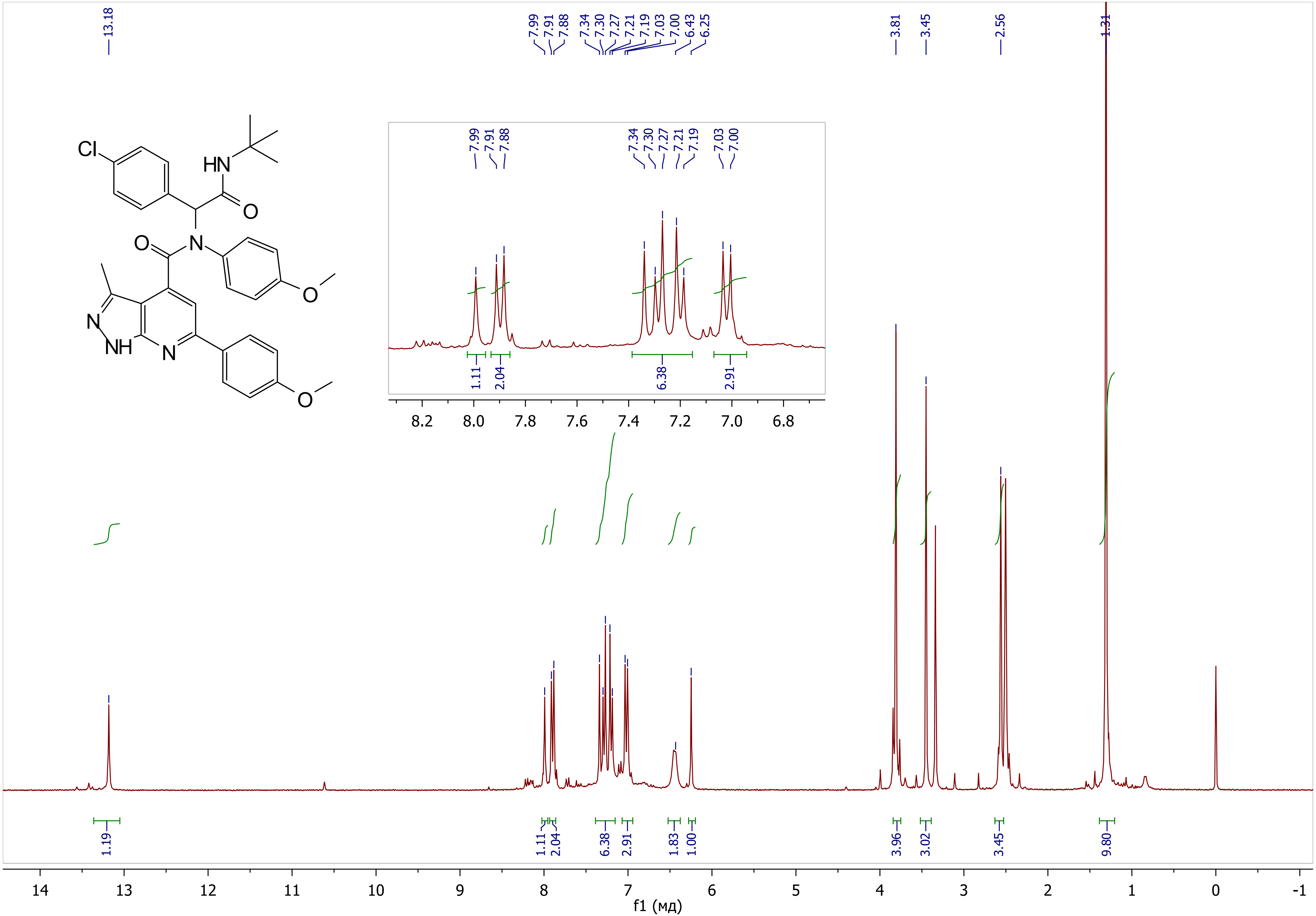
13.18
7.99
7.91
7.88
7.34
7.30
7.27
7.21
7.19
7.03
7.00
6.43
6.25
3.81
3.45
2.56
1.31
Cl
HN
O
O
N
N
NH
N
O
O
1.19
1.11
2.04
6.38
2.91
1.83
1.00
3.96
3.02
3.45
9.80
14
13
12
11
10
9
8
7
6
5
4
3
2
1
0
-1
f1 (мд)
8.2
8.0
7.8
7.6
7.4
7.2
7.0
6.8

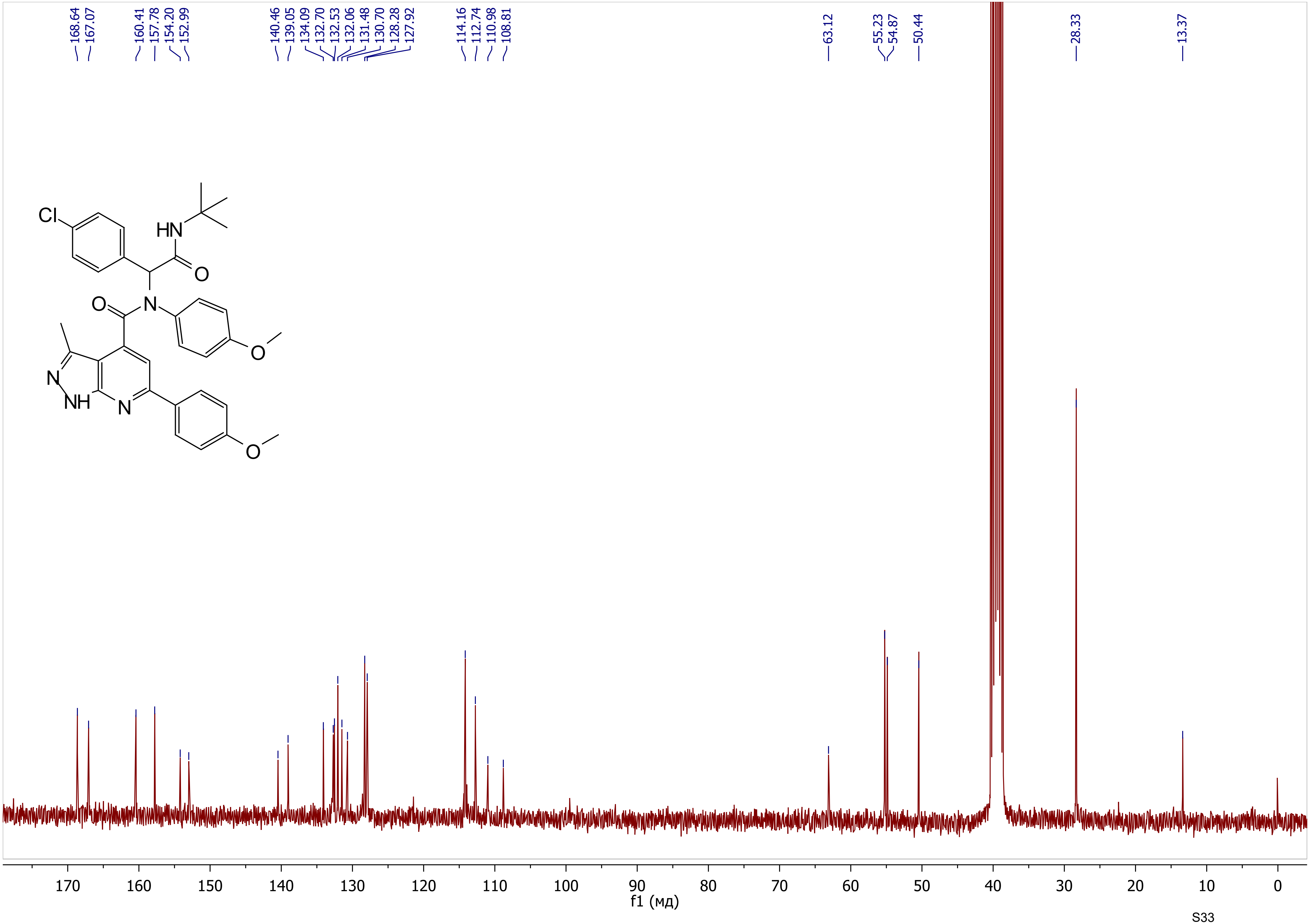

168.64
167.07
160.41
157.78
154.20
152.99
140.46
139.05
134.09
132.70
132.53
132.06
131.48
130.70
128.28
127.92
114.16
112.74
110.98
108.81
63.12
55.23
54.87
50.44
28.33
13.37
Cl
HN
O
O
N
N
NH
N
O
O
170
160
150
140
130
120
110
100
90
80
70
60
50
40
30
20
10
0
f1 (мд)

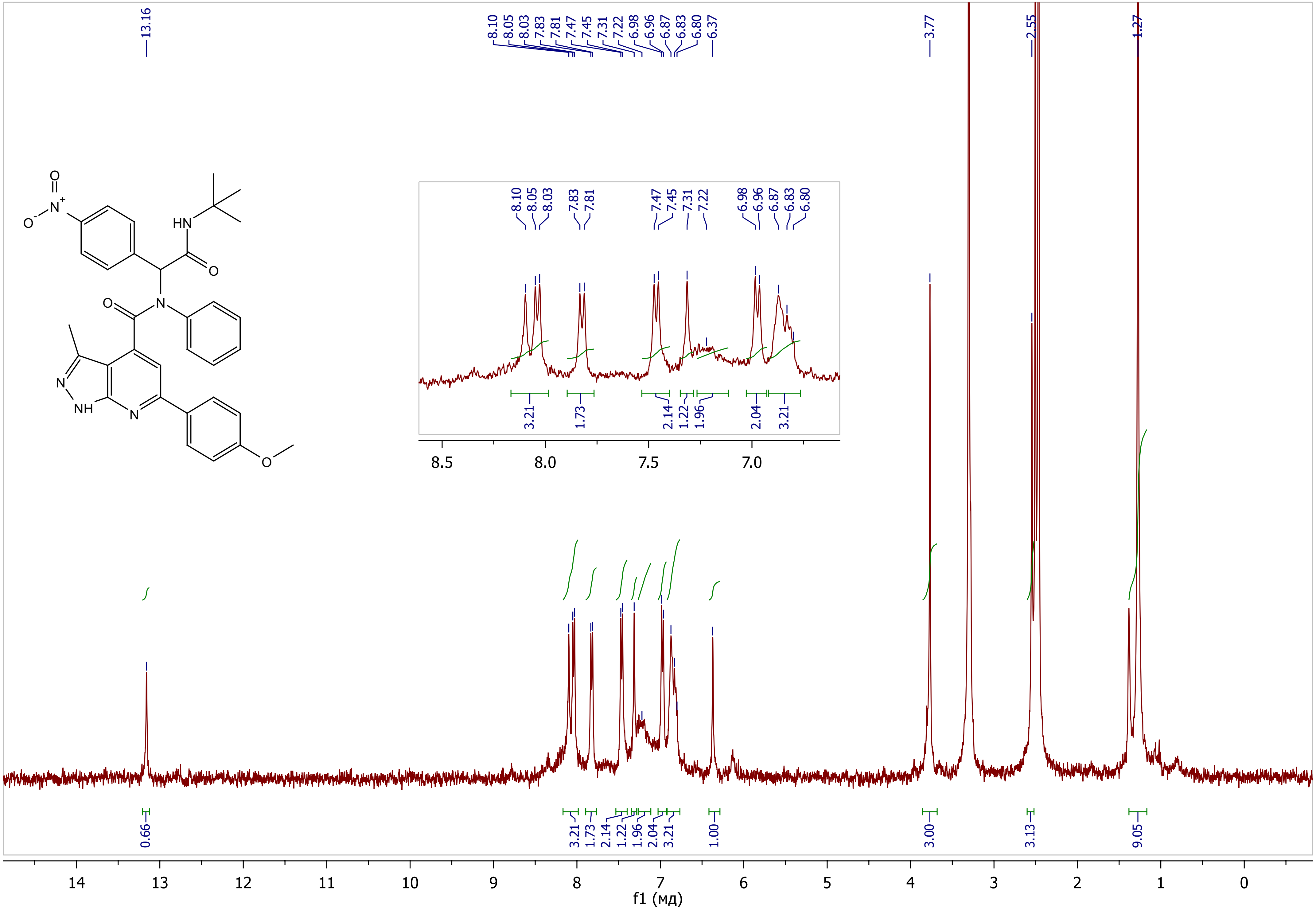
13.16
8.10 8.05 8.03 7.83 7.81 7.47 7.45 7.31 7.22 6.98 6.96 6.87 6.83 6.80 6.37
3.77
2.55
1.27
O
N^+
O^-
HN
O
O
N
N
NH
N
O
8.10 8.05 8.03 7.83 7.81 7.47 7.45 7.31 7.22 6.98 6.96 6.87 6.83 6.80
3.21 1.73 2.14 1.22 1.96 2.04 3.21
8.5 8.0 7.5 7.0
0.66 3.21 1.73 2.14 1.22 1.96 2.04 3.21 1.00 3.00 3.13 9.05
14 13 12 11 10 9 8 7 6 5 4 3 2 1 0
f1 (мд)

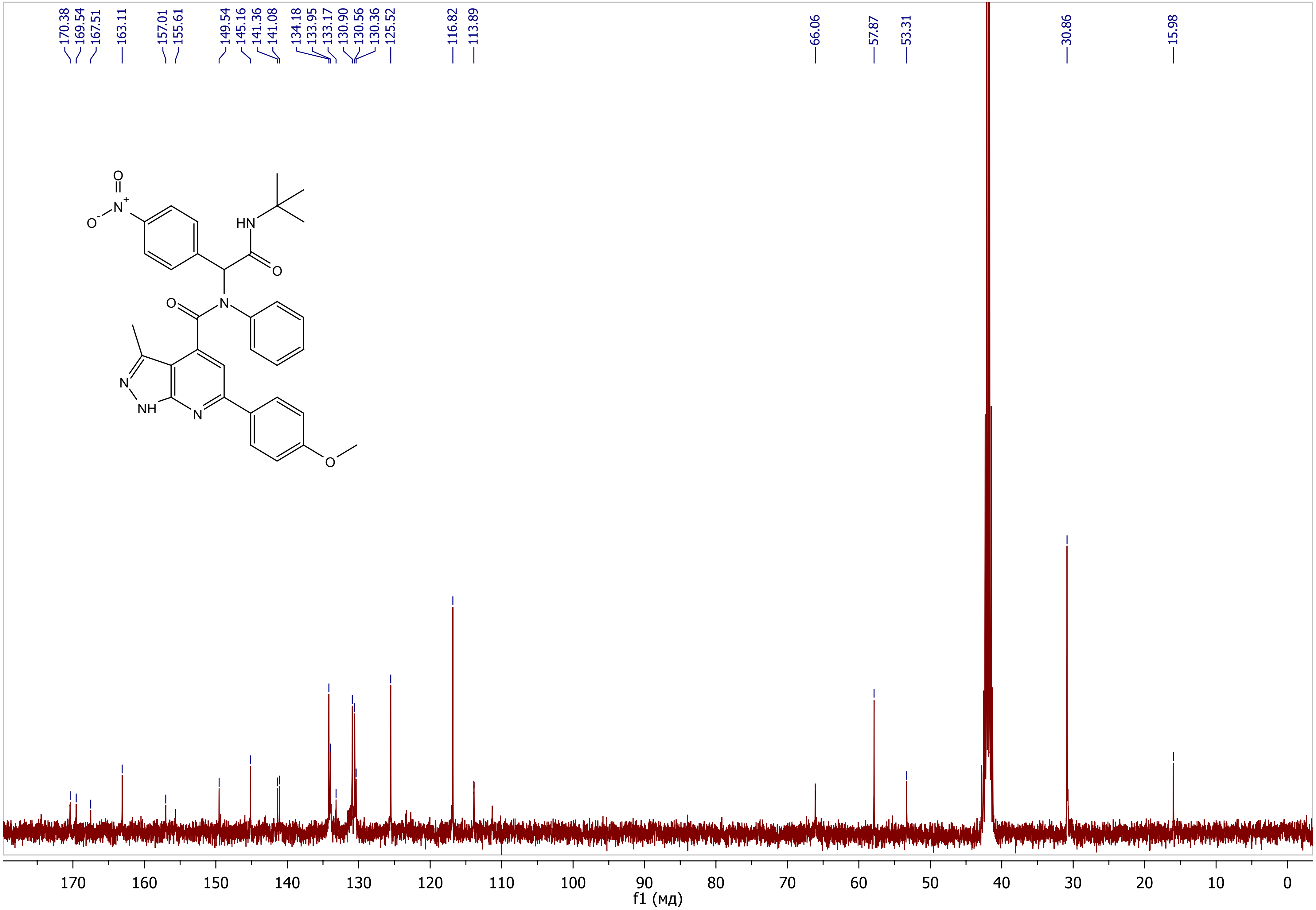
170.38
169.54
167.51
163.11
157.01
155.61
149.54
145.16
141.36
141.08
134.18
133.95
133.17
130.90
130.56
130.36
125.52
116.82
113.89
66.06
57.87
53.31
30.86
15.98
f1 (мд)

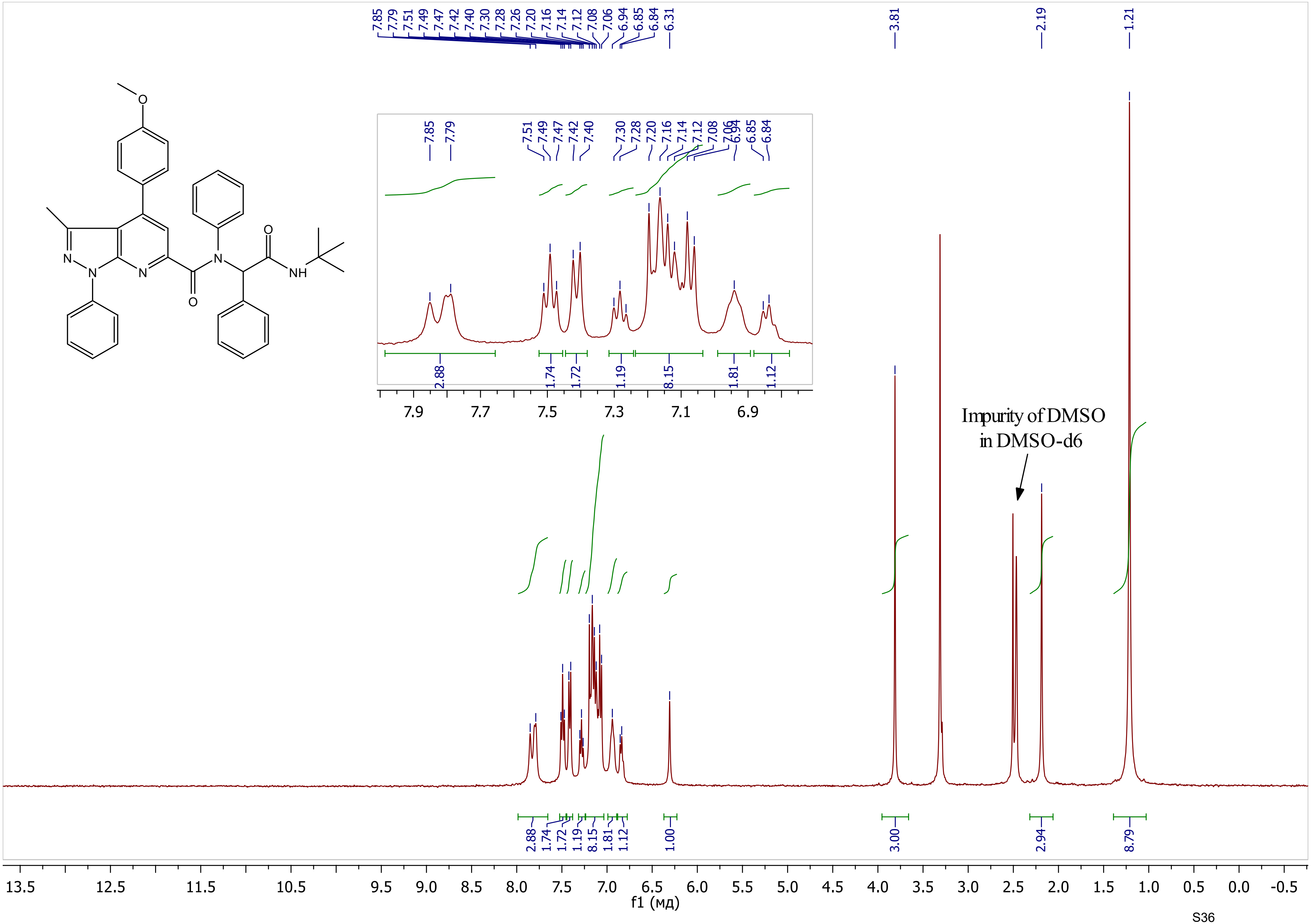

7.85 7.79 7.51 7.49 7.47 7.42 7.40 7.30 7.28 7.26 7.20 7.16 7.14 7.12 7.08 7.06 6.94 6.85 6.84 6.31
3.81
2.19
1.21
2.88 1.74 1.72 1.19 8.15 1.81 1.12
7.9 7.7 7.5 7.3 7.1 6.9
Impurity of DMSO in DMSO-d6
2.88 1.74 1.72 1.19 8.15 1.81 1.12 1.00 3.00 2.94 8.79
13.5 12.5 11.5 10.5 9.5 9.0 8.5 8.0 7.5 7.0 6.5 6.0 5.5 5.0 4.5 4.0 3.5 3.0 2.5 2.0 1.5 1.0 0.5 0.0 -0.5
f1 (мд)

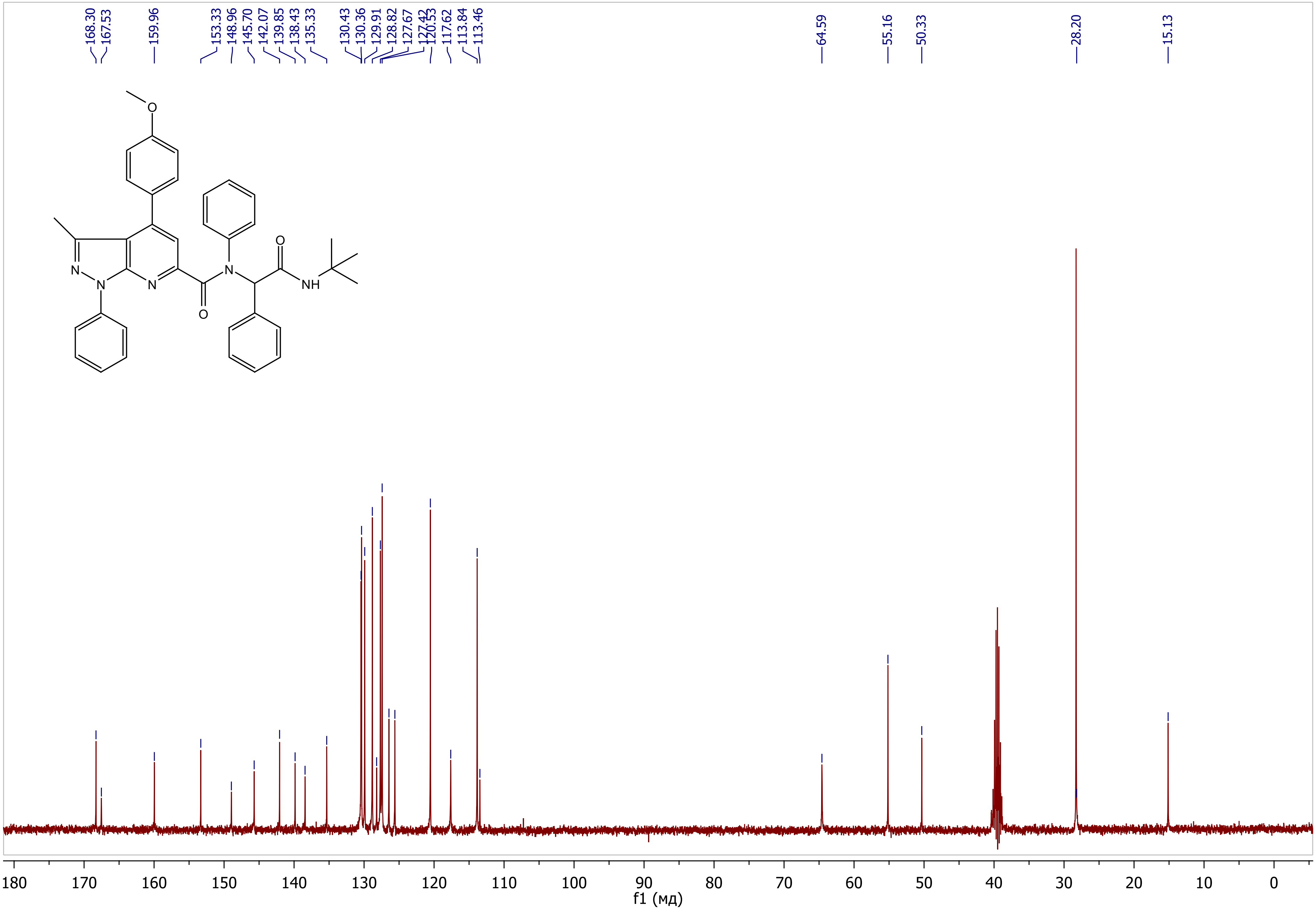
168.30
167.53
159.96
153.33
148.96
145.70
142.07
139.85
138.43
135.33
130.43
130.36
129.91
128.82
127.67
127.42
120.53
117.62
113.84
113.46
64.59
55.16
50.33
28.20
15.13
O
N
N
N
N
O
O
NH
180
170
160
150
140
130
120
110
100
90
80
70
60
50
40
30
20
10
0
f1 (мд)

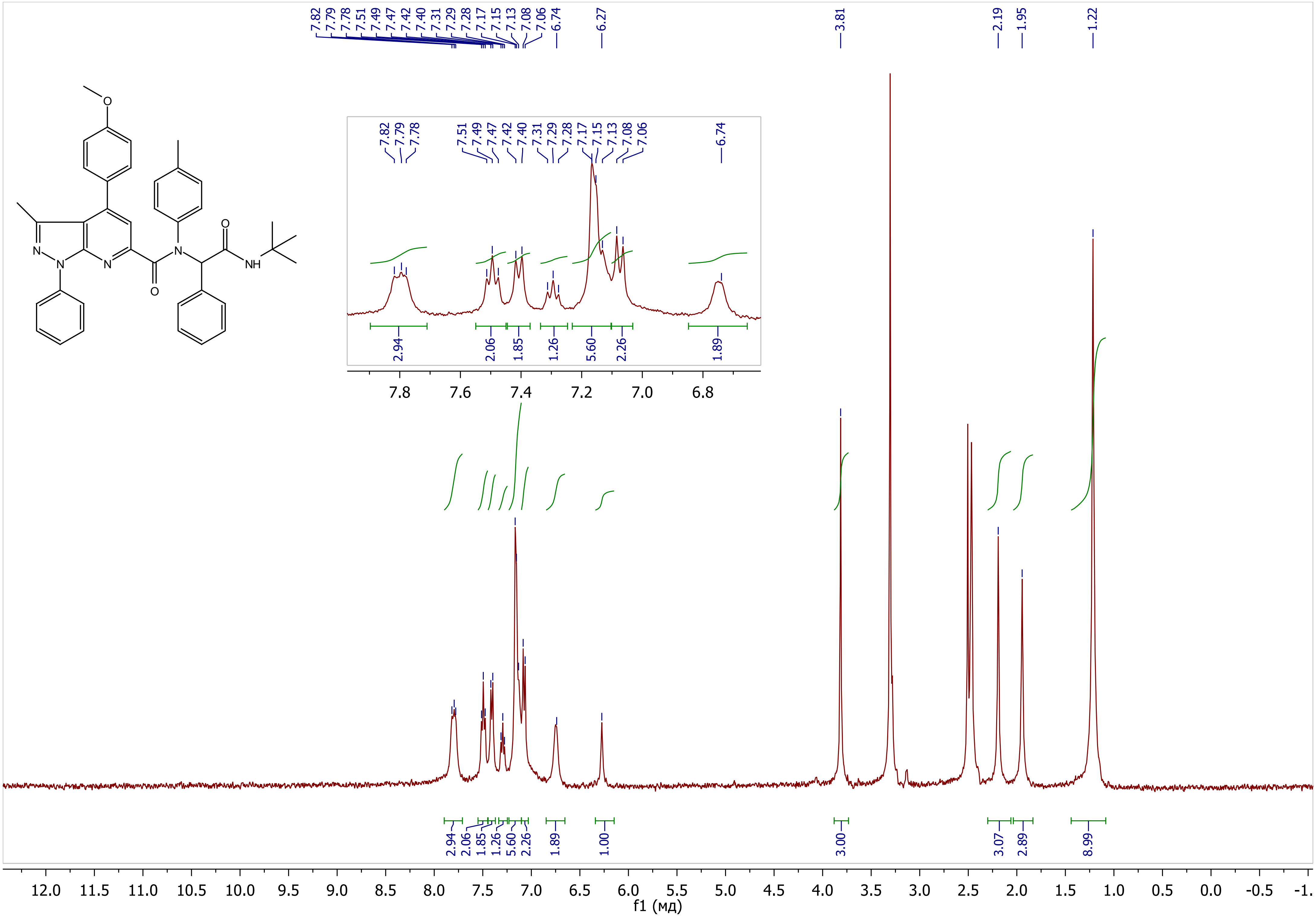
7.82 7.79 7.78 7.51 7.49 7.47 7.42 7.40 7.31 7.29 7.28 7.17 7.15 7.13 7.08 7.06 6.74 6.27 3.81 2.19 1.95 1.22
2.94 2.06 1.85 1.26 5.60 2.26 1.89 1.00 3.00 3.07 2.89 8.99
O
N
N
N
O
N
O
NH
f1 (мд)

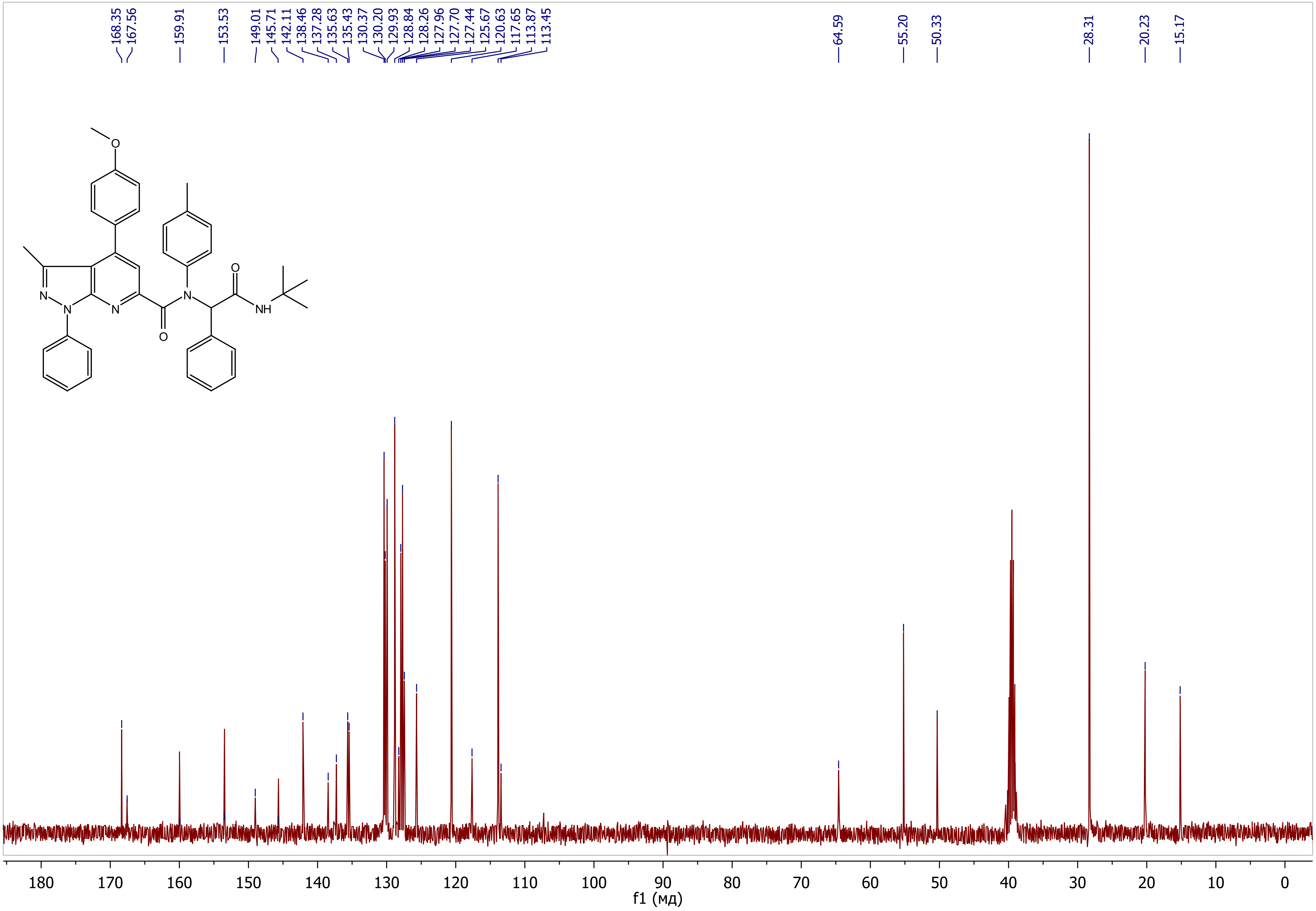

168.35
167.56
159.91
153.53
149.01
145.71
142.11
138.46
137.28
135.63
135.43
130.37
130.20
129.93
128.84
128.26
127.96
127.70
127.44
125.67
120.63
117.65
113.87
113.45
64.59
55.20
50.33
28.31
20.23
15.17
O
N
N
N
O
N
O
NH
180 170 160 150 140 130 120 110 100 90 80 70 60 50 40 30 20 10 0
f1 (мд)

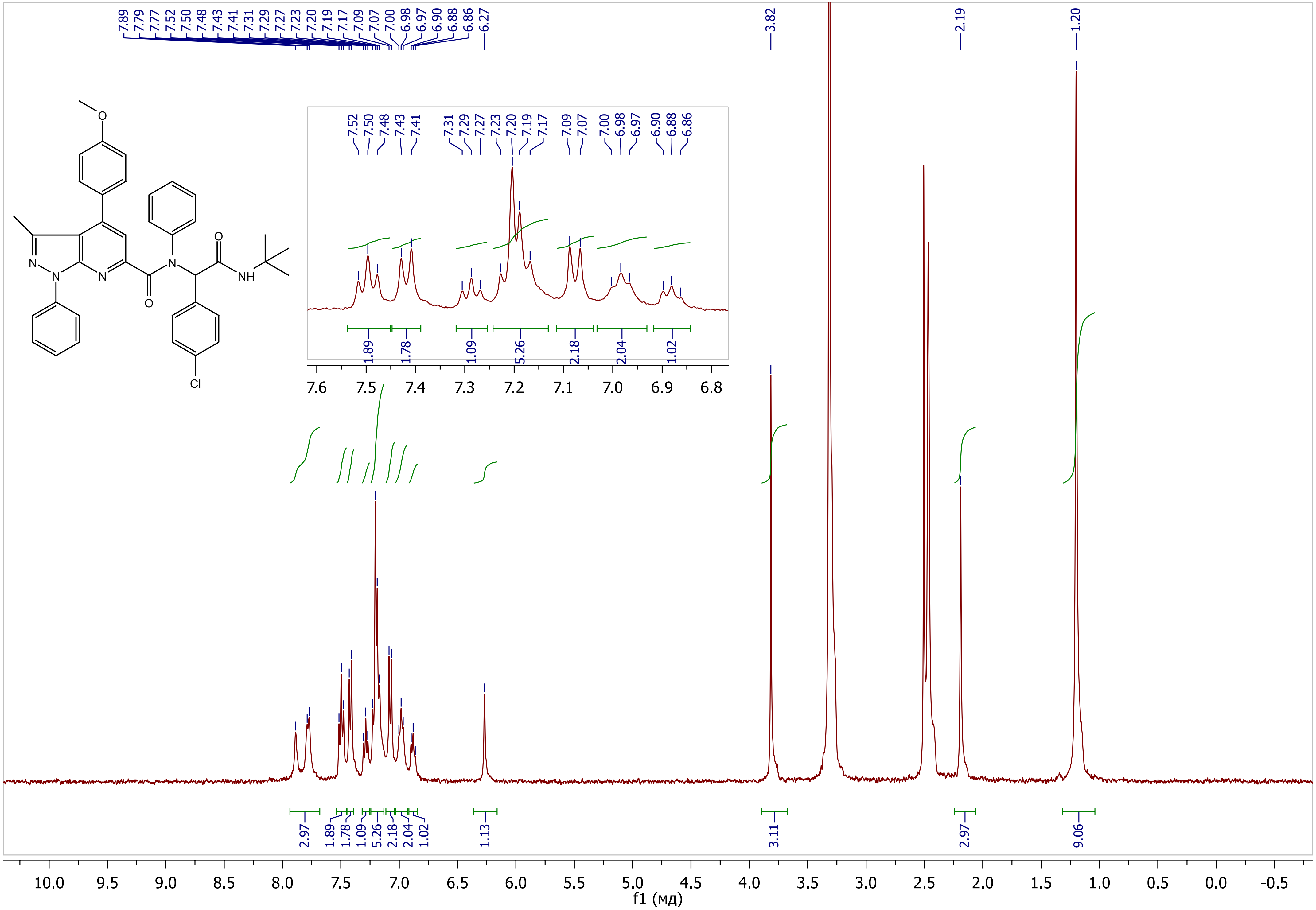
7.89 7.79 7.77 7.52 7.50 7.48 7.43 7.41 7.31 7.29 7.27 7.23 7.20 7.19 7.17 7.09 7.07 7.00 6.98 6.97 6.90 6.88 6.86 6.27
3.82
2.19
1.20
2.97 1.89 1.78 1.09 5.26 2.18 2.04 1.02 1.13 3.11 2.97 9.06
7.52 7.50 7.48 7.43 7.41 7.31 7.29 7.27 7.23 7.20 7.19 7.17 7.09 7.07 7.00 6.98 6.97 6.90 6.88 6.86
1.89 1.78 1.09 5.26 2.18 2.04 1.02
7.6 7.5 7.4 7.3 7.2 7.1 7.0 6.9 6.8
O
N
N
N
O
N
O
NH
O
Cl
10.0 9.5 9.0 8.5 8.0 7.5 7.0 6.5 6.0 5.5 5.0 4.5 4.0 3.5 3.0 2.5 2.0 1.5 1.0 0.5 0.0 -0.5
f1 (мд)

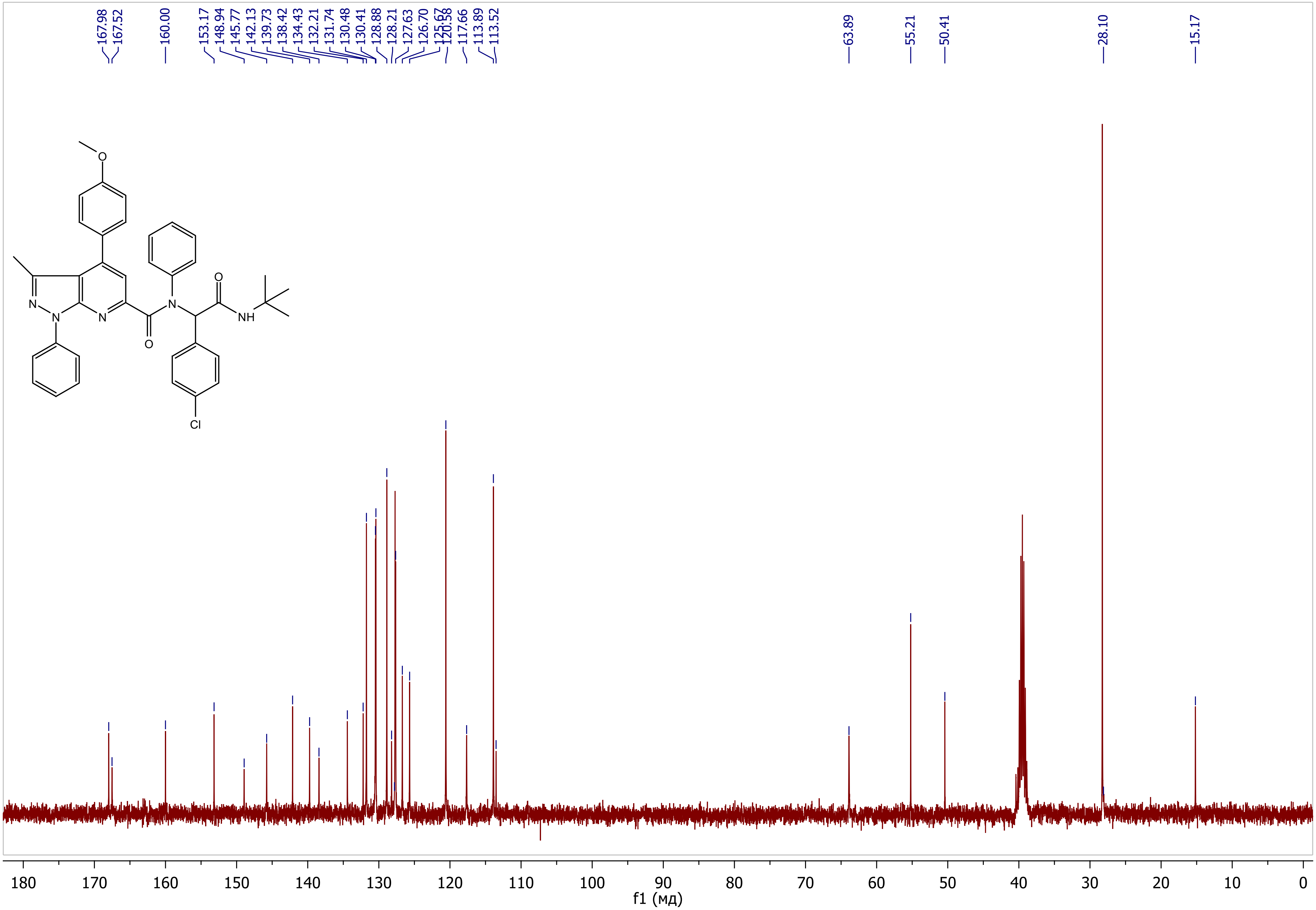
167.98
167.52
160.00
153.17
148.94
145.77
142.13
139.73
138.42
134.43
132.21
131.74
130.48
130.41
128.88
128.21
127.63
126.70
125.67
120.58
117.66
113.89
113.52
63.89
55.21
50.41
28.10
15.17
O
N
N
N
O
N
O
NH
Cl
180 170 160 150 140 130 120 110 100 90 80 70 60 50 40 30 20 10 0
f1 (мд)

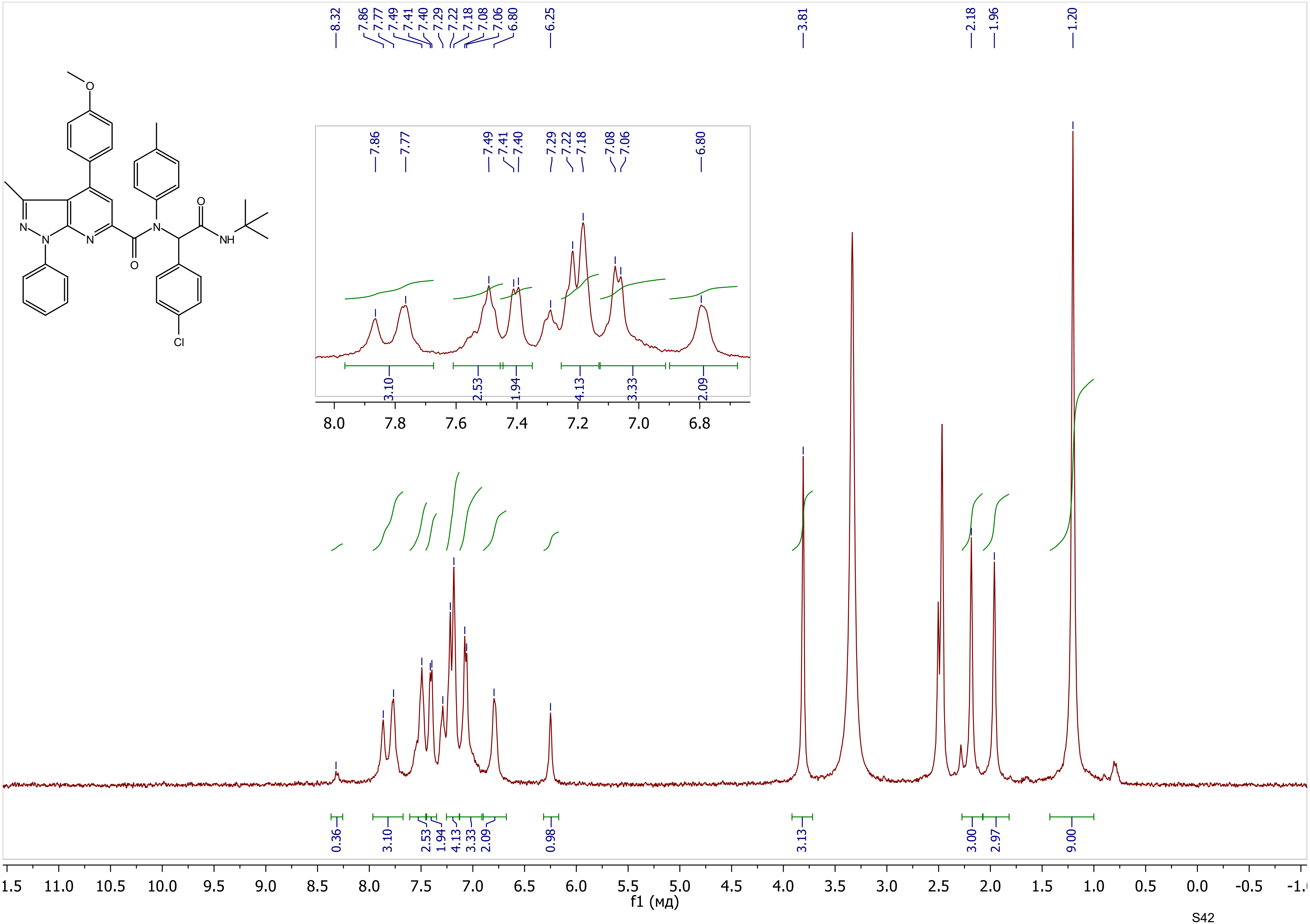
8.32
7.86
7.77
7.49
7.41
7.40
7.29
7.22
7.18
7.08
7.06
6.80
6.25
3.81
2.18
1.96
1.20
O
N
N
N
O
N
O
NH
Cl
0.36
3.10
2.53
1.94
4.13
3.33
2.09
0.98
3.13
3.00
2.97
9.00
f1 (мд)

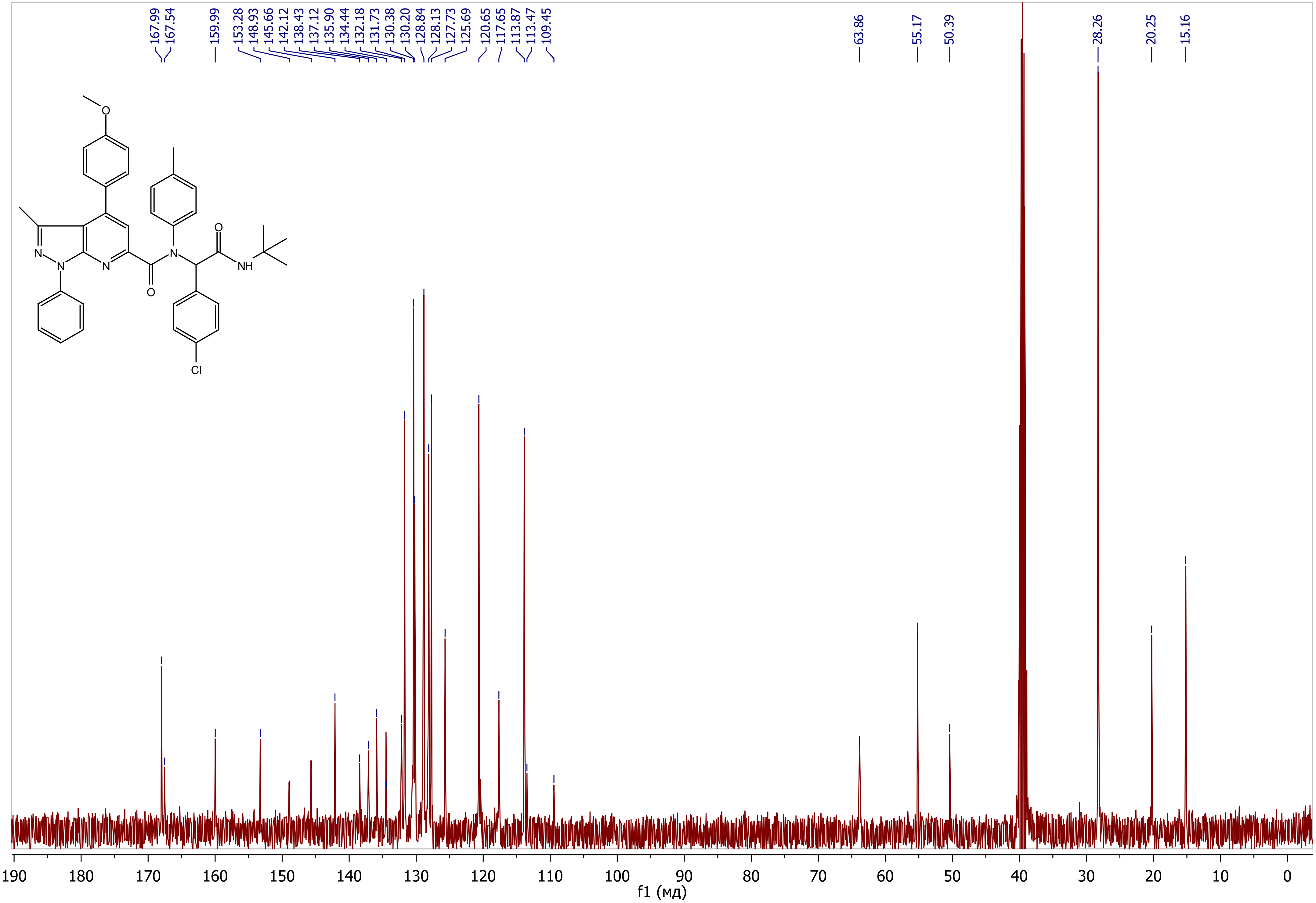
167.99
167.54
159.99
153.28
148.93
145.66
142.12
138.43
137.12
135.90
134.44
132.18
131.73
130.38
130.20
128.84
128.13
127.73
125.69
120.65
117.65
113.87
113.47
109.45
63.86
55.17
50.39
28.26
20.25
15.16
O
N
N
N
N
O
N
O
NH
Cl
190
180
170
160
150
140
130
120
110
100
90
80
70
60
50
40
30
20
10
0
f1 (мд)

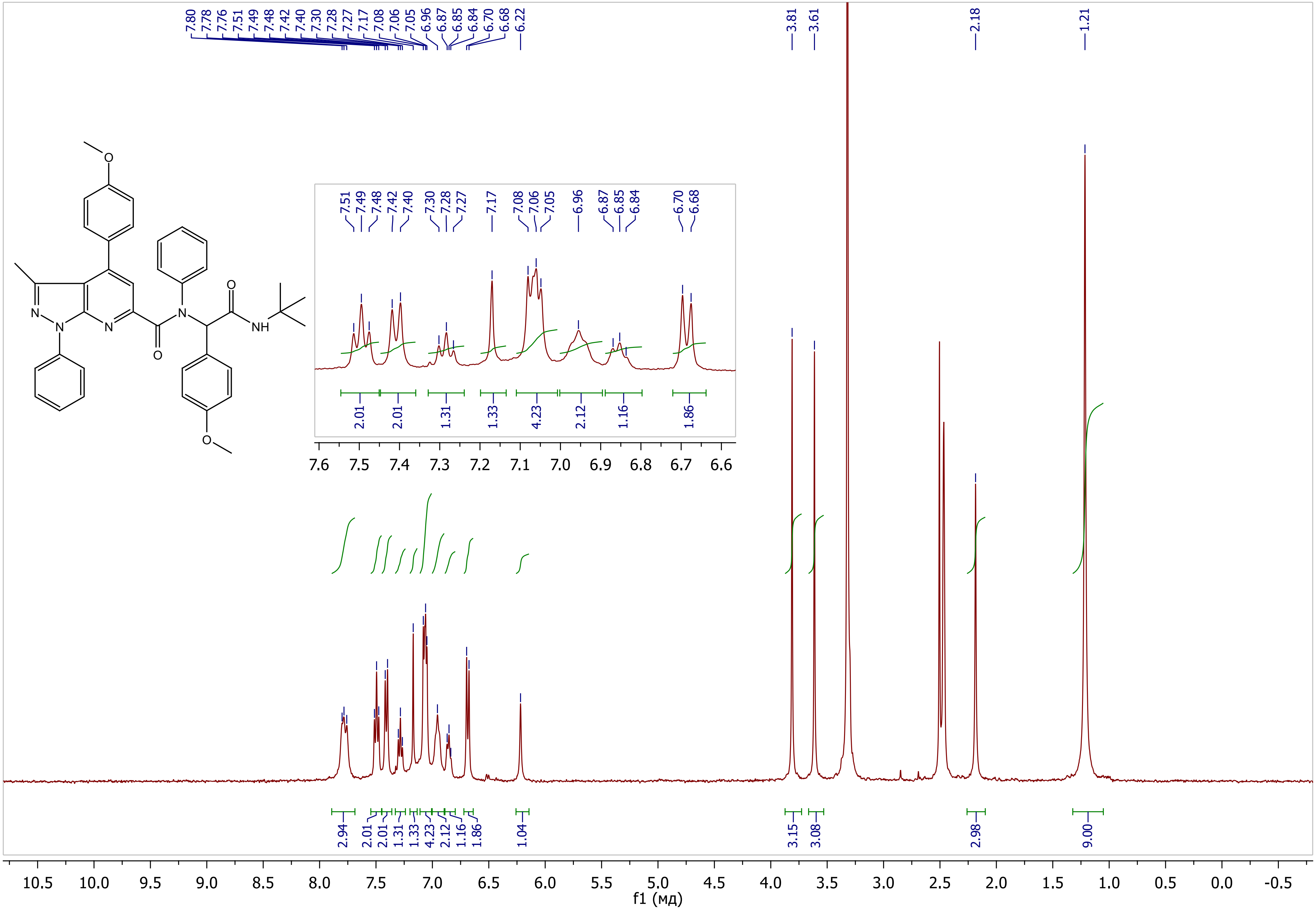
7.80
7.78
7.76
7.51
7.49
7.48
7.42
7.40
7.30
7.28
7.27
7.17
7.08
7.06
7.05
6.96
6.87
6.85
6.84
6.70
6.68
6.22
3.81
3.61
2.18
1.21
2.94
2.01
2.01
1.31
1.33
4.23
2.12
1.16
1.86
1.04
3.15
3.08
2.98
9.00
O
N
N
N
O
N
O
NH
O
10.5 10.0 9.5 9.0 8.5 8.0 7.5 7.0 6.5 6.0 5.5 5.0 4.5 4.0 3.5 3.0 2.5 2.0 1.5 1.0 0.5 0.0 -0.5
f1 (мд)
7.6 7.5 7.4 7.3 7.2 7.1 7.0 6.9 6.8 6.7 6.6

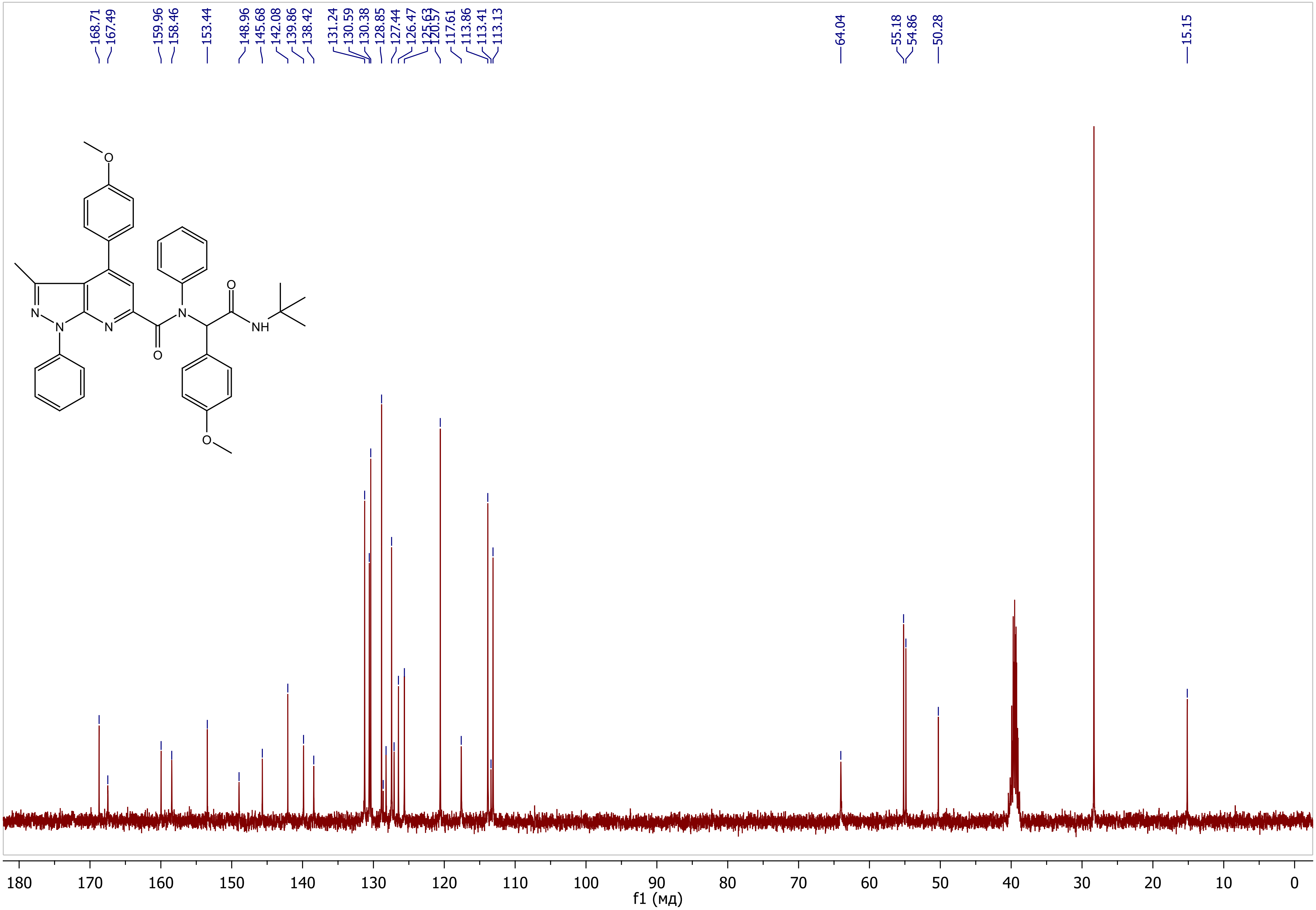

168.71
167.49
159.96
158.46
153.44
148.96
145.68
142.08
139.86
138.42
131.24
130.59
130.38
128.85
127.44
126.47
125.63
120.57
117.61
113.86
113.41
113.13
64.04
55.18
54.86
50.28
15.15
O
N
N
N
N
O
O
NH
O
180 170 160 150 140 130 120 110 100 90 80 70 60 50 40 30 20 10 0
f1 (мд)

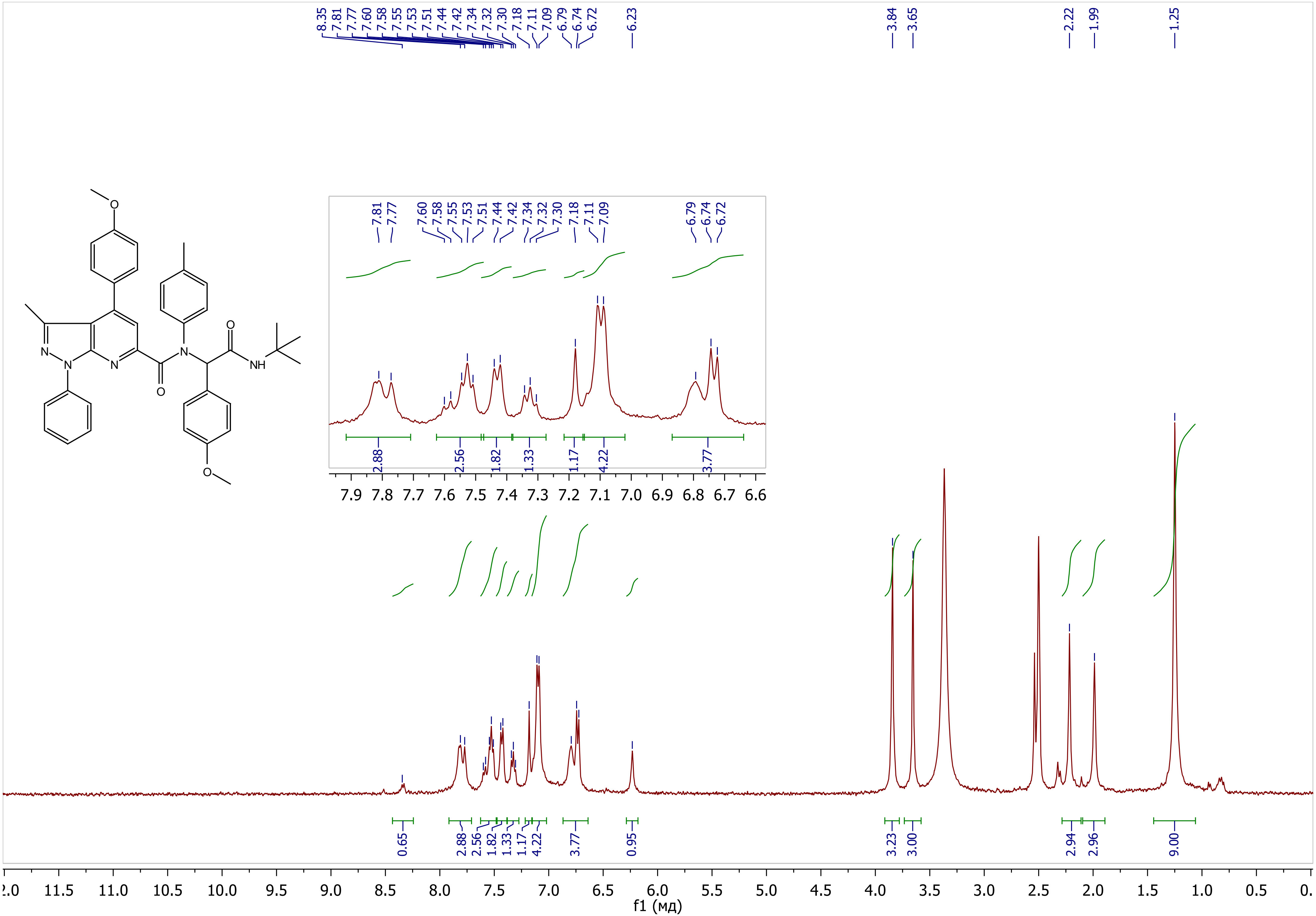
8.35 7.81 7.77 7.60 7.58 7.55 7.53 7.51 7.44 7.42 7.34 7.32 7.30 7.18 7.11 7.09 6.79 6.74 6.72 6.23 3.84 3.65 2.22 1.99 1.25
0.65 2.88 2.56 1.82 1.33 1.17 4.22 3.77 0.95 3.23 3.00 2.94 2.96 9.00
O
N
N
N
O
N
O
NH
O
f1 (мд)

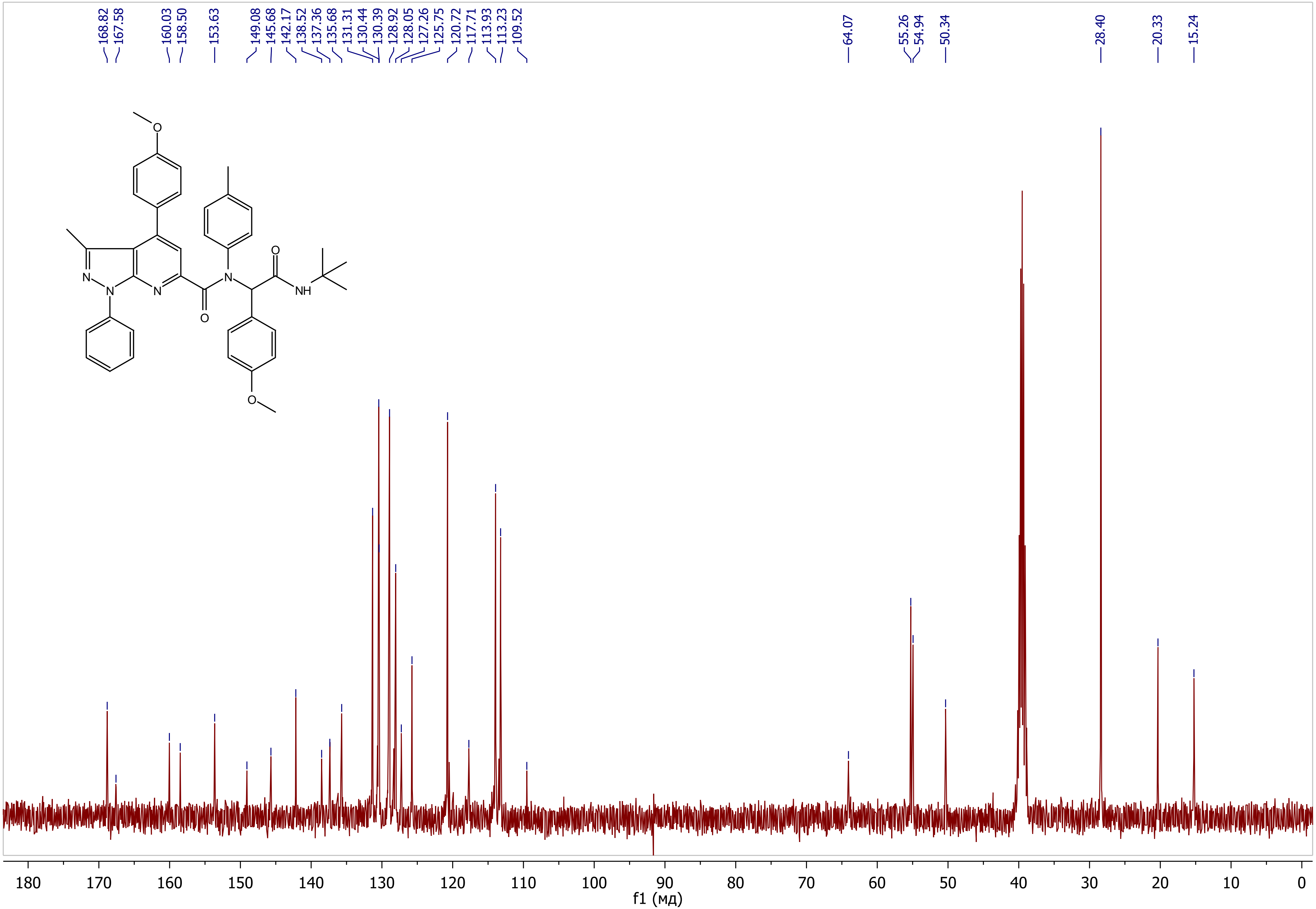
168.82
167.58
160.03
158.50
153.63
149.08
145.68
142.17
138.52
137.36
135.68
131.31
130.44
130.39
128.92
128.05
127.26
125.75
120.72
117.71
113.93
113.23
109.52
64.07
55.26
54.94
50.34
28.40
20.33
15.24
N
N
N
O
O
N
O
NH
O
O
180 170 160 150 140 130 120 110 100 90 80 70 60 50 40 30 20 10 0
f1 (мд)
